# MOTS-c is a mitochondrial-encoded interferon-linked host defense peptide

**DOI:** 10.7554/eLife.87615

**Published:** 2026-08-18

**Authors:** Michelle C Rice, Maria Imun, Sang Wun Jung, Chan Yoon Park, Jessica S Kim, Rochelle W Lai, Casey R Barr, Jyung Mean Son, Kathleen Tor, Emmeline Kim, Ryan J Lu, Ilana Cohen, Bérénice A Benayoun, Changhan Lee

**Affiliations:** 1 Leonard Davis School of Gerontology, University of Southern California Los Angeles United States; 2 USC Earth Sciences Los Angeles United States; 3 USC Norris Comprehensive Cancer Center Los Angeles United States; 4 USC Stem Cell Initiative Los Angeles United States; 5 Molecular and Computational Biology Department, USC Dornsife College of Letters, Arts and Sciences Los Angeles United States; 6 Biochemistry and Molecular Medicine Department, USC Keck School of Medicine Los Angeles United States; 7 Biomedical Science, Graduate School, Ajou University Suwon Republic of Korea; Singapore Immunology Network Singapore; National Institute of Immunology India

**Keywords:** MOTS-c, microprotein, host defense peptide, mitochondria, macrophage, mitochondrial immunity, Human, Mouse

## Abstract

The mitochondrial DNA (mtDNA) can trigger immune responses and directly entrap pathogens, but it is not known to encode active immune factors. The immune system is traditionally thought to be exclusively nuclear-encoded. Here, we report the identification of a host defense peptide (HDP) encoded in the human mitochondrial genome that presumably derives from the primordial proto-mitochondrial bacteria. We demonstrate that MOTS-c (mitochondrial open reading frame from the 12 S rRNA type-c) is a mitochondrial-encoded amphipathic and cationic peptide with direct antibacterial and immunomodulatory functions, consistent with the peptide chemistry and functions of known HDPs. MOTS-c targeted *Escherichia coli* and methicillin-resistant *Staphylococcus aureus* (MRSA), in part, by targeting their membranes using its hydrophobic and cationic domains. In a mouse model of acute peritonitis, MOTS-c fully neutralized MRSA infectivity. In human monocytes, interferon gamma (IFNγ), lipopolysaccharides (LPS), and differentiation signals each induced the expression of endogenous MOTS-c. Notably, exogenous MOTS-c, applied during primary mouse monocyte differentiation, reprogrammed the cells into macrophages with distinct transcriptomic signatures related to antigen presentation and IFN signaling. MOTS-c-programmed macrophages exhibited enhanced bacterial clearance and shifted metabolism. Our findings support MOTS-c as a first-in-class mitochondrial-encoded HDP and indicate that our immune system is not only encoded by the nuclear genome but also by the co-evolved mitochondrial genome.

## Introduction

The endosymbiotic theory posits that mitochondria originate from once free-living bacteria that infected eukaryotic ancestral cells. Indeed, owing to their bacterial origin (Martijn et al., 2018), mitochondria still retain several prokaryotic characteristics, including N-formylated proteins and circular DNA, that register as quasi-self damage-associated molecular patterns (DAMPs) (West and Shadel, 2017). Multiple pattern-recognition receptors can sense DAMPs and elicit pro-inflammatory and type I interferon responses (West and Shadel, 2017). Further, mitochondrial DNA (mtDNA) can be actively released by neutrophils (Papayannopoulos, 2018) and eosinophils (Yousefi et al., 2008) to physically target pathogens and by lymphocytes to signal type I interferon responses (Ingelsson et al., 2018).

While mtDNA itself can trigger immune responses and directly entrap pathogens, it is not known to encode for genes that yield immune factors. The continuous expansion of our proteome, powered by the characterization of short/small open reading frames (sORFs) in the nuclear (Chen et al., 2020; Saghatelian and Couso, 2015) and mitochondrial (Lee et al., 2013; Kim et al., 2017) genomes that yield functional peptides, provides unprecedented opportunities for the discovery of host defense peptides (HDPs), also known as antimicrobial peptides (AMPs) (Hancock et al., 2016; Mookherjee et al., 2020). All kingdoms of life possess a vast repertoire of AMPs that are now pursued therapeutically as antibiotics, antivirals, cell-penetrating peptides, immunomodulators, and cancer-targeting peptides (Hancock et al., 2016; Zhang and Gallo, 2016; Lewis, 2013; Zasloff, 2002; Hanson et al., 2019; Sassone-Corsi et al., 2016; Thomas et al., 2010; Lazzaro et al., 2020). HDPs are microproteins of 10–50 amino acids in length (Hancock et al., 2016; Zhang and Gallo, 2016; Zasloff, 2002). We have previously identified a mitochondrial-encoded sORF, MOTS-c (mitochondrial ORF from the 12 S rRNA type-c), which yields a 16 amino acid peptide (Lee et al., 2015). MOTS-c has a key role in regulating cellular homeostasis under cellular stress and during aging (López-Otín et al., 2023), in part, by directly translocating to the nucleus to regulate adaptive gene expression (Lee et al., 2015; Kim et al., 2018; Kang et al., 2021; Kong et al., 2021; Reynolds et al., 2021).

**Figure 1.**
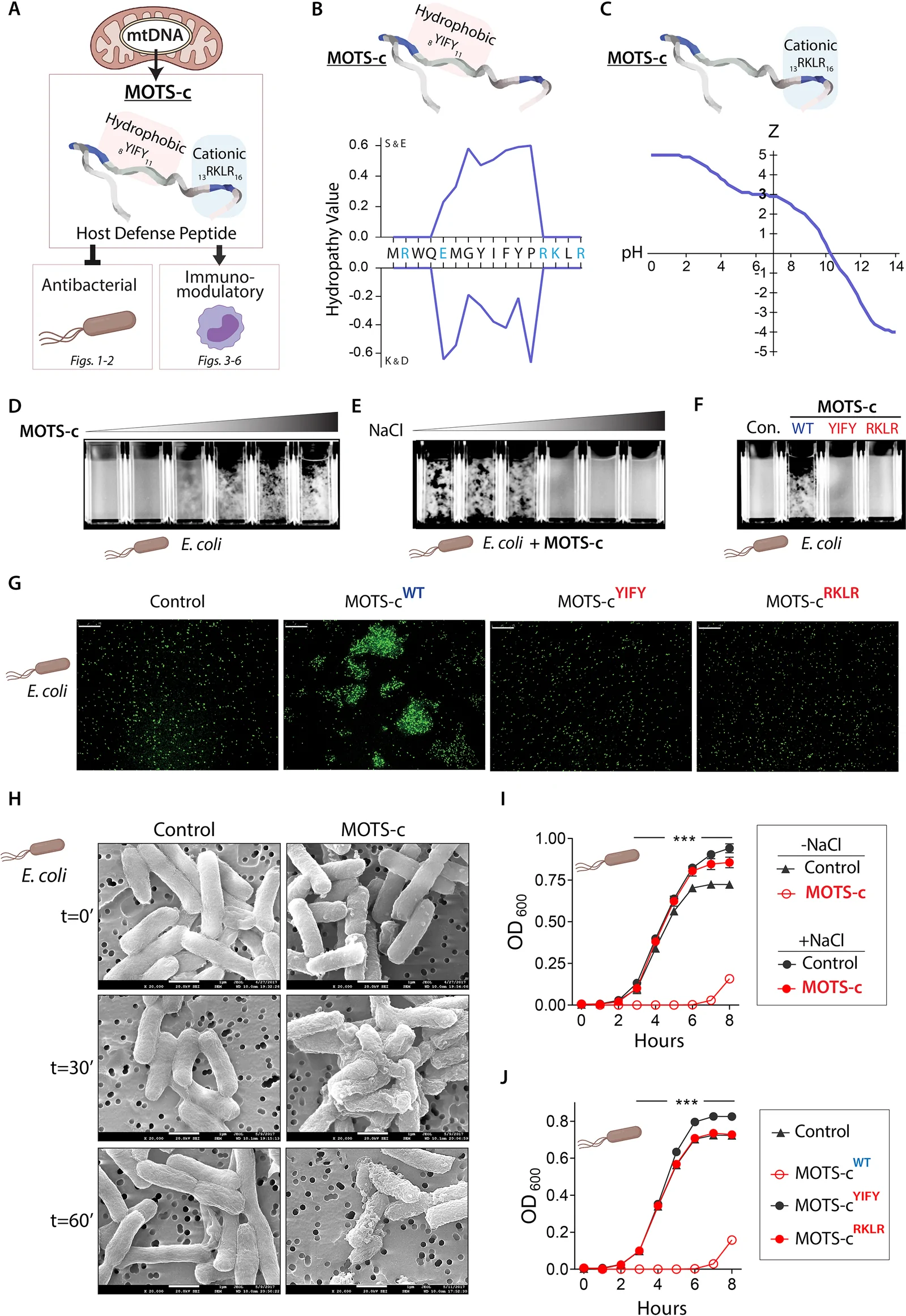
MOTS-c is a mitochondrial-encoded host defense peptide (HDP). (**A**) Bacteria and bacteria-derived mitochondria possess gene-encoded immune peptides, known as HDPs in higher eukaryotes. (**B**) MOTS-c has a hydrophobic core (_8_YIFY_11_), determined using the hydrophobicity scales of Kyte and Doolittle, 1982, and Sweet and Eisenberg, 1983. Blue: cationic residues. (**C**) MOTS-c has a cationic tail (_13_RKLR_16_) that confers positive charge (**Z**) across a pH range. (**D**) MOTS-c treatment (0–100 µM) immediately aggregates *E. coli* in a dose-dependent manner. (**E**) MOTS-c-dependent *E. coli* aggregation is lost in increasing salt concentrations (NaCl; 0–1%) and (**F, G**) requires its hydrophobic core and cationic tail, consistent with other HDPs. EGFP-expressing *E. coli* (BL21) is shown. Wild-type MOTS-c (WT) and mutants devoid of its hydrophobic (YIFY: _8_YIFY_11_>_8_AAAA_11_) or cationic domain (RKLR: _13_RKLR_16_>_13_AAAA_16_). Bar, 75 µm. (**H**) Scanning electron micrographs of *E. coli* treated with MOTS-c (100 µM) for 0 (immediate fixation), 30, and 60 min (n=3). Representative images are shown. Bar, 100 nm. (**I–J**) Growth curve of *E. coli* (BL21), measured by optical density at 600 nm (OD_600_), following (n=6) (**I**) MOTS-c treatment in the presence of 1% NaCl, and (**J**) treatment with wild-type (WT) MOTS-c and mutants devoid of its hydrophobic (_8_YIFY_11_>_8_AAAA_11_; YIFY), or cationic domain (_13_RKLR_16_>_13_AAAA_16_; RKLR). Data are expressed as mean ± SEM. Two-way ANOVA repeated measures. ***p<0.001.

Based on the endosymbiosis theory, early communication between the proto-mitochondria and proto-eukaryotic cell likely occurred on an immunological basis with immune factors that were encoded within both genomes. Today, bacteria still use gene-encoded peptides, known as bacteriocins, that regulate vital cellular processes (e.g. ribosomal processes, DNA replication, and cell wall synthesis; Le et al., 2017) to control inter- and intra-species proliferation (Sassone-Corsi et al., 2016; Monnet et al., 2016; Keller and Surette, 2006; Bassler, 2002; Cotter et al., 2013). In the unique endosymbiotic context, these peptides may have served to not only protect the newly formed union from other pathogens, but also to regulate the growth and metabolism of the opposing quasi-self (i.e. the present-day mitochondria and nucleus). Thus, mitochondrial-derived peptides (MDPs) (Miller et al., 2022), including MOTS-c, may inherently possess immuno-metabolic functions and represent a primordial arm of the eukaryotic immune system (Figure 1A) that evolved with dual roles as cellular regulators during aging (Lee et al., 2015; López-Otín et al., 2023; Reynolds, 2019).

**Figure 1—figure supplement 1.**
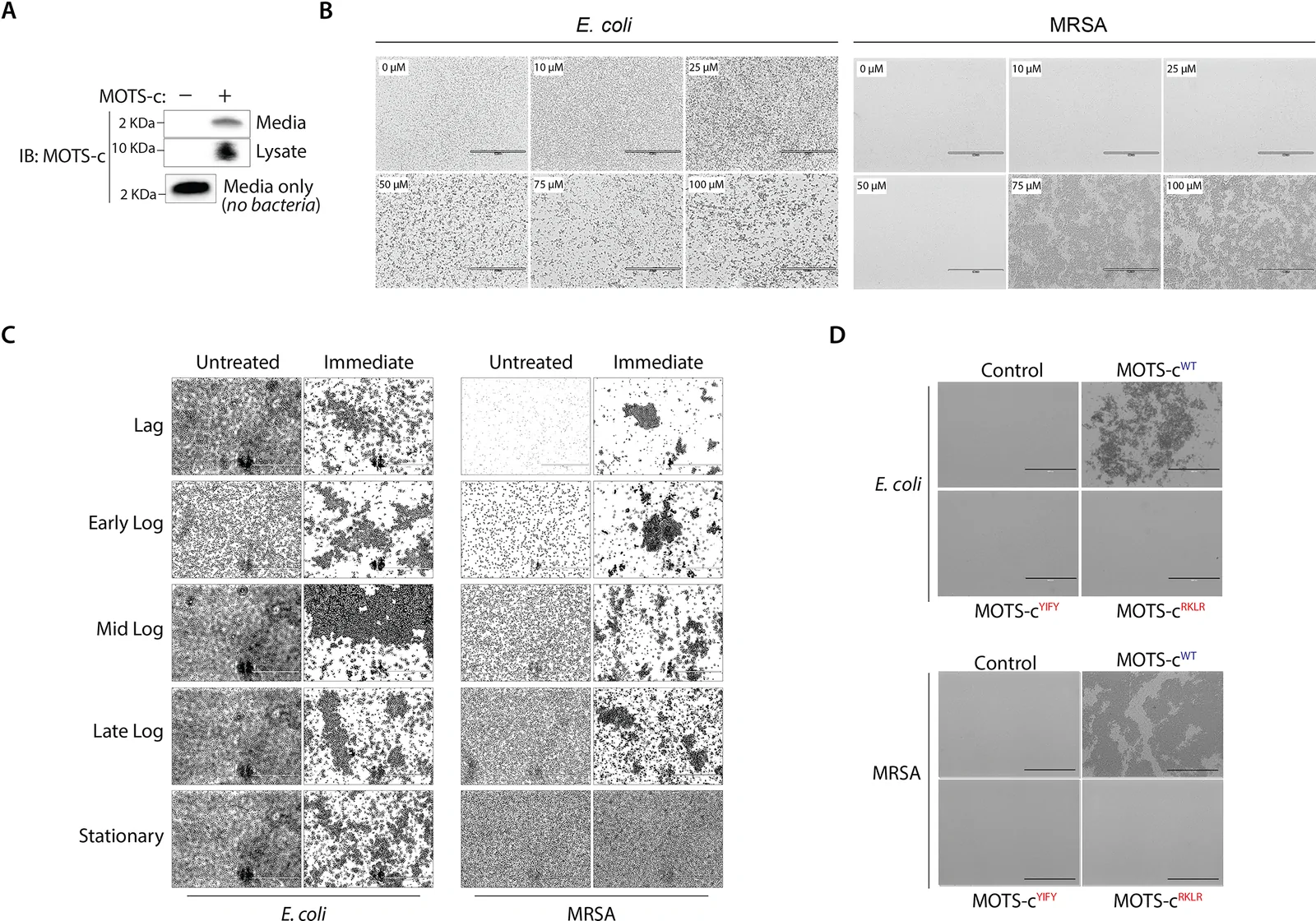
The effect of MOTS-c bacterial aggregation. (**A**) MOTS-c (100 μM) was added to an *E. coli* suspension in media (water), which immediately aggregated and precipitated bacteria. MOTS-c levels remaining in media (water) and associated with *E. coli* were detected by western blotting. MOTS-c (100 μM) levels in media (water) without any bacteria are also shown as reference. (**B**) MOTS-c immediately agglutinates bacteria in a dose-dependent manner. *E. coli* and methicillin-resistant *Staphylococcus aureus* (MRSA) were resuspended in water and treated with MOTS-c at varying doses (n=6). Bar, 200 μm. (**C**) *E. coli* and MRSA were collected at various points of growth (i.e. lag, early/mid/late log, and stationary phase), washed and resuspended in water, then treated with MOTS-c (100 μM). Images were taken immediately and rendered equally across all panels to aid in visual recognition of bacteria (n=3). Bar, 200 μm. (**D**) *E. coli* and MRSA were treated with wild-type (WT) or mutant MOTS-c (100 μM) and imaged immediately (n=6). MOTS-c mutants were devoid of its hydrophobic (_8_YIFY_11_>_8_AAAA_11_; MOTS-c^YIFY^) or cationic domain (_13_RKLR_16_>_13_AAAA_16_; MOTS-c^RKLR^). Bar, 400 μM. Figure 1—figure supplement 1—source data 1.PDF of uncropped western blots with relevant bands indicated. Figure 1—figure supplement 1—source data 2.Original western blot TIF files from ChemiDoc.

**Figure 1—figure supplement 2.**
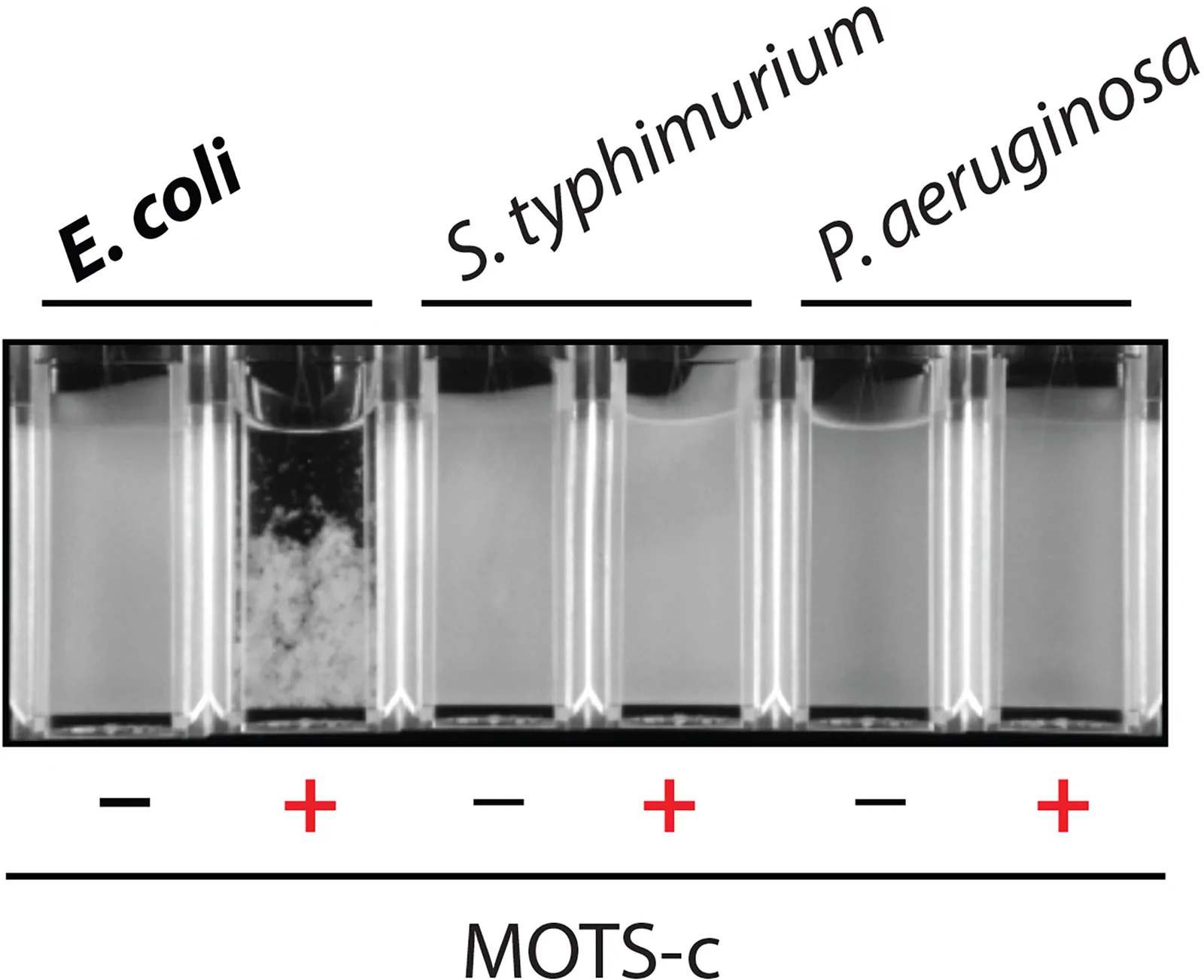
MOTS-c exhibits specificity in bacterial targeting. MOTS-c (100 μM) treatment causes immediate aggregation of *E. coli* but not *Salmonella typhimurium* or *Pseudomonas aeruginosa* (n=6). Representative image is shown. See Figure 1D.

**Figure 1—figure supplement 3.**
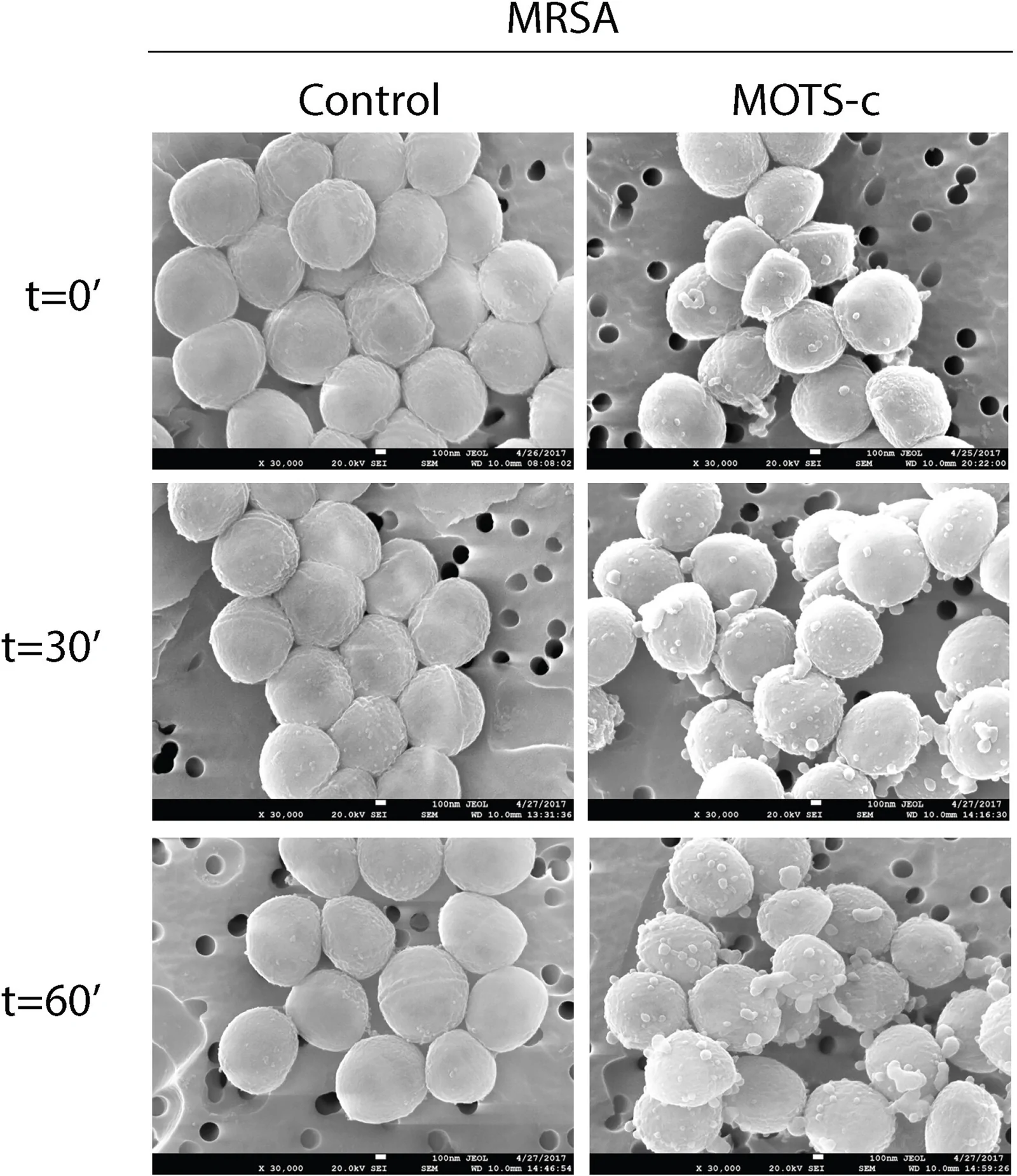
MOTS-c targets methicillin-resistant *S. aureus* (MRSA) membranes. Scanning electron micrographs of MRSA treated with MOTS-c (100 μM) for 0 (immediate fixation), 30, and 60 min (n=3). Representative images are shown. Bar, 100 nm.

**Figure 1—figure supplement 4.**
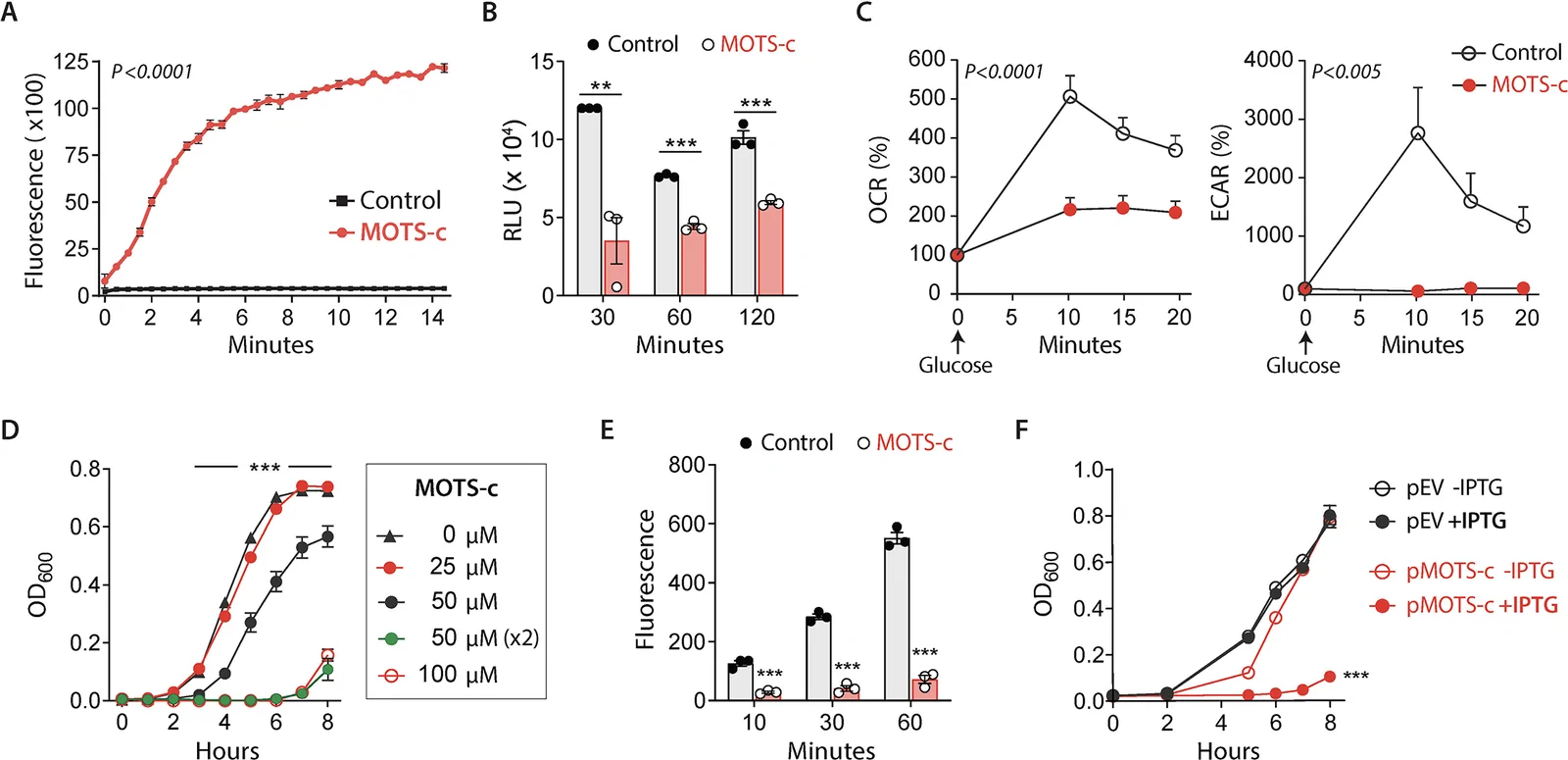
The effect of MOTS-c on bacterial metabolism and growth. (**A**) Membrane integrity assessment by time-course quantification of SYTOX Green nucleic acid stain, which is excluded by intact *E. coli* membranes (n=6). (**B**) Total cellular ATP levels measured following MOTS-c treatment (100 μM) in *E. coli* (n=3). RLU: relative light units. (**C**) Real-time metabolic assessment of *E. coli* respiration (oxygen consumption rate [OCR]) and glycolysis (extracellular acidification rate [ECAR]) (n=8). (**D**) Growth curve of *E. coli*, measured by optical density at 600 nm (OD_600_) following various doses of MOTS-c peptide treatment (n=6), (**E**) *E. coli* growth in M9 media with/without MOTS-c treatment (100 μM) was assessed using alamarBlue, a redox-sensitive resazurin dye (n=3). RFU: relative fluorescence units. (**F**) *E. coli* were transformed with expression vectors harboring the MOTS-c ORF (open reading frame) that can be induced by IPTG (isopropyl β-D-1-thiogalactopyranoside). Growth determined by optical density at 600 nm (OD_600_) periodically for 8 hr (n=3). Data expressed as mean ± SEM. Mann-Whitney test for (**B, E**) and two-way ANOVA (repeated measures) (A, C, D, and F). **p<0.01, ***p<0.001.

Here, we report, for the first time, that the human mitochondrial genome encodes for an HDP that directly targets bacteria and regulates monocyte function. This indicates that the human immune system is encoded in both of our co-evolved mitochondrial and nuclear genomes.

## Results

### The MDP MOTS-c compromises bacterial viability in vitro

HDPs are characterized by their cationic and amphipathic residues, typically with a net positive charge. Peptide analysis using the hydrophobicity scales of Kyte and Doolittle, 1982, and Sweet and Eisenberg, 1983, indicated that MOTS-c is an amphipathic peptide consisting of a core that is enriched with hydrophobic residues (_8_YIFY_11_) (Figure 1B). MOTS-c retains a +3 charge under physiological pH, largely owing to the basic tail residues (_13_RKLR_16_) (Figure 1C), which may also serve as a heparin-binding domain (XBBXB-like moiety; B: basic residues) that can bind to cell wall carbohydrates (e.g. glucan, mannan) and mediate bacterial recognition and binding (Andersson et al., 2004; Chang et al., 2017).

Direct bacterial targeting is a hallmark of HDPs (Lazzaro et al., 2020; Melo et al., 2009). Indeed, MOTS-c dynamically associated with bacteria immediately upon contact (Figure 1D–H and Figure 1—figure supplement 1). We mixed MOTS-c (100 µM) with *E. coli* suspended in water. MOTS-c levels in the media (water) decreased concomitantly with increased detection in cell lysates, indicating direct interaction with bacteria (Figure 1—figure supplement 1A). HDPs can cause bacterial aggregation, which immobilizes them to the local infection site and enhances pathogen clearance (Sinha et al., 2017; Pulido et al., 2012; Robert et al., 2015; Torrent et al., 2012; Chairatana and Nolan, 2014). Consistently, MOTS-c immediately aggregated *E. coli* and MRSA in a dose- and growth phase-dependent manner (Figure 1D and Figure 1—figure supplement 1B–C; Video 1). HDPs engage with bacteria through ionic and hydrophobic interactions, owing to their hydrophobic and charged residues (Bahar and Ren, 2013; Bals et al., 1998). MOTS-c was ineffective in aggregating bacteria under higher salt concentrations (0–1% in ddH_2_O) (Figure 1E), which disrupts ionic interactions and HDP activity (Bals et al., 1998; Kandasamy and Larson, 2006). The loss of the hydrophobic and cationic domains of MOTS-c, by substituting the residues with alanine (i.e. _8_YIFY_11_>_8_AAAA_11_ and _13_RKLR_16_>_13_AAAA_16_), prevented bacterial aggregation (Figure 1F–G and Figure 1—figure supplement 1D), consistent with the importance of these residues for HDP function (Hancock et al., 2016; Choi et al., 2016). MOTS-c did not aggregate *S. typhimurium* or *P. aeruginosa* (Figure 1—figure supplement 2)*,* indicating target selectivity independent of Gram status.

**Video 1.** Aggregation of *E. coli* by MOTS-c. A 1 ml aliquot of *E. coli* BL21 culture (OD_600_ ≈ 0.6) was pelleted by centrifugation at 6000×*g* and resuspended in ddH_2_O. MOTS-c was immediately added to a final concentration of 100 μM, and the suspension was vortex-mixed and transferred to a cuvette containing 1 ml of ddH_2_O for imaging.

HDPs can perturb bacterial membranes via several mechanisms, including progressive blebbing, budding, and pore formation (Schmidt et al., 2011; Schmidt and Wong, 2013; Farkas et al., 2017). Using scanning electron microscopy (SEM), we visualized a time-dependent progression of MOTS-c-dependent membrane blebbing in *E. coli* (Figure 1H) and MRSA (Figure 1—figure supplement 3). Membrane destabilization by HDPs can also cause bacterial aggregation (Torrent et al., 2012; Sinha et al., 2017; Robert et al., 2015). We confirmed rapid compromise of bacterial membrane integrity upon MOTS-c treatment using a fluorescent nucleic acid stain that only penetrates permeabilized membranes (Roth et al., 1997; Figure 1—figure supplement 4A). Further, MOTS-c treatment depleted cellular ATP levels in *E. coli* (Figure 1—figure supplement 4B). Consistently, real-time metabolic flux analyses revealed that MOTS-c treatment perturbed respiration and glycolysis (Figure 1—figure supplement 4C), which requires intact membrane function (Hurdle et al., 2011).

We next confirmed the antimicrobial effect of MOTS-c on bacterial growth. A single treatment of MOTS-c significantly retarded *E. coli* proliferation in liquid culture in a dose-dependent manner (Figure 1—figure supplement 4D–E). Two intermediate doses of MOTS-c (50 µM) had comparable effects to a single high-dose treatment (100 µM) (Figure 1—figure supplement 4D), reflecting the significance of antibiotic treatment frequency in addition to absolute dose. Notably, human HDPs can reach high intracellular concentrations compared to their low circulating levels: LL-37 can reach 40 µM (Lai and Gallo, 2009; Sørensen et al., 1997; Sørensen et al., 2001; Duplantier and van Hoek, 2013) and defensins can constitute 5–7% of the total protein content of neutrophils (Ashrafi et al., 2017; Lehrer and Ganz, 1992). Further, HDPs can reach very high membrane-bound concentrations that are 10,000 times that of aqueous solutions (80 mM) (Melo et al., 2009). Interruption of ionic interaction, achieved by higher salt concentration (Figure 1I) or loss of the hydrophobic or cationic domains by alanine-substitution mutagenesis (i.e*.*_8_YIFY_11_>_8_AAAA_11_ and _13_RKLR_16_>_13_AAAA_16_; 100 µM) (Figure 1J), fully reversed the antimicrobial function of MOTS-c on *E. coli* growth. Multiple HDPs also have intracellular roles that contribute to the antimicrobial effect (Nicolas, 2009; Cudic and Otvos, 2002; Shah et al., 2016; Ho et al., 2016). Using an inducible MOTS-c expression vector, we found that the endogenous overexpression of MOTS-c significantly inhibited *E. coli* (BL21) growth (Figure 1—figure supplement 4F), indicating a two-stage mechanism of targeting membranes and intracellular components.

### MOTS-c enhances survival from MRSA exposure in vivo

**Figure 2.**
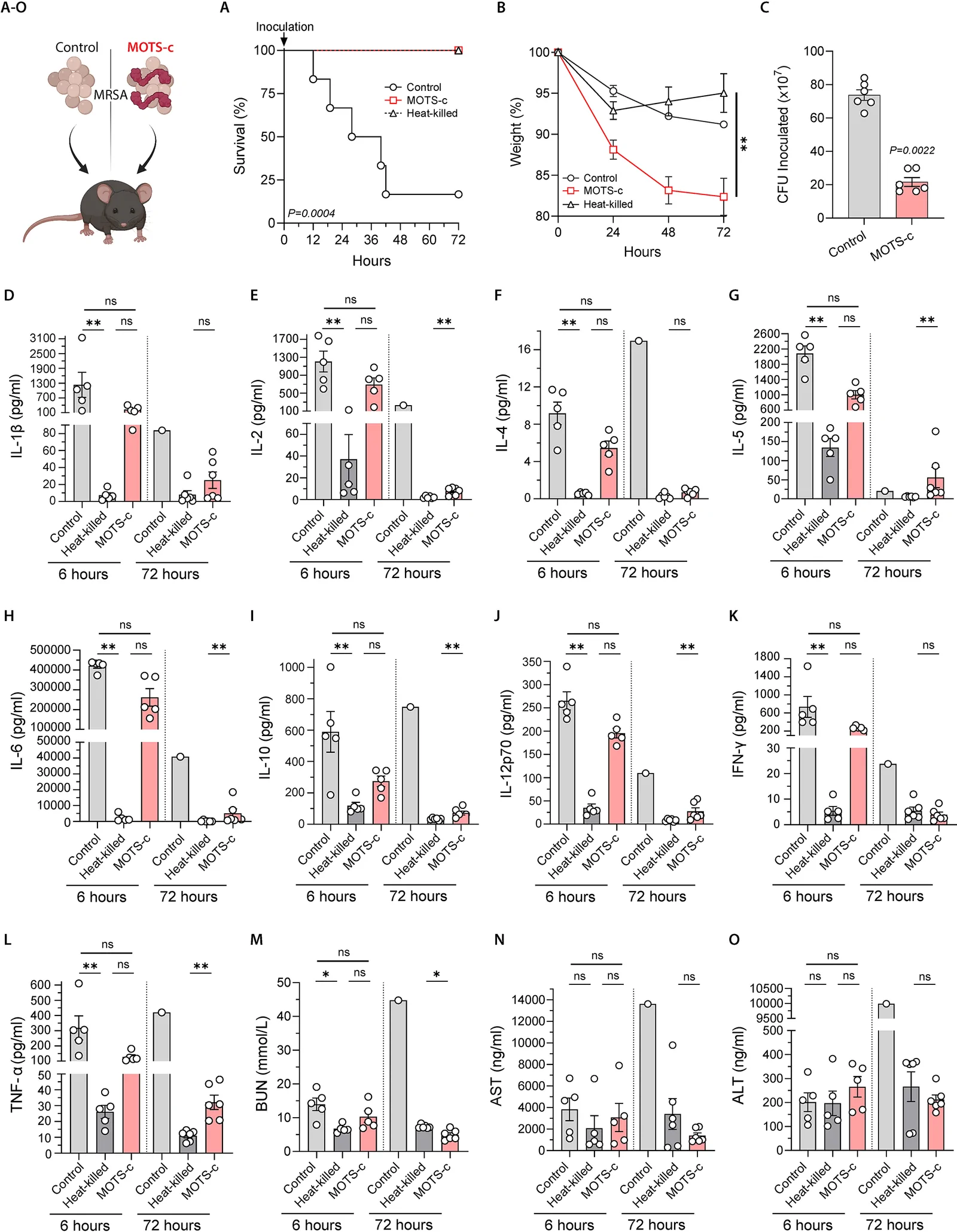
MOTS-c enhances survival from methicillin-resistant *S. aureus* (MRSA) exposure in vivo. (**A–O**) 6×10^8^ colony-forming unit (CFU) of mid-log phase MRSA either resuspended in (**i**) 100 µM MOTS-c, (ii) vehicle (water), or (iii) in vehicle and then heat-killed in a water bath. MRSA preparations were then immediately injected IP into 6-month-old female C57BL/6J mice (n=5–6). Mice were euthanized and blood collected after 6 and 72 hr. (**A**) Survival and (**B**) weight were monitored for 72 hr. (**C**) 6×10^8^ CFU of MRSA resuspended in 100 µM MOTS-c or water was serially diluted and plated on LB agar before injection and colonies counted after overnight incubation. (**D–L**) Cytokines (IL-1β, IL-2, IL-4, IL-5, IL-6, IL-10, IL-12p70, IFN-γ, and TNF-α) were measured in plasma by multiplex ELISA after 6 hr (n=5) and 72 hr (n=1 for control and n=6 for others; surviving mice). Plasma levels of (**M**) blood urea nitrogen (BUN), (**N**) aspartate transaminase (AST), and (**O**) alanine transaminase (ALT) were measured by ELISA at 6 hr (n=5) and 72 hr (n=1 for control and n=6 for others). Data are expressed as mean ± SEM. Log-rank (Mantel-Cox) test for (**A**), two-way ANOVA (repeated measures) for (**B**), Mann-Whitney test for (**C**), and Kruskal-Wallis test (6 hr time point) and Mann-Whitney test (72 hr time point; due to only one control surviving) for (**D–O**). *p<0.05, **p<0.01.

To confirm the sustained antibacterial effects of MOTS-c in vivo, we inoculated female mice with MRSA that had been treated with or without MOTS-c (Figure 2A–O). While intraperitoneal inoculation of MRSA was lethal, MOTS-c-treated MRSA was not (16.67% vs. 100% survival, respectively) (Figure 2A). To further explore whether lethal peritonitis could be induced by high levels of pathogen-associated molecular patterns alone or if live bacteria were necessary, we simultaneously inoculated a third group of mice with heat-killed MRSA. All mice in this group survived, indicating that live bacteria are required to cause lethal peritonitis (Figure 2A). Notably, both the heat-killed MRSA and MOTS-c-treated MRSA groups showed complete survival; however, the group that received MOTS-c-treated MRSA lost weight during the 72 hr infection, suggesting a distinct response to the inoculation (Figure 2B). MOTS-c-treated MRSA, sampled from the inoculated preparation (6×10^8^ CFU), showed a 3.4-fold reduction in CFU on LB agar plates (Figure 2C). This suggests that treatment with MOTS-c reduced the MRSA bacterial burden sufficiently for the mice to clear the infection and recover. To determine the level of inflammatory response to MRSA inoculation, we measured circulating cytokines (IL-1β, IL-2, IL-4, IL-5, IL-6, IL-10, IL-12p70, IFN-γ, and TNF-α) 6 hr post-infection. Indeed, mice inoculated with MOTS-c-treated MRSA had an intermediary induction of cytokines between those of the control and heat-killed MRSA groups (Figure 2D–L). At 72 hr post-infection, cytokine levels in the MOTS-c-treated MRSA group were significantly reduced and closer to those of the heat-killed group compared to the control group (sole surviving mouse), indicating resolution of inflammation (Figure 2D–L). Elevated blood urea nitrogen (BUN), aspartate transaminase (AST), and alanine transaminase (ALT) levels in circulation indicate kidney and liver injury during lethal infection (Liao et al., 2013). After 72 hr, mice inoculated with MOTS-c-treated MRSA had lower BUN levels and no difference in AST and ALT levels compared to the heat-killed MRSA group (Figure 2M–O). This suggests that the MOTS-c-treated MRSA group induced an inflammatory immune response without significant organ damage. These results were consistent in male mice, with 20% vs. 100% survival in the control and MOTS-c-treated group, respectively, and a 19.8-fold reduction in CFU of sampled inoculated MRSA preparation (4×10^8^ CFU) on LB agar plates (Figure 2—figure supplement 1). This indicates that, similar to other known HDPs, the antimicrobial effects of MOTS-c depend on the stoichiometric ratio of MOTS-c:MRSA and their proximity (Malanovic and Lohner, 2016). These results suggest that MOTS-c can opsonize bacteria, perhaps for immune clearance, and neutralize their pathogenicity.

**Figure 2—figure supplement 1.**
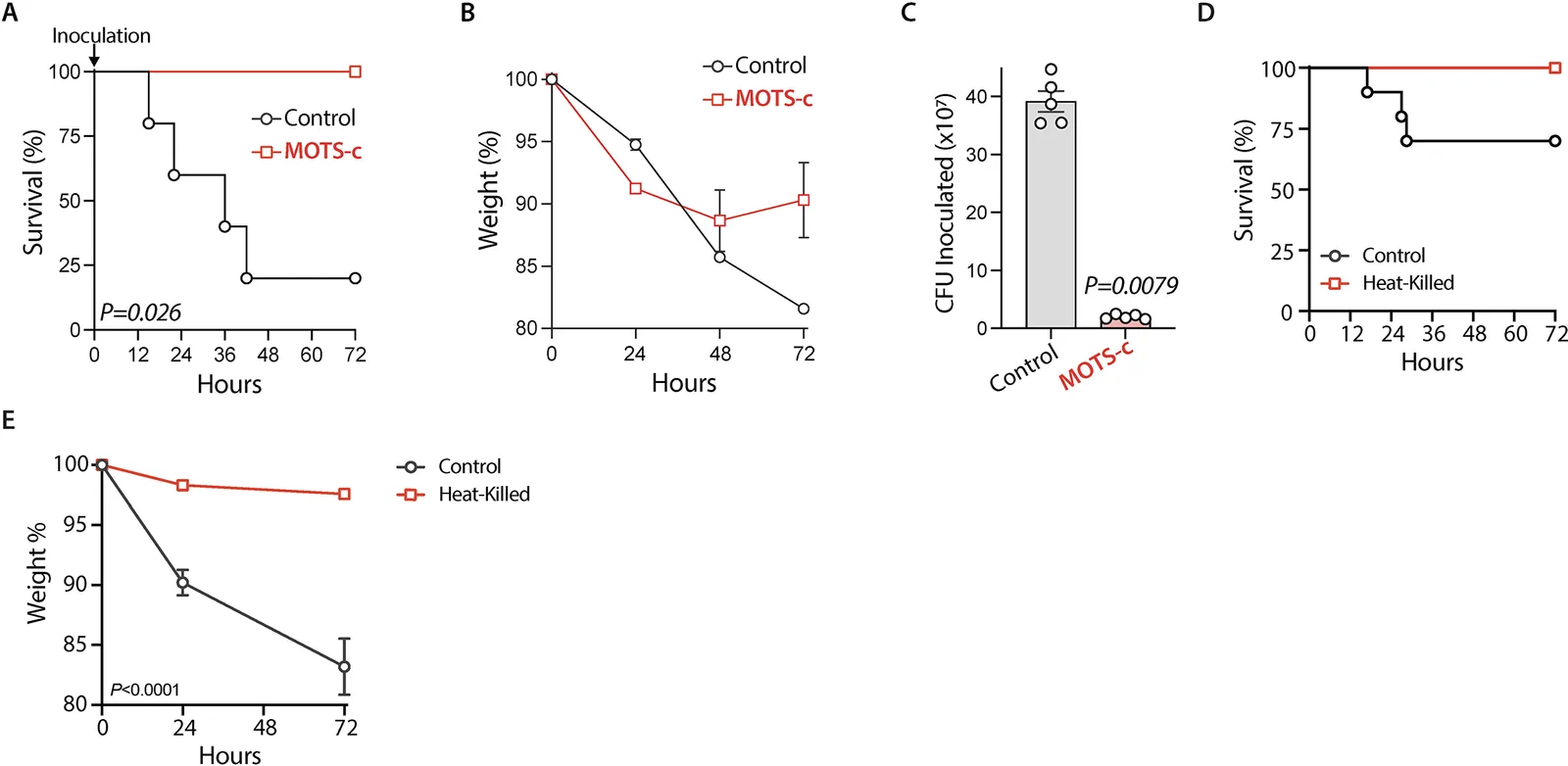
MOTS-c enhances survival from methicillin-resistant *S. aureus* (MRSA) exposure in vivo in male mice. (**A–C**) 4×10^8^ colony-forming unit (CFU) of mid-log phase MRSA was resuspended in 100 µM MOTS-c or vehicle (water) and immediately injected IP into 3.5- to 4-month-old male C57BL/6J mice (n=4–5). Survival (**A**) and weight (**B**) were monitored for 72 hr. (**C**) 4×10^8^ CFU of MRSA resuspended in 100 µM MOTS-c or water was serially diluted and plated on LB agar before injection and colonies counted after overnight incubation. (**D–E**) 4×10^8^ CFU of mid-log phase *S. aureus* (MRSA) was resuspended in phosphate-buffered saline (PBS) and either kept on ice (control) or heat-killed in a 70°C water bath for 20 min. Live or heat-killed MRSA was injected IP into 3.5- to 4-month-old male C57BL/6J mice (n=10). Survival (**D**) and weight (**E**) were monitored for 72 hr. Data expressed as mean ± SEM. Log-rank (Mantel-Cox) test (**A,D**), Mann-Whitney test (**C**), and two-way ANOVA (repeated measures) (**E**).

### Endogenous MOTS-c is induced upon human monocyte activation in vitro

**Figure 3.**
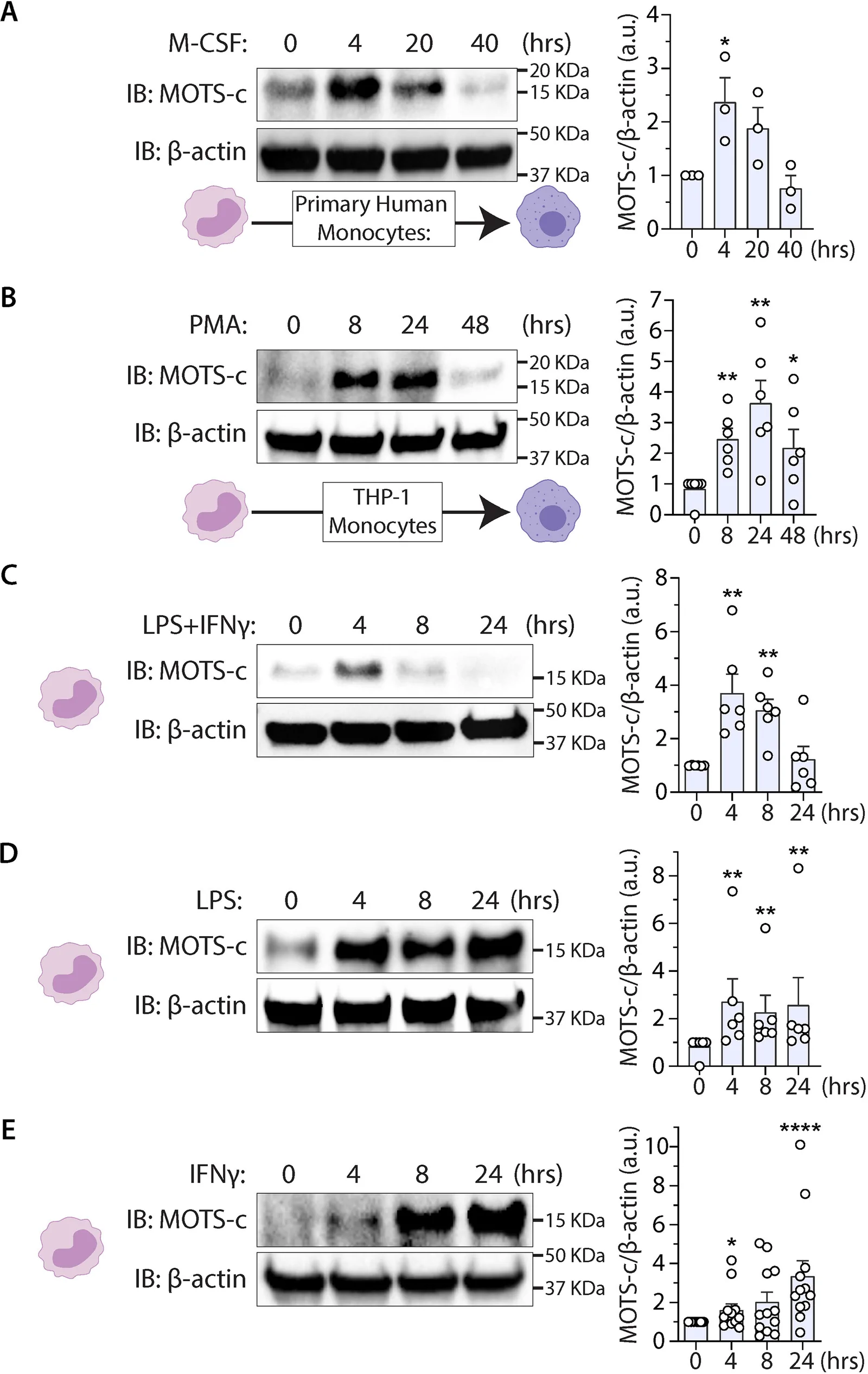
MOTS-c is induced in activated and differentiating monocytes. (**A–B**) Total endogenous MOTS-c levels measured as a function of time following monocyte differentiation in (**A**) primary human monocytes by macrophage colony-stimulating factor (M-CSF) (100 ng/ml) (n=3) and (**B**) THP-1 cells by phorbol myristate acetate (PMA) (15 nM) (n=6). (**C–E**) Total endogenous MOTS-c levels following THP-1 monocyte activation by lipopolysaccharides (LPS) (100 ng/ml) and interferon gamma (IFNγ) (20 ng/ml) (**C**) in combination (n=6), (**D**) LPS alone (n=6), and (**E**) IFNγ alone (n=12). Data are expressed as mean ± SEM. Mann-Whitney test. *p<0.05, **p<0.01, ****p<0.0001. Figure 3—source data 1.PDF of uncropped western blots with relevant bands indicated. Figure 3—source data 2.Original western blot TIF files from ChemiDoc.

Although the initial focus on HDP research was on their direct antimicrobial effect, recent studies have established them as key regulators of immune responses, including monocyte activation and differentiation (Hancock et al., 2016; Mookherjee et al., 2020). Because (i) the discovery of MOTS-c was inspired by a prior study demonstrating interferon gamma (IFNγ)-induced transcripts from the mitochondrial rRNA genes in monocytes (Tsuzuki et al., 1983) and (ii) MOTS-c regulates adaptive cellular responses to various types of stress (Lee et al., 2015; Kim et al., 2018; Kang et al., 2021; Kong et al., 2021; Reynolds et al., 2021), we hypothesized that it may act as a modulator of monocyte activation and differentiation. Endogenous MOTS-c levels dynamically increased in a time-dependent manner upon monocyte-to-macrophage differentiation in (i) primary human peripheral blood monocytes by M-CSF (macrophage colony-stimulating factor) (Figure 3A) and (ii) a human monocytic cell line (THP-1) by phorbol myristate acetate (PMA) (Auwerx, 1991; Figure 3B). Since they recapitulated the dynamics of MOTS-c upon differentiation signals and they are a tractable cell line, we decided to perform follow-up experiments primarily in the THP-1 system. Endogenous MOTS-c was also induced in THP-1 monocytes in a time-dependent manner following stimulation with LPS and IFNγ (Figure 3C), a combination of bacterial-derived and cytokine-dependent signals known to synergistically activate monocytes (Chow et al., 2014; Tamai et al., 2003; Duits et al., 2002; Müller et al., 2017; Held et al., 1999; Schroder et al., 2004; Nakagomi et al., 2000). We then tested whether LPS and IFNγ could each induce MOTS-c expression separately. LPS alone increased MOTS-c expression in THP-1 monocytes (Figure 3D) and differentiated THP-1 macrophages (Figure 3—figure supplement 1A), consistent with known HDPs (Fang et al., 2003; Pioli et al., 2006; Liu et al., 2003; Tsutsumi-Ishii and Nagaoka, 2003; Ayabe et al., 2000; Gläser et al., 2005; Harder et al., 1997; Sun et al., 2015; Zhai et al., 2018). IFNγ alone also induced MOTS-c expression in THP-1 monocytes (Figure 3E), consistent with the strong induction of transcripts from the mitochondrial rRNA genes in interferon-induced monocyte-like cells (Tsuzuki et al., 1983). Cellular levels of induced MOTS-c in THP-1 macrophages appear to reach high concentrations (Figure 3—figure supplement 1B), consistent with known HDPs (Lai and Gallo, 2009; Sørensen et al., 1997; Sørensen et al., 2001; Duplantier and van Hoek, 2013; Ashrafi et al., 2017; Lehrer and Ganz, 1992).

**Figure 3—figure supplement 1.**
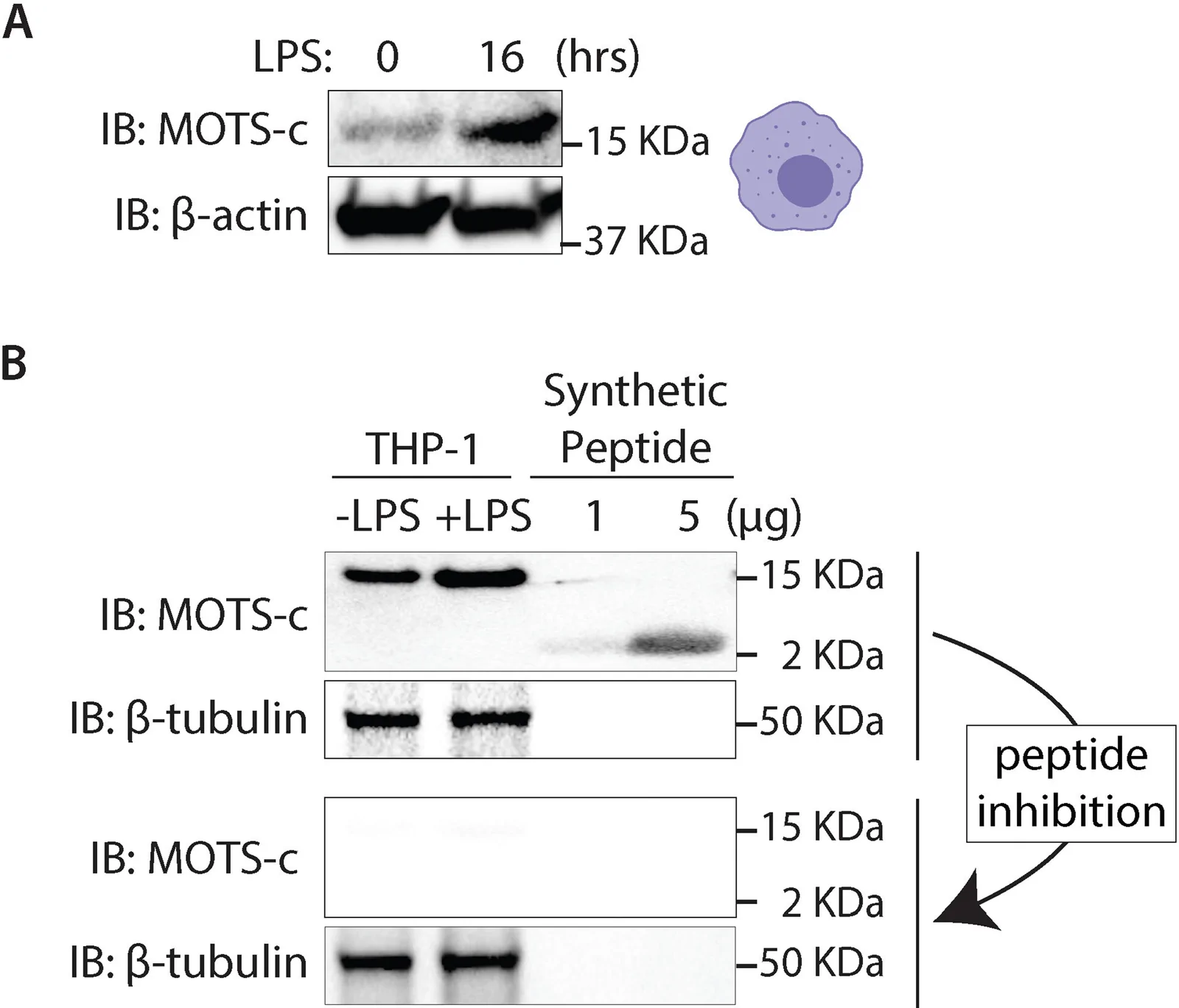
Endogenous MOTS-c levels in macrophages. (**A**) Total endogenous MOTS-c levels in differentiated macrophages (THP-1) measured following lipopolysaccharides (LPS) treatment for 16 hr. (**B**) Comparison of endogenous MOTS-c levels in macrophages (THP-1; 15 μg total cell lysates/well) and measured synthetic peptide levels (1 and 5 μg). Antibodies were competed out with MOTS-c peptide to confirm detection specificity. Synthetic MOTS-c peptide was chemically synthesized without any post-translational modifications (PTM) or oligomerizations. Figure 3—figure supplement 1—source data 1.PDF of uncropped western blots with relevant bands indicated. Figure 3—figure supplement 1—source data 2.Original western blot TIF files from ChemiDoc.

### MOTS-c regulates the differentiation trajectory of human monocytes in vitro

**Figure 4.**
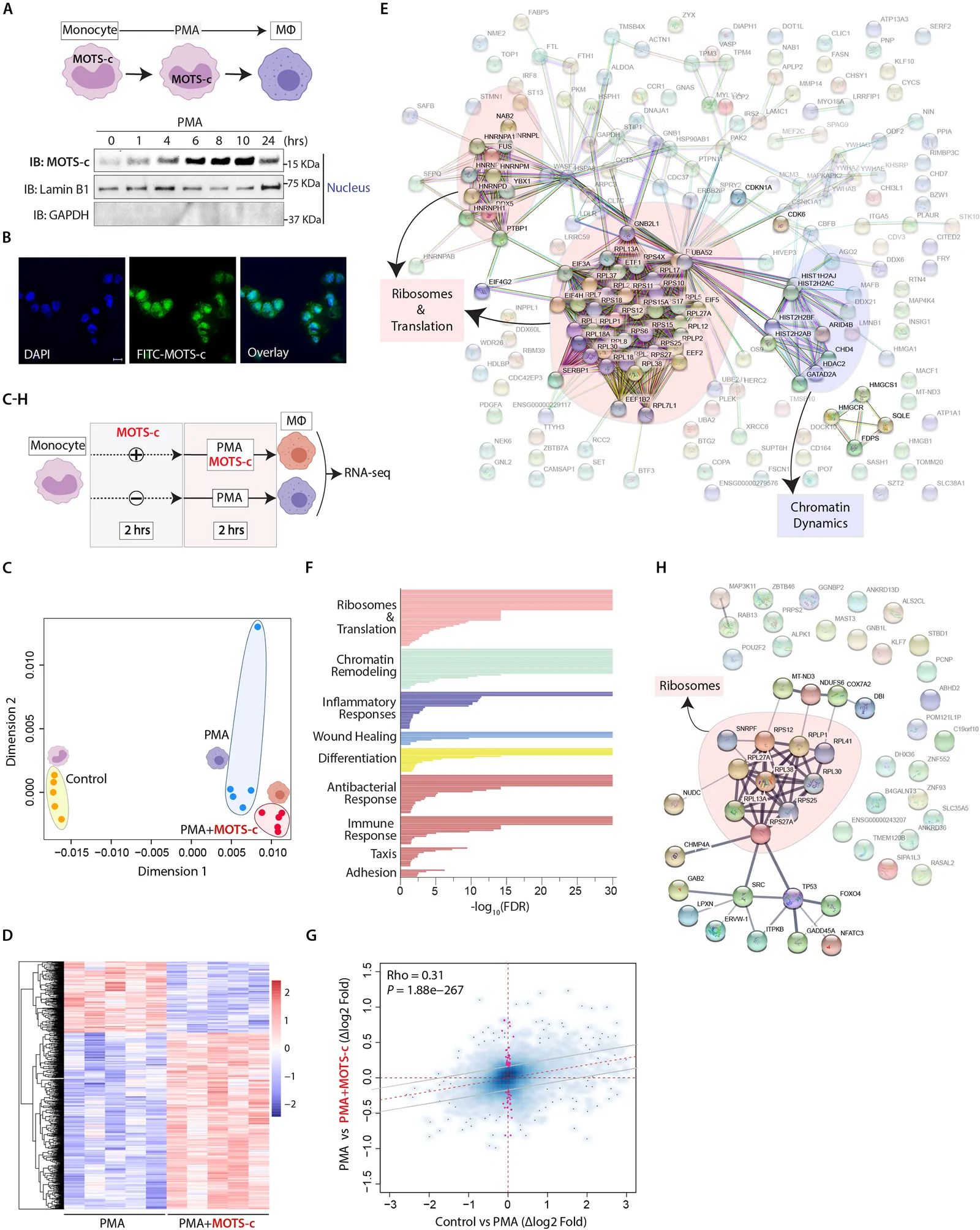
MOTS-c reprograms early nuclear gene expression during monocyte differentiation. (**A**) A time-course measurement of endogenous MOTS-c in purified nuclear extracts following THP-1 monocyte differentiation by phorbol myristate acetate (PMA) (15 nM). (**B**) Confocal fluorescence images of THP-1 monocytes treated with FITC-MOTS-c (1 µM) for 30 min, showing nuclear localization. Nucleus marked by DAPI staining. Bar, 10 µm. (**C–H**) THP-1 monocytes were primed with MOTS-c (10 µM) or vehicle control for 2 hr, then differentiated with PMA±MOTS-c (10 µM) for 2 hr, at which time RNA was collected for bulk RNA-seq analysis (n=6); false discovery rate (FDR)<5%. (**C**) Multidimensional scaling (MDS) analysis across control, PMA, and PMA+MOTS-c groups based on RNA-seq expression profiles after DESeq2 VST normalization. (**D**) Heatmap of significantly differentially regulated genes by MOTS-c by DESeq2 analysis. (**E**) Protein-protein interaction network analysis based on genes that were significantly differentially up- and downregulated by MOTS-c (FDR<5%) using the STRING (Search Tool for the Retrieval of Interacting Genes/Proteins) database version 11.0 (Szklarczyk et al., 2019). (**F**) Significantly enriched biological functions based on gene set enrichment analysis (GSEA) using Gene Ontology (GO). Selected groups are shown, full data in Supplementary file 1. (**G**) Correlation plot of gene expression changes by DESeq2 upon (**i**) *regular* induction of differentiation (control vs*.* PMA) compared to (ii) MOTS*-*c-directed induction of differentiation (PMA vs. PMA+MOTS-c). Spearman rank correlation (Rho) and significance of this correlation are reported. Genes that are significantly regulated only upon MOTS-c treatment but not during normal differentiation are highlighted in red and may underlie a specific MOTS-c-induced macrophage state. (**H**) Protein-protein interaction network analysis based on the 64 genes that were significantly differentially up- or downregulated by MOTS-c (FDR<5%) as described in (**G**) using the STRING database version 11.0 (Szklarczyk et al., 2019). MΦ=macrophage. Figure 4—source data 1.PDF of uncropped western blots with relevant bands indicated. Figure 4—source data 2.Original western blot TIF files from ChemiDoc.

Because HDPs can regulate monocyte activation and differentiation (Pena et al., 2013; Bowdish et al., 2004; Davidson et al., 2004; Cecotto et al., 2022), we next tested whether MOTS-c can modulate monocyte differentiation. We previously reported that MOTS-c translocates to the nucleus upon cellular stress to regulate a range of nuclear genes, indicating that mitochondrial-encoded factors can regulate the nuclear genome (Kim et al., 2018; Kang et al., 2021; Kong et al., 2021; Reynolds, 2019). Indeed, endogenous MOTS-c translocated to the nucleus upon differentiation of THP-1 monocytes by PMA in a time-dependent manner (Figure 4A) and following LPS+IFNγ stimulation (Figure 4—figure supplement 1A). Consistent with our previous reports (Lee et al., 2015; Kim et al., 2018; Kang et al., 2021; Kong et al., 2021; Reynolds et al., 2021), exogenously treated MOTS-c readily entered THP-1 monocytes (Figure 4—figure supplement 1B); the uptake was significantly retarded at a lower temperature (4°C) (Figure 4—figure supplement 1C), indicating the potential involvement of active transport (Drin et al., 2003). Exogenously treated MOTS-c peptide also showed strong nuclear localization (Figure 4B and Figure 4—figure supplement 1D). To test whether MOTS-c regulates nuclear transcriptional programming during early monocyte differentiation, we performed bulk RNA-seq on THP-1 monocytes that were treated with PMA or PMA+MOTS-c for 2 hr (Figure 4C–H). Multidimensional scaling (MDS) (Chen and Meltzer, 2005) revealed that the overall expression profiles of PMA and PMA+MOTS-c monocytes were distinct, as they were clearly separated in the MDS two-dimensional space (Figure 4C). Notably, the separation of the two groups occurred mostly on a single dimension (i.e*.* dimension 1, which captures the largest proportion of variance among samples), compatible with the notion that MOTS-c treatment led to acceleration in differentiation-related gene expression changes. MOTS-c differentially regulated 945 genes between PMA vs. PMA+MOTS-c groups (FDR<5%) (Figure 4D; Supplementary file 1), comparable to LL-37, which can affect the expression of >900 nuclear genes in human monocytes (Hancock et al., 2016).

**Figure 4—figure supplement 1.**
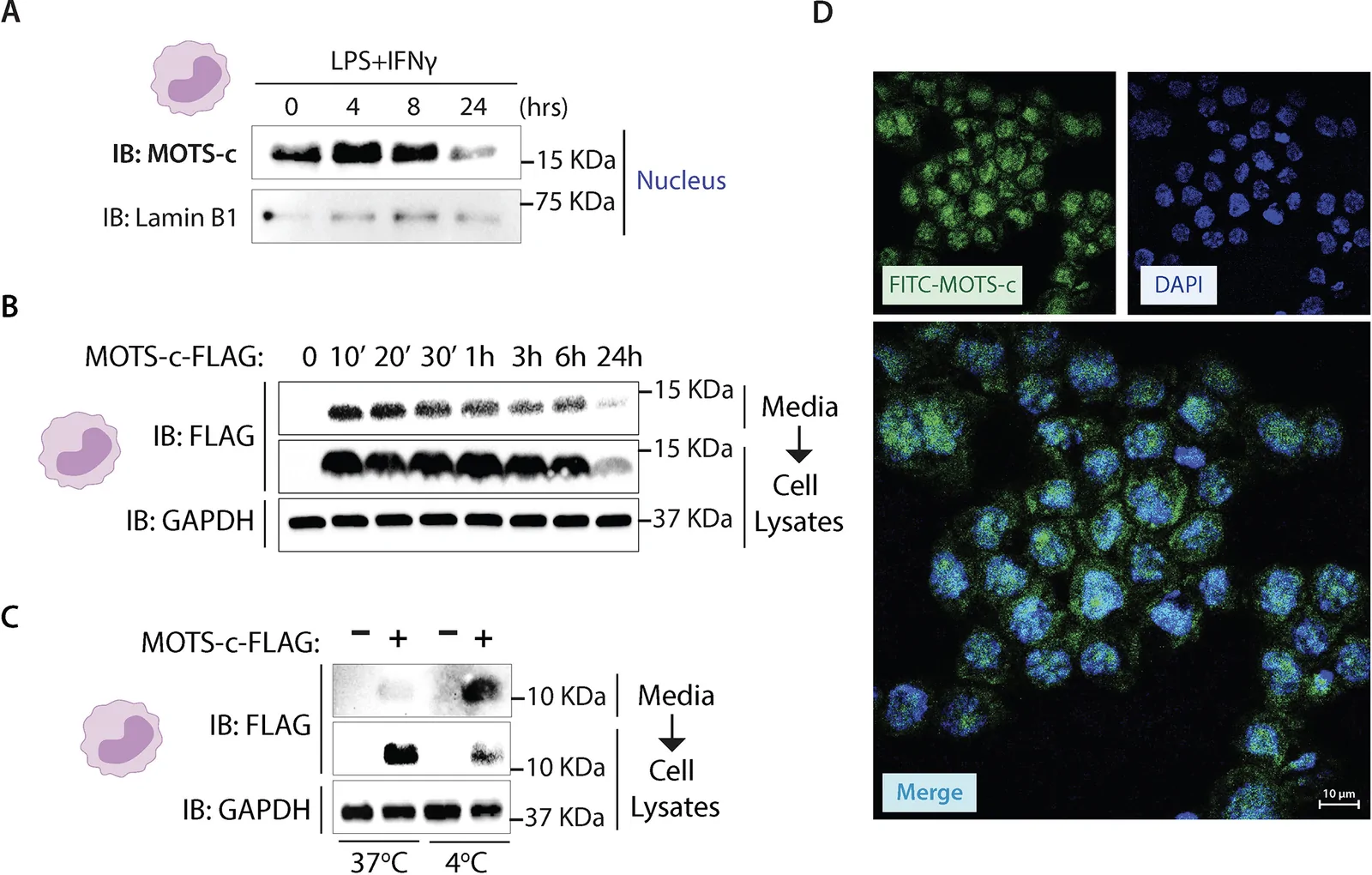
Nuclear MOTS-c in monocytes during activation and exogenous MOTS-c treatment. (**A**) MOTS-c translocates to the nucleus upon activation by lipopolysaccharides (LPS)+interferon gamma (IFNγ). A time-course measurement of endogenous MOTS-c in purified nuclear extracts following monocyte (THP-1) activation by LPS+IFNγ. Lamin B1 was used as a loading control for purified nuclear samples. See also Figure 4A. (**B**) Time-course detection of intracellular MOTS-c-FLAG peptide (10 µM) following treatment in THP-1 monocytes. (**C**) Intracellular levels of MOTS-c-FLAG peptide (10 µM) following treatment in THP-1 monocytes at different temperatures (i.e*.* 4°C vs*.* 37°C) for 30 min. (**D**) Exogenously treated MOTS-c peptide enters monocytes and localizes to the nucleus. Confocal fluorescence images of monocytes (THP-1) treated with FITC-MOTS-c (1 μM) for 30 min, showing nuclear localization. Nucleus marked by DAPI staining. Bar, 10 μm. Figure 4—figure supplement 1—source data 1.PDF of uncropped western blots with relevant bands indicated. Figure 4—figure supplement 1—source data 2.Original western blot TIF files from ChemiDoc.

Using the STRING (Search Tool for the Retrieval of Interacting Genes/Proteins) database version 11.0 (Szklarczyk et al., 2019), which can assess putative changes in protein-protein interaction networks based on our RNA-seq analysis, we identified large gene clusters in the PMA+MOTS-c group, compared to the PMA group, that were related to (i) ribosomes and translation initiation and (ii) chromatin dynamics (Figure 4E; full results in Supplementary file 2). Consistently, functional enrichment analysis for Gene Ontology (GO) gene sets revealed that the most significantly targeted functions by MOTS-c included (i) ribosomal and translational processes and (ii) chromatin dynamics (selected terms in Figure 4F; full results in Supplementary file 2). We then compared gene expression changes during normal differentiation (control vs*.* PMA) and MOTS-c-programmed differentiation (PMA vs. PMA+MOTS-c), and Spearman rank correlation (Rho) analysis revealed that changes were significantly correlated, consistent with the notion that MOTS-c treatment can accelerate the normal macrophage differentiation program (Figure 4G). In this context, we identified 64 genes that were specifically attributed to MOTS-c regulation after PMA induction (Figure 4G; Supplementary file 1D), of which ribosomal genes were again represented based on STRING analysis (Figure 4H). Consistently, ribosomal levels and differential translation are known to regulate cellular differentiation and lineage commitment (Khajuria et al., 2018; Buszczak et al., 2014; Ingolia et al., 2011; Shi and Barna, 2015), indicating that MOTS-c may broadly impact gene expression during monocyte differentiation.

### In vitro MOTS-c-treated human monocytes yield functionally distinct macrophages

**Figure 5.**
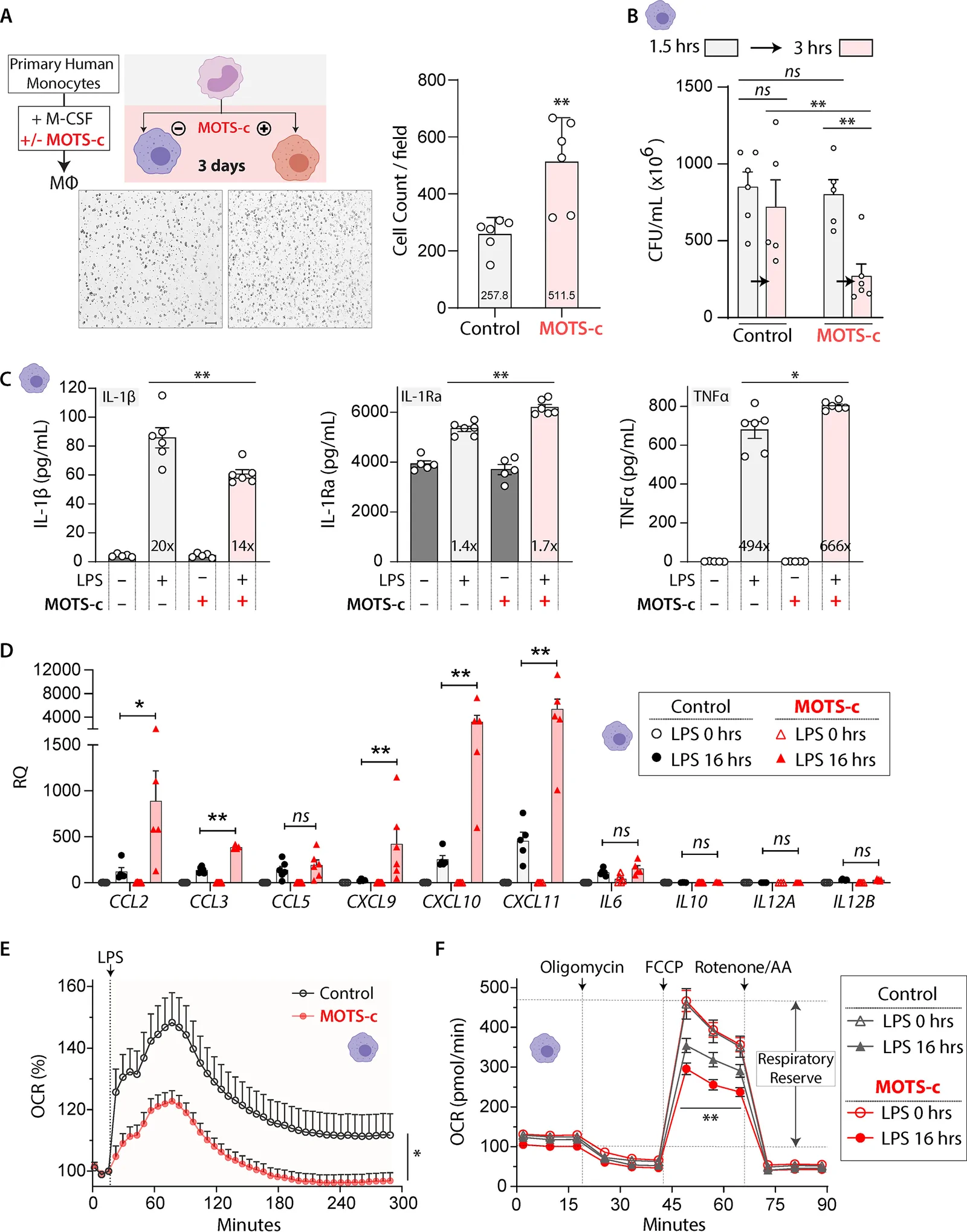
MOTS-c promotes the generation of macrophages with enhanced antibacterial capacity. (**A**) Primary human monocytes were differentiated by macrophage colony-stimulating factor (M-CSF) for 3 days with MOTS-c (10 µM) or vehicle (ddH_2_O) (2 hr priming, then single treatment with M-CSF). Representative images of adhered macrophages (n=6; MΦ=macrophage; bar, 10 µm). (**B–F**) THP-1 macrophages were differentiated for 4 days with/without MOTS-c treatment (10 µM; 2 hr priming, then single treatment with phorbol myristate acetate [PMA]). (**B**) Gentamicin protection assay in MOTS-c-programmed THP-1 macrophages following 1.5 or 3 hr post-infection of *E. coli* (MOI: 10). CFU: colony-forming units (n=6). (**C–F**) MOTS-c-programmed THP-1 macrophages were stimulated with lipopolysaccharides (LPS) (100 ng/ml) and (**C**) secreted levels of IL-1β, IL-1Ra, and TNF-α measured by ELISA after 20 hr (n=6), (**D**) cytokine expression levels determined by RT-qPCR after 16 hr (n=6), and (**E–F**) metabolic flux assessed (n=15) by cellular respiration (oxygen consumption rate [OCR]) (**E**) immediately after LPS stimulation and (**F**) 16 hr after LPS stimulation. Data are expressed as mean ± SEM. Mann-Whitney test, except for (**E, F**), which used two-way ANOVA repeated measures. *p<0.05, **p<0.01, ***p<0.001.

Due to the impact of MOTS-c on the transcriptome of THP-1 monocytes, we then asked whether MOTS-c can regulate monocyte differentiation to produce macrophages that are functionally distinct. A single exposure to MOTS-c during differentiation enhanced monocyte adherence, an important step for extravasation and differentiation to macrophages (Otto et al., 2021). We found that a greater number of MOTS-c-programmed primary human monocytes became adherent 3 days (Figure 5A) and 6 days (Figure 5—figure supplement 1A) after M-CSF stimulation, indicating an accelerated differentiation program, consistent with our RNA-seq analysis (Figure 4C and G). Next, we exposed THP-1 monocytes to MOTS-c at the onset of differentiation and functionally characterized the MOTS-c-programmed macrophages 4 days later. First, MOTS-c-programmed THP-1 macrophages exhibited a significant increase in bacterial killing capacity, determined using a gentamicin protection assay (Figure 5B). Second, we characterized the differential response of MOTS-c-programmed macrophages to LPS stimulation, including cytokine secretion/expression and cellular metabolism (Figure 5C–F). MOTS-c-programmed THP-1 macrophages exhibited (i) a shift in LPS-induced secretion of IL-1β, IL-1Ra, and TNF-α as measured by ELISA (Figure 5C) and (ii) selective impact on LPS-induced chemokine and cytokine gene expression as measured by RT-qPCR (Figure 5D and Figure 5—figure supplement 1B), and (iii) suppressed oxygen consumption in response to LPS, a metabolic reflection of macrophage activity (Viola et al., 2019; Russell et al., 2019; Langston et al., 2017; Odegaard and Chawla, 2011; Biswas and Mantovani, 2012), following acute stimulation (Figure 5E) and later reduced spare respiratory capacity 16 hr post-stimulation (Figure 5F). These results are in line with the previously described impact of LL-37, a well-described human HDP, in reprogramming monocytes to differentiate into macrophages with enhanced antibacterial capacity (van der Does et al., 2010), suggesting that the mitochondrial-encoded MOTS-c may act in a similar fashion.

**Figure 5—figure supplement 1.**
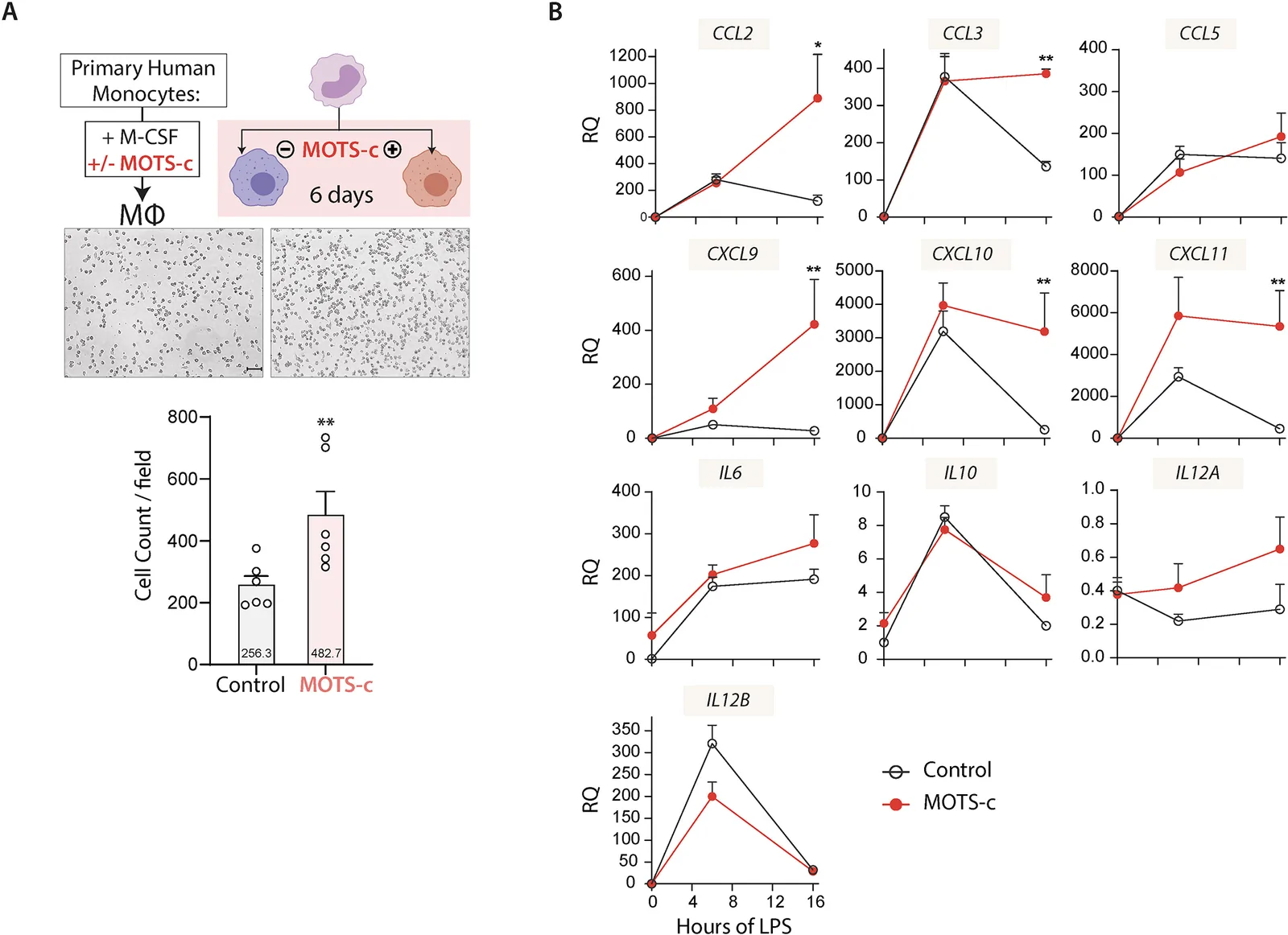
The presence of MOTS-c during monocyte-derived macrophage differentiation generates macrophages with an altered phenotype. (**A**) MOTS-c affects primary human monocyte differentiation. Primary human monocytes were differentiated with macrophage colony-stimulating factor (M-CSF) for 6 days with MOTS-c (10 μM) treatment or vehicle (ddH_2_O) only during the first 24 hr (n=6; MΦ=macrophage; bar, 10 μm). Adherent macrophages were imaged and counted. See also Figure 5A. (**B**) Macrophages (THP-1) were differentiated with MOTS-c (10 μM) or vehicle (ddH_2_O) (n=6) for 4 days. Time-course gene expression levels in MOTS-c-programmed macrophages by qPCR of cytokines *CCL2, CCL3, CCL5, CXCL9, CXCL10, CXCL11, IL6, IL10, IL12A*, and *IL12B* following lipopolysaccharides (LPS) treatment (100 ng/ml) (0, 6, and 16 hr). Relative gene expression, calculated based on the 0 time point, is shown. RQ: relative quantification. See also Figure 5D. Data expressed as mean ± SEM. Mann-Whitney test, *p<0.05, **p<0.01.

### Exposure of primary mouse bone marrow cells to MOTS-c promotes the emergence of a distinct subset of mature macrophages in both sexes throughout aging

**Figure 6.**
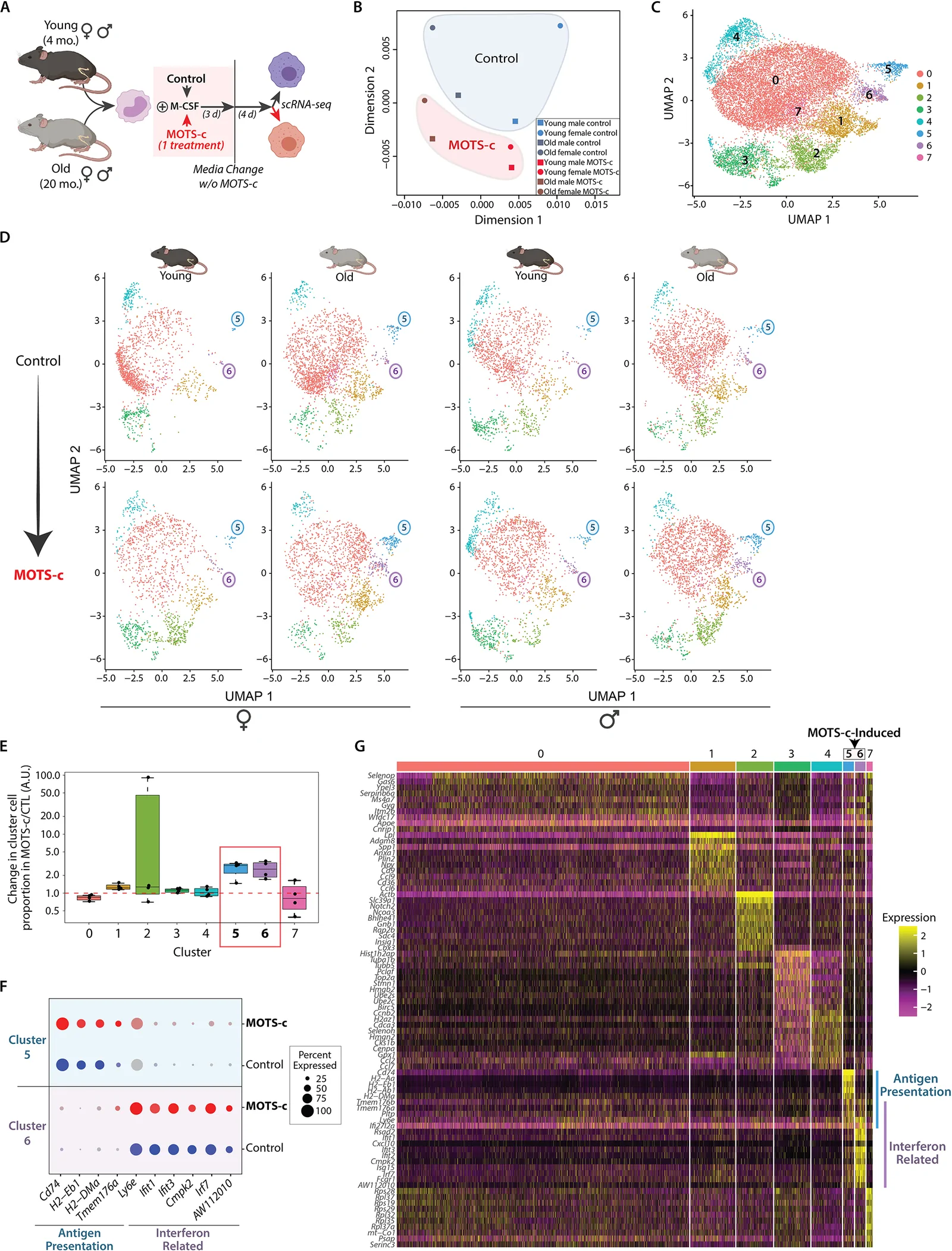
MOTS-c generates unique macrophages characterized by enhanced interferon signaling and antigen presentation in an age-related manner. (**A**) Single-cell RNA-seq (scRNA-seq) was performed on bone marrow-derived macrophages (BMDMs) from young (4 mo.) and old (20 mo.) mice of both sexes that were differentiated for 7 days in the presence of MOTS-c (10 µM) or vehicle (ddH_2_O), treated once concomitantly with first exposure to M-CSF, and present only for the first 3 days of differentiation. (**B**) Multidimensional scaling (MDS) analysis across each of the eight groups based on pseudobulk gene expression profiles for each biological group after performing DESeq2 VST normalization. (**C–D**) Uniform manifold approximation and projection (UMAP) plot on (**C**) all mice and (**D**) separated by age and sex, with cells color-coded based on shared nearest neighbor (SNN) clustering. Clusters 5 and 6 were enriched in MOTS-c-programmed BMDM populations. (**E**) Box plot of relative cluster cell proportion ratios between MOTS-c-programmed vs*.* control macrophages across clusters. Note that clusters 5 and 6 are consistently found in higher proportion in MOTS-c-treated samples compared to their corresponding control condition. (**F**) Dotplot of select genes enriched in clusters 5 and 6 (see also Figure 6—figure supplements 2–4 and Supplementary file 3). (**G**) Heatmap of the top 10 differential gene markers of each of the eight clusters in BMDMs induced in the presence/absence of MOTS-c (false discovery rate [FDR]<5%).

MOTS-c has a significant impact on aging physiology (Lee et al., 2015; Reynolds et al., 2021), in part, by acting as a regulator of adaptive stress responses (Lee et al., 2015; Kim et al., 2018; Kang et al., 2021; Kong et al., 2021; Reynolds et al., 2021; Mottis et al., 2019; Galluzzi et al., 2018) and is considered an emerging mitochondrial hallmark of aging (López-Otín et al., 2023; López-Otín et al., 2016). In addition, aging is also accompanied by maladaptive immune responses, including a shift in macrophage function (Franceschi et al., 2018; Pawelec, 2017; Pawelec, 2018; Panda et al., 2009; Goronzy and Weyand, 2013; Mahbub et al., 2012; Lloberas and Celada, 2002; Plowden et al., 2004; Fei et al., 2016; Minhas et al., 2019). Thus, we tested the impact of early MOTS-c exposure on the differentiation trajectories of primary progenitors from the bone marrow of female and male, young and old C57BL/6JNia mice (Figure 6A). Importantly, we used single-cell analyses to investigate heterogeneity in resulting primary macrophage populations (i.e*.* different macrophage ‘states’), reflecting their broad spectrum of transcriptional programs trained to dynamically adapt to their environment (Murray, 2017; Ginhoux et al., 2016; Xue et al., 2014).

**Figure 6—figure supplement 1.**
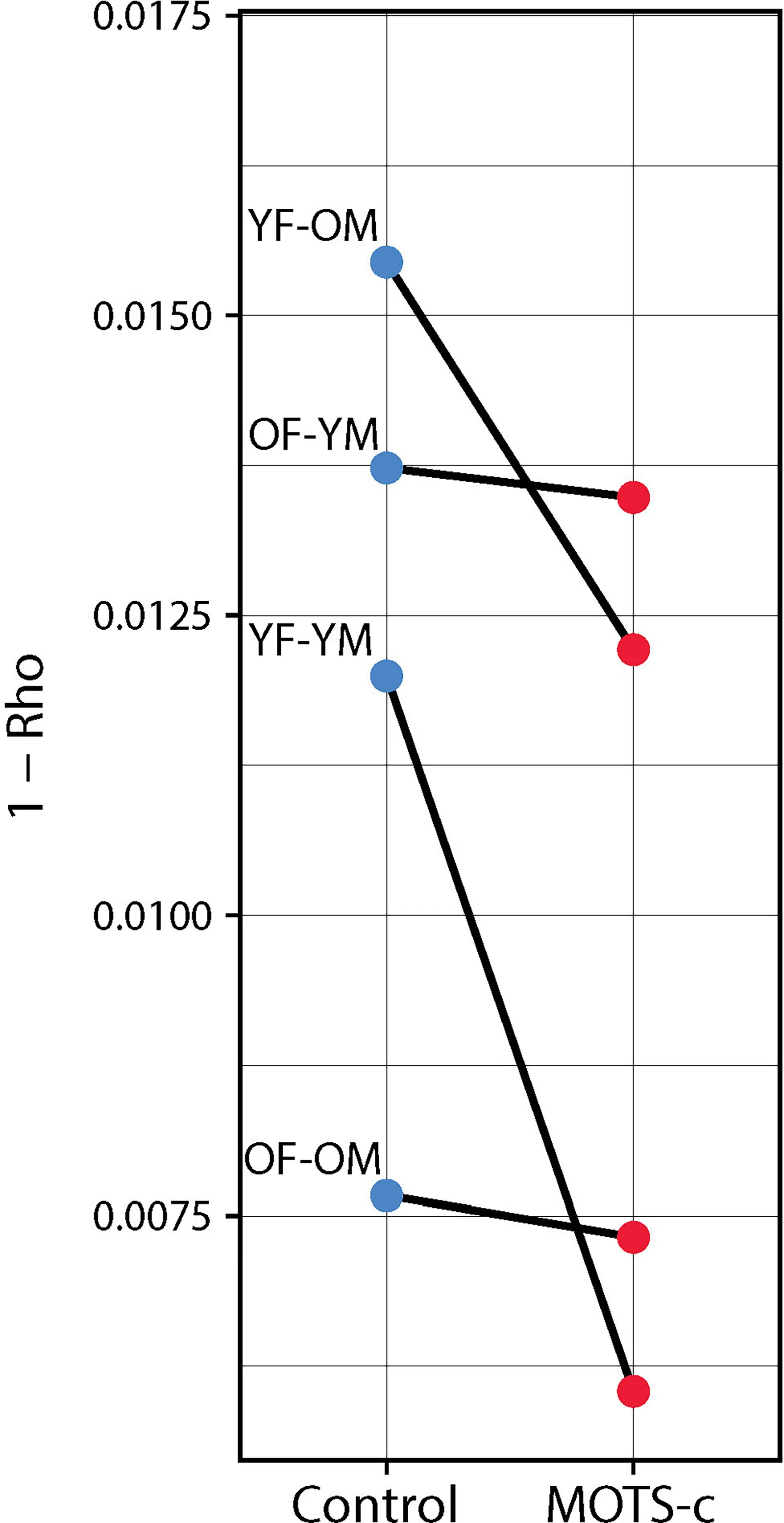
Pairwise distance analysis between female and male samples in control and MOTS-c-treated conditions for bone marrow-derived macrophage (BMDM) pseudobulk transcriptomes. The distance between each pair of (female, male) pseudobulk sample is computed as 1–Rho for each pair (YF-YM, YF-OM, OF-YM, and OF-OM) in the control and MOTS-c-treated conditions. To test whether the distance between (female, male) pseudobulk sample pair is reduced upon MOTS-c treatment, we used a one-sided paired Wilcoxon rank-sum test (p~0.0625). YF = young female; YM = young male; OF = old female; OM = old male.

**Figure 6—figure supplement 2.**
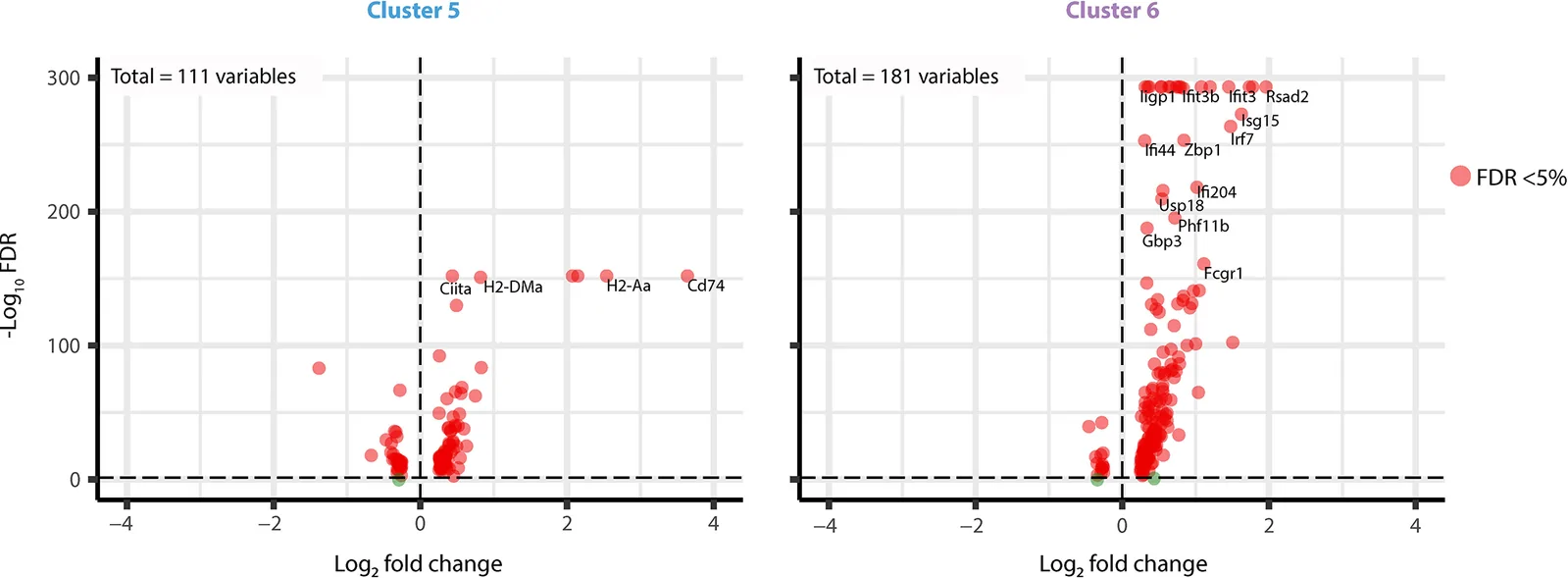
Genes enriched in MOTS-c-programmed macrophage populations. Single-cell RNA-seq (scRNA-seq) was performed on bone marrow-derived macrophages (BMDMs) from young (4 mo.) and old (20 mo.) mice of both sexes that were differentiated for 7 days with MOTS-c (10 μM) or vehicle (ddH_2_O). MOTS-c was given only once concomitantly with macrophage colony-stimulating factor (M-CSF) at the onset of differentiation, and the media was replaced after 3 days in both the control- and MOTS-c-treated conditions (see also Figure 6A). Volcano plots on differentially expressed genes in clusters 5 and 6 that are enriched by MOTS-c are shown here (see also Figure 6D–F, Figure 6—figure supplements 3 and 4, and Supplementary file 3).

**Figure 6—figure supplement 3.**
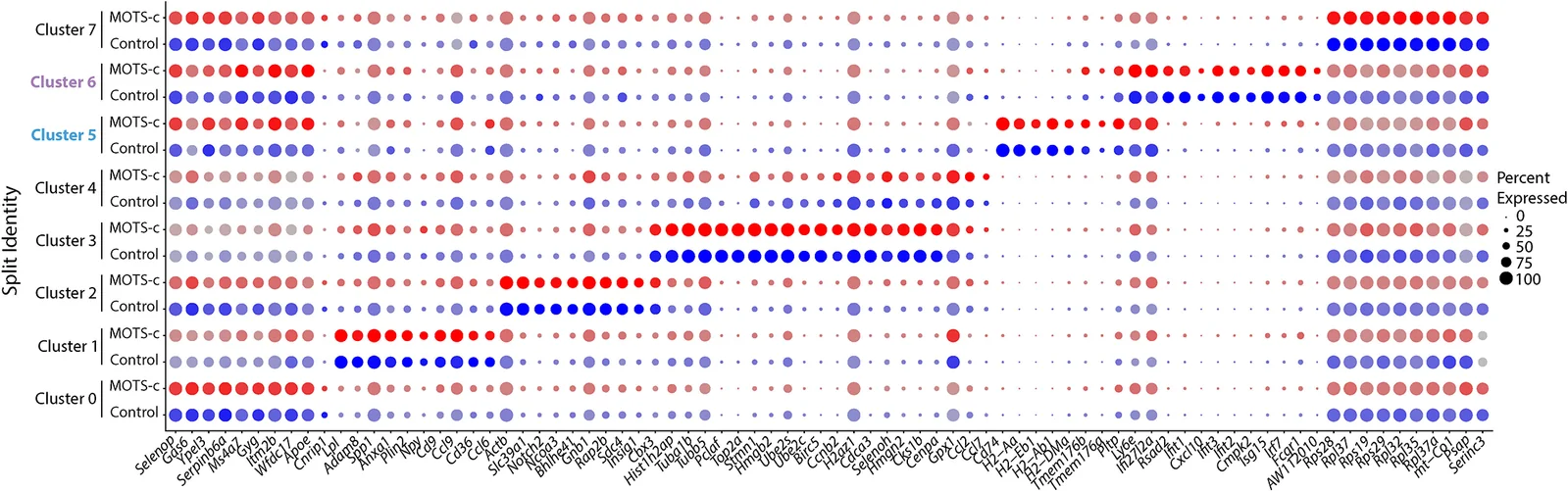
MOTS-c generates macrophages that uniquely express genes related to antigen presentation and interferon signaling. Single-cell RNA-seq (scRNA-seq) was performed on bone marrow-derived macrophages (BMDMs) from young (4 mo.) and old (20 mo.) mice of both sexes that were differentiated for 7 days with MOTS-c (10 μM) or vehicle (ddH_2_O). MOTS-c was given only once concomitantly with macrophage colony-stimulating factor (M-CSF) at the onset of differentiation, and the media was replaced after 3 days in both the control- and MOTS-c-treated conditions (see also Figure 6A). Dotplot of genes enriched in each of the eight clusters, showing MOTS-c-programmed BMDMs (clusters 5 and 6) have a unique gene expression profile not present in other clusters (see also Figure 6D–F, Figure 6—figure supplements 2 and 4, and Supplementary file 3).

**Figure 6—figure supplement 4.**
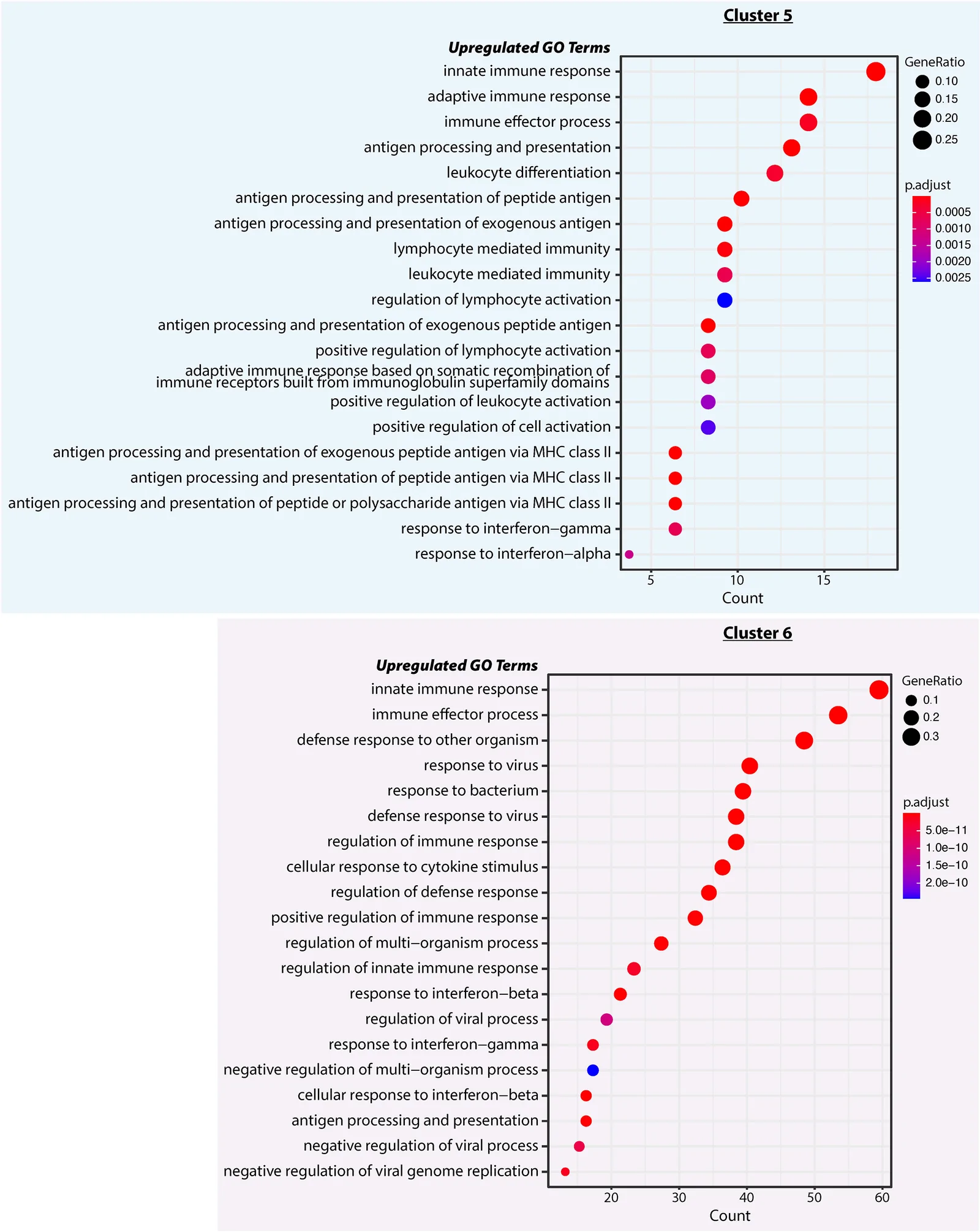
MOTS-c-programmed macrophage populations exhibit differential regulation of antigen presentation and interferon-related pathways. Single-cell RNA-seq (scRNA-seq) was performed on bone marrow-derived macrophages (BMDMs) from young (4 mo.) and old (20 mo.) mice of both sexes that were differentiated for 7 days with MOTS-c (10 μM) or vehicle (ddH_2_O). MOTS-c was given only once concomitantly with macrophage colony-stimulating factor (M-CSF) at the onset of differentiation, and the media was replaced after 3 days in both the control- and MOTS-c-treated conditions (see also Figure 6A). Dotplots of biological processes derived from scRNA-seq data for clusters 5 and 6, based on gene set enrichment analysis (GSEA) of Gene Ontology Biological Process (GO_BP) at false discovery rate (FDR)<5% (see also Figure 6D–F, Figure 6—figure supplements 2 and 3, and Supplementary file 3).

Specifically, we used single-cell RNA-seq (scRNA-seq) to determine whether early exposure (during the first 3 days of differentiation) of primary mouse bone marrow progenitors to MOTS-c may promote the emergence of transcriptionally distinct macrophage subsets. Because of the age-dependent shift in macrophage adaptive capacity (van Beek et al., 2019), we collected bone marrow cells from young (4 mo.) and old (20 mo.) mice of both sexes and differentiated them for 7 days±MOTS-c into bone marrow-derived macrophages (BMDMs). MOTS-c was given only once concomitantly with M-CSF at the onset of differentiation, and the media was replaced after 3 days in both the control- and MOTS-c-treated conditions (Figure 6A). scRNA-seq was performed at day 7 of differentiation, generating libraries for each biological group separately (N=5 animals per group, one library for each group obtained by equicellular mixing of BMDMs from each animal). To minimize the negative impact of batch effects, all samples were processed in parallel from bone marrow collection to library preparation and sequencing.

First, we asked whether there were global changes to mature BMDM transcriptomes upon only early exposure to MOTS-c, irrespective of age and sex. For this purpose, we decided to leverage a pseudobulk approach, which best controls false discovery rates (FDR) (Squair et al., 2021). After aggregating reads for each independent library, we normalized the data using the Variance Stabilizing Transformation from the ‘DESeq2’ R package. MDS revealed that macrophages were clearly separated by age on dimension 1 and by MOTS-c treatment on dimension 2 (Figure 6B). Intriguingly, female BMDMs showed a greater shift in response to MOTS-c treatment than male BMDMs; notably, female MOTS-c-treated BMDMs appeared ‘masculinized’ (i.e*.* closer to male samples in MDS space, and with reduced sample-to-sample distance; Figure 6B and Figure 6—figure supplement 1). With limited sample number, future work will be needed to elucidate interactions of MOTS-c treatment with age and sex in the context of BMDM transcriptional programming. However, our pseudobulk analysis reveals global remodeling of BMDM programs upon transient early exposure to MOTS-c.

Using a shared nearest neighbor modularity optimization with the ‘Seurat’ R package, we identified eight BMDM clusters, indicative of different latent transcriptional states, consistent with a heterogeneous mature macrophage population. Importantly, the eight distinct single-cell BMDM clusters were comprised of cells from young and old mice of both sexes (cluster labeling is shown on our data using a nonlinear dimensionality-reduction technique, UMAP [uniform manifold approximation and projection]) (Figure 6C). Intriguingly, two clusters (clusters 5 and 6) were consistently enriched in MOTS-c-treated condition, and their increase was more pronounced in BMDMs derived from older animals regardless of sex (Figure 6D and E; Supplementary file 3). To note, aging alone increased the proportion of clusters 5 and 6 (Figure 6D, upper panels), consistent with the connection between MOTS-c signaling and aging (Lee et al., 2015; Reynolds et al., 2021; Fuku et al., 2015; D’Souza et al., 2020; Zempo et al., 2021). Cluster 5 was largely enriched in the expression of genes relevant to antigen presentation, whereas cluster 6 showed increased expression of interferon-related genes (Figure 6F–G, Figure 6—figure supplements 2 and 3; full analysis in Supplementary file 3); some genes were shared between these categories. Consistently, overrepresentation analysis using Gene Ontology Biological Process terms showed a clear signature of antigen presentation processes for cluster 5 and IFN-related processes for cluster 6 (FDR5%; top 20 pathways in Figure 6—figure supplement 4; full analysis in Supplementary file 4). Notably, interferon signaling can be triggered not only by mtDNA (West and Shadel, 2017), but can also induce the expression mtDNA-encoded MOTS-c (Figure 3C and E; Tsuzuki et al., 1983). Increased expression of genes involved in antigen presentation and interferon signaling with age has been observed in monocytes and macrophages in mice (Barman et al., 2022; Almanzar et al., 2020; Teo et al., 2023; Kimmel et al., 2019; Benayoun et al., 2019). In fact, the antigen presentation genes *H2-Aa, H2-Ab1, H2-Eb1, Cd74,* and *AW112010* and interferon-related genes *Irf7, Ifit2, Ifit3, Ifitm3*, and *Ifi204* are consistently upregulated with age in our data and that of others (Supplementary file 3; Barman et al., 2022; Almanzar et al., 2020; Teo et al., 2023; Kimmel et al., 2019), indicating a conserved effect of aging on monocytes/macrophages.

## Discussion

Here, we identify MOTS-c as a first-in-class mitochondrial-encoded HDP that can target bacteria and modulate monocyte differentiation. MOTS-c influenced nuclear gene expression during the early phase of monocyte-to-macrophage differentiation to generate distinct macrophage populations that are adapted to bacterial clearance. Our current working model is that MOTS-c initially mounts a preemptive attack on bacteria by aggregating them and preventing growth, then influences monocyte differentiation for efficient microbial clearance. Notably, HDPs possess anti-inflammatory effects upon clearing pathogens to promote a non-inflammatory post-infection resolution and alleviate over-responsive inflammation (e.g*.* sepsis) (Sun and Shang, 2015; Tyers and Wright, 2019), consistent with the systemic anti-inflammatory effects of MOTS-c (Lee et al., 2015; Kim et al., 2018; Kong et al., 2021; Reynolds, 2019; Ming et al., 2016; Hu and Chen, 2018; Che et al., 2019; Kim et al., 2019; Li et al., 2019; Liu et al., 2019; Lu et al., 2019a; Lu et al., 2019b; Weng et al., 2019; Yan et al., 2019; Wei et al., 2020; Xinqiang et al., 2020; Yin et al., 2020).

Our initial hypothesis was that interbacterial communication using peptides may still exist between bacteria-derived mitochondria and bacteria. Endosymbiosis is a form of sustained infection whereby the primal bacteria and our ancestral cell communicated to coordinate the establishment of mitochondria and eukaryotic life. Such communication was likely derived from immunological processes to regulate each other, which may have laid the foundation for cellular signal transduction. HDPs often regulate highly conserved cellular functions, such as ribosomes and protein metabolism (Le et al., 2017), consistent with the stress-responsive regulatory functions of MOTS-c in mammalian cells (Mottis et al., 2019; Galluzzi et al., 2018; Quirós et al., 2016; Sloan et al., 2018; Reynolds et al., 2020; Tan and Finkel, 2020; Gubert and Hannan, 2021; Figure 4E–H). Evidence for MOTS-c as a therapeutic agent against MRSA in mice adds translational potential for mtDNA-encoded immune peptides for combating bacterial infection (Zhai et al., 2017). Further, it is likely that MOTS-c, consistent with other HDPs, may also target viruses. Human myeloblast cells that were infected with Sendai virus to induce IFN expression showed considerable induction of transcripts from the mitochondrial rRNA loci (~75% of induced transcripts) (Tsuzuki et al., 1983), a study that influenced the discovery of MOTS-c (Lee et al., 2015). Consistently, we found that MOTS-c is endogenously expressed in monocytes and that it can be induced by IFNγ stimulation (Figure 3E).

Our data supports an immunological role for mitochondrial-encoded MOTS-c and provides a proof of principle for mitochondrial-encoded HDPs. Together with the ever-increasing identification of sORFs in both our mitonuclear genomes, multiple unannotated HDPs may exist in humans with the potential for clinical use against a broad range of infections (Gomes et al., 2018; Mishra et al., 2017).

## Methods

### Bacterial strains

*E. coli* (BL21 [DE3]; NEB), MRSA (ATCC 33592), *S. typhimurium* (ATCC 14028), and *P. aeruginosa* (ATCC 27853) were used. *E. coli* cells were transformed to conditionally overexpress MOTS-c in pSF-T7/LacO (Sigma, OGS500) using IPTG. Bacteria were routinely maintained in LB medium (liquid and agar). A modified M9 minimal medium, composed of 0.1× of M9 Minimal Salts without NaCl (BD Difco), 1 mM MgSO_4_, 1% glucose, and 2.5 g/l peptone, was used in select studies.

### Phase-contrast light microscopy

Bacteria were grown overnight in LB broth, from which a 1:100 inoculation was made in LB broth and grown to mid-log phase (~0.6 OD_600_), measured using a SpectraMax M3 Spectrophotometer (Molecular Devices), in a 37°C shaker at 225 rpm. 1 ml of mid-log *E. coli* (BL21) and 3 ml of mid-log MRSA were collected and treated in modified M9 minimal medium to achieve macroscopic bacterial aggregates. Approximately 3 µl of aggregates were transferred to Superfrost Plus Micro Slides (VWR) with platinum-grade coverslips and imaged with phase-contrast mode using an EVOS FL Cell Imaging System.

### Scanning electron microscopy

Samples were fixed overnight at 4°C in 3% glutaraldehyde. Fixed cells were placed onto 0.2 μm Nuclepore Track-Etch Membrane filters (Whatman) and allowed to air-dry for 15 min prior to ethanol dehydration. The dehydration series progressed from an initial wash concentration of 30% ethanol with 30 min stepwise increments to a final wash of 100% ethanol prior to critical point drying (Autosamdri-815, Toursimis). Samples were then sputter-coated (Cressington) with approximately 3 nm of Pd. Electron micrographs were obtained with a JEOL-7001 FEG Scanning Electron Microscope.

### Confocal imaging

Cellular images were obtained using a Zeiss LSM700 confocal microscope system (Germany). THP-1 monocytes were treated with synthetic MOTS-c peptide tagged with an FITC fluorophore (1 μM) for 30 min. After washing three times with PBS, cells were fixed using 4% paraformaldehyde and permeabilized in 0.2% Triton X-100. Fixed cells were incubated with DAPI (Sigma) for 30 min, followed by three additional washes with 0.1% PBST. The cells were spread onto glass coverslips and attached to MicroSlides (cat#48311-703, VWR) using ProLong Gold antifade mountant (cat#P36934, Thermo Fisher).

### Bacterial growth measurements

Overnight bacterial cultures were diluted 1:1000 and grown in modified M9 minimal medium (0.1× M9, 5% glucose, 1 mM MgSO_4_, 1.0 g/l peptone) with MOTS-c (or vehicle) in a 37°C shaker at 225 rpm. Bacterial density was measured at OD_600_ every hour in two-sided polystyrene cuvettes (VWR) using a SpectraMax M3 Spectrophotometer (Molecular Devices). For plate assays, modified M9 minimal medium agar (1.5%) plates (35 mm Petri dishes) were made.

### ATP assay

BacTiter-Glo Microbial Cell Viability Kit (Promega) was used to assess bacterial cell viability and metabolism by measuring luminescence correlated with the amount of intracellular ATP. Bacteria were grown overnight in LB broth, from which a 1:100 inoculation was grown in LB broth to reach mid-log phase (~0.6 OD_600_) in a 37°C shaker at 225 rpm. 1 ml of log-phase bacteria was collected, resuspended in modified M9 minimal medium, treated with MOTS-c (100 µM), then subjected to luciferase reaction per the manufacturer’s instructions. Luminescence was measured using SpectraMax M3 Spectrophotometer (Molecular Devices) and values reported as RLU (relative light units).

### SYTOX Green assay

SYTOX Green Nucleic Acid Stain 5 mM in DMSO (Thermo Fisher) was used to assess bacterial membrane integrity. SYTOX Green does not cross intact membranes, but easily penetrates compromised membranes and stains nucleic acids (Roth et al., 1997). Fluorescence readings (bottom-read, excitation/emission at 504/523 nm) were recorded at various time points with one measurement taken before treatment (blank).

### Western blot

Whole cell and nuclear compartment were lysed with 8 M urea buffer containing a protease/phosphatase inhibitor cocktail (Thermo Fisher Scientific) and were sonicated for 15 s at 60% amplitude. The lysates were separated by 8–16% pre-cast SDS-PAGE gels (Bio-Rad) and transferred onto PVDF membranes. The membranes were blocked with 5% bovine serum albumin (BSA) in Tris-buffered saline with 0.05% Tween-20 and probed with primary antibody at 4°C overnight. The following antibodies were used: β-actin (Cell Signaling #8457), β-tubulin (Cell Signaling #2146), Lamin B1 (Cell Signaling #12586), GAPDH (Cell Signaling #5174), and FLAG (Millipore Sigma #F3165). For MOTS-c, a custom antibody was made from YenZym (no commercial RRID available). Proteins of interest were detected with anti-rabbit IgG HRP-linked antibody (Cell Signaling) and developed by Clarity Western ECL substrates (Bio-Rad). The membranes were imaged using the ChemiDoc XRS+ system (Bio-Rad).

### Human cell culture

THP-1 cells (RRID:CVCL_0006) were purchased, authenticated by STR profiling, and tested for mycoplasma by ATCC. They were routinely cultured at a range of cell density of 2×10^5^ cells/ml to 8×10^5^ cells/ml in RPMI 1640 (Corning) supplemented with 10% heat-inactivated fetal bovine serum (Omega Scientific) and 0.05 mM 2-mercaptoethanol at 37°C with 5% CO_2_. Macrophages were generated by culturing THP-1 (6×10^5^ /ml) cells with 15 nM PMA (Sigma-Aldrich) in the media. Human primary monocytes were isolated from leukocyte cones of healthy blood donors, obtained from USC/CHLA. Mononuclear cells were separated by centrifugation at 400×*g* for 20 min with underlying Histopaque-1077. The opaque interface between plasma and the Histopaque-1077 was collected for further purification by Red Blood Cell lysis buffer (Miltenyi). Monocytes were isolated by negative selection using the StraightFrom LRSC CD14 MicroBead Kit (cat# 130-117-026, Miltenyi). Purified human monocytes were seeded at a density of 1×10^6^ cells/ml in RPMI 1640 supplemented with 10% heat-inactivated fetal bovine serum and 100 ng/ml human M-CSF (Miltenyi).

### Human cell treatment

THP-1 cells were seeded at a density of 4×10^5^ cells/ml and reached 6×10^5^ cells/ml prior to treatment. PMA (Sigma-Aldrich) was reconstituted in DMSO and used at 15 nM. LPS from *E. coli* O111:B4 (List Biology Laboratories, Inc) was reconstituted in sterile water and used at 100 ng/ml. Human IFNγ (Sigma) was reconstituted in sterile water and used at 20 ng/ml. Synthetic MOTS-c peptide with a purity >95% by mass spectrometry (New England Peptides, now Biosynth) was reconstituted in sterile water and used at 10 µM.

### Nuclear fractionation

Nuclear fraction was isolated as previously described (Kim et al., 2018; Gagnon et al., 2014). Cells were harvested by centrifugation. The pellet was resuspended in hypotonic fractionation buffer (10 mM Tris, pH 7.5, 10 mM NaCl, 3 mM MgCl_2_, 0.3% NP-40, 10% glycerol, and EDTA-free protease inhibitor) and incubated on ice for 30 min and passed through a 31G needle five times and centrifuged. The nuclear pellet was washed with hypertonic fractionation buffer three times and resuspended in 8 M urea lysis buffer (1 M Tris, pH = 8).

### Gentamicin protection assay

THP-1 monocytes were seeded on a six-well plate at a concentration of 1.5×10^6^ cells/well and differentiated by PMA (15 nM) for 96 hr. For MOTS-c treatment, monocytes were first primed with MOTS-c (10 µM) for 2 hr, then treated only once with a mixture of PMA (15 nM) and MOTS-c (10 µM). *E. coli* (BL21) were added to differentiated THP-1 cells (MOI 10) and co-cultured for 1–2.5 hr, at which time gentamicin (50 µg/ml) (Fisher Scientific) was added. 30 min later, cells were washed with PBS and collected (total of 1.5 or 3 hr). Cells were lysed with 1% Triton X-100 and spread on LB agar plate. Bacterial colonies were counted after overnight incubation at 37°C.

### ELISA

THP-1 monocytes (n=6) were cultured in six-well plates at a density of 5×10^6^ cells/ml in 2 ml of RPMI 1640 (cat#45000-396, VWR, USA) supplemented with 10% fetal bovine serum (Omega Scientific) and incubated at 37°C in humidified air with 5% CO_2_. THP-1 cells were primed with MOTS-c peptide (10 μM) for 2 hr, then treated with a combination of PMA (15 nM)+MOTS c (10 μM). 96 hr post-differentiation, cells were treated with LPS (100 ng/ml) or vehicle for an additional 20 hr and supernatants were collected for cytokine analysis. The production levels of cytokines, including IL-1β (cat#BMS224-2), IL-1Ra (cat#BMS2080), and TNF-α (cat#BMS223-4), were measured in duplicate using human ELISA kits following the manufacturer’s instructions (Thermo Fisher) and SpectraMax M3 (Molecular Devices).

### Real-time qPCR

Total RNA was extracted and purified using the Direct-zol RNA MiniPrep kit (Zymo Research) following the manufacturer’s instructions. The total RNA (1.2 μg) was used to synthesize single-stranded cDNA using iScript cDNA synthesis Kit (Bio-Rad) and T100 Thermal Cycler (Bio-Rad) according to the manufacturer’s instructions. The relative mRNA levels of *CCL2, CCL3, CCL5, CXCL9, CXCL10, CXCL11, IL6, IL10, IL12A,* and *IL12B* were determined by the real-time quantitative PCR analysis using the SYBR Green Supermix (#1725275, Bio-Rad) and CFX Connect Real-time PCR Detection System (Bio-Rad). All reactions were performed in a total of 20 μl reaction volume containing 2 μl of diluted cDNA, 10 μl of SYBR Supermix, 1 μl of 10 μM forward primer, 1 μl of 10 μM reverse primer, and 6 μl of autoclaved distilled water. All samples were analyzed in duplicate with an endogenous control gene (β-actin) being analyzed at the same time. The relative expression of each gene was calculated from 2^-ΔΔCT^.

### Primer sequences used in real-time RT-qPCR

**Table:** 

Gene	Forward primer	Reverse primer
*IL12A*	GATGGCCCTGTGCCTTAGTA	TCAAGGGAGGATTTTTGTGG
*IL12B*	GGACATCATCAAACCTGACC	AGGGAGAAGTAGGAATGTGG
*IL10*	GTGATGCCCCAAGCTGAGA	CACGGCCTTGCTCTTGTTTT
*IL6*	TGCGTCCGTAGTTTCCTTCT	GCCTCAGACATCTCCAGTCC
*CCL2*	ATCAATGCCCCAGTCACC	AGTCTTCGGAGTTTGGG
*CCL3*	CGGTGTCATCTTCCTAACCA	GACATATTTCTGGACCCACTC
*CCL5*	TACCATGAAGGTCTCCGC	GACAAAGACGACTGCTGG
*CXCL9*	GAGTGCAAGGAACCCCAGTAGT	TTGTAGGTGGATAGTCCCTTGGTT
*CXCL10*	TTCAAGGAGTACCTCTCTCTAG	CTGGATTCAGACATCTCTTCTC
*CXCL11*	CCTGGGGTAAAAGCAGTGAA	TGGGATTTAGGCATCGTTGT

### Metabolic flux measurements

Real-time analysis of oxygen consumption rates (OCR) were measured in THP-1 cells using XF24/96 Extracellular Flux Analyzer (Seahorse Bioscience). Cells were seeded in Seahorse XF96 cell culture microplate (#101085-004, Seahorse Bioscience) at a density of 5×10^6^ cells/ml in 100 μl of RPMI 1640 (#45000-396, VWR) supplemented with 10% fetal bovine serum (Omega Scientific). The cells were treated without or with 10 μM MOTS-c for 2 hr, followed by treatment with PMA for 96 hr. LPS (100 ng/ml) was added either during OCR measurements (dispensed by XF96) or added for 16 hr prior to OCR measurements, and medium was replaced with XF assay buffer supplemented with 1 mM pyruvate and 12 mM D-glucose. ATP turnover, maximum respiratory capacity, and non-mitochondrial respiration were estimated by sequential addition of oligomycin (0.9 μM), carbonyl cyanide 4-[trifluoromethoxy]phenylhydrazone (FCCP, 1.0 μM), rotenone (0.5 μM), and antimycin A (0.5 μM). All readings were normalized to relative protein concentration.

### Bulk RNA-seq library preparation

1 μg of total RNA was subjected to rRNA depletion using the NEBNext rRNA Depletion Kit (New England Biolabs), according to the manufacturer’s protocol. Strand-specific RNA-seq libraries were then constructed using the SMARTer Stranded RNA-Seq Kit (Clontech #634839), according to the manufacturer’s protocol. Based on rRNA-depleted input amount, 13–15 cycles of amplification were performed to generate final RNA-seq libraries. Pooled libraries were sent for paired-end sequencing on the Illumina HiSeq-Xten platform at the Novogene Corporation (USA). The raw sequencing data has been deposited to the NCBI Sequence Read Archive (accession number PRJNA623667). The resulting data was analyzed with a standard RNA-seq data analysis pipeline (described below).

### Bulk RNA-seq analysis pipeline

To avoid the mapping issues due to overlapping sequence segments in paired-end reads, reads were hard-trimmed to 75 bp using Fastx toolkit v0.0.13. Reads were then further quality-trimmed using Trimgalore 0.4.4 to retain high-quality bases with Phred score>20. All reads were also trimmed by 6 bp from their 5' end to avoid poor qualities or random-hexamer-driven sequence biases. cDNA sequences of protein-coding and lncRNA genes were obtained through ENSEMBL Biomart for the GRCh38 build of the human genome (release v96). Trimmed reads were mapped to this reference using kallisto v0.43.0 and the –fr-stranded option (Bray et al., 2016). All bulk RNA-seq analyses were performed in the R statistical software version 3.4.1 (https://cran.r-project.org/). Read counts were imported into R and summarized at the gene level to estimate gene expression levels. We estimated differential gene expression between control, PMA, and PMA+MOTS-c-treated THP-1 RNA-seq samples using the ‘DESeq2’ R package (DESeq2 1.16.1) (Love et al., 2014). The heatmap of expression across samples for significant genes (Figure 5D) was plotted using the R package ‘pheatmap’ 1.0.10 (Kolde, 2015).

### Functional enrichment analysis

To perform functional enrichment analysis, we used the gene set enrichment analysis (GSEA) paradigm through R packages ‘phenoTest’ 1.24.0 and ‘qusage’ 2.10.0. GO gene sets were obtained from the Molecular Signature Database, C5 collection (c5.all.v6.2.symbols.gmt) (Subramanian et al., 2005). An FDR threshold of 0.05 was considered statistically significant. The –log_10_(FDR) value for GSEA enrichment is reported in Figure 3F as a barplot for selected terms, and FDR values of 0 were replaced by a small value of 10^–30^ to enable plotting on a reasonable scale. The full list of enriched GO terms at FDR 0.05 is reported in Supplementary file 2.

### STRING analysis

The list of significantly regulated genes in the PMA vs. PMA+MOTS-c conditions at FDR<0.05 was used to infer potential disruption to protein interaction networks using the STRING (Search Tool for the Retrieval of Interacting Genes/Proteins) tool database version 11.0 (Szklarczyk et al., 2019).

### Mouse husbandry

All animals were treated and housed in accordance with the Guide for Care and Use of Laboratory Animals. All experimental procedures were approved by the University of Southern California’s Institutional Animal Care and Use Committee (IACUC) under protocol #21275 and are in accordance with institutional and national guidelines. For murine peritonitis experiments, male and female C57BL/6J mice were obtained from Jackson Laboratory, and experiments were performed at 4–6 months of age. For scRNA-seq analyses, male and female C57BL/6JNia mice (4- and 20-month-old animals) were obtained from the National Institute on Aging (NIA) colony at Charles Rivers. Animals were acclimated at the SPF animal facility at USC for 2–4 weeks before any processing and were euthanized between 8 and 11 am to minimize circadian effects. In all cases, animals were euthanized using a ‘snaking order’ across all groups to minimize batch-processing confounds. All animals were euthanized by CO_2_ asphyxiation followed by cervical dislocation.

### Murine model of peritonitis

MRSA (ATCC 33592) were grown overnight in LB broth, from which a 1:100 inoculation was made in LB broth and grown to mid-log phase (~0.6 OD_600_), measured using a SpectraMax M3 Spectrophotometer (Molecular Devices), in a 37°C shaker at 225 rpm. 6×10^8^ or 4×10^8^ CFU of MRSA was washed and resuspended in 100 μM MOTS-c or water immediately prior to intraperitoneal injection with a 28G syringe. MRSA was serially diluted, plated on LB agar, and colonies counted after overnight incubation to confirm CFU inoculated. Mice were monitored for 72 hr and weighed daily. Moribund animals were euthanized by CO_2_ asphyxiation followed by cervical dislocation according to IACUC approved experimental guidelines. To heat-kill MRSA, MRSA resuspended in vehicle (water) was heated in a 70°C water bath for 20 min. Mice were euthanized either 6 hr or 72 hr post-inoculation, and blood was collected by cardiac puncture. Plasma was collected and stored at –80°C after centrifugation at 800×*g* for 10 min at 4°C. Cytokines and organ injury markers were measured in plasma following the manufacturer’s instructions using the Meso Scale Discovery V-PLEX Proinflammatory Panel 1 Mouse Kit (K15048D-1) for cytokines, Mouse ALT ELISA Kit (ab282882), Mouse AST ELISA Kit (ab263882), and Urea Assay Kit (ab83362).

### Derivation of BMDMs and MOTS-c treatment

We isolated BMDMs as previously described (Lu et al., 2020). Briefly, the long bones of each mouse were harvested and kept on ice in D-PBS (Corning) supplemented with 1% Penicillin/Streptomycin (Corning) until further processing. Muscle tissue was removed from the bones, and the bone marrow from cleaned bones was collected into clean tubes (Amend et al., 2016). Red blood cells from the marrow were removed using Red Blood Cell Lysis buffer (Miltenyi Biotech #130-094-183), according to the manufacturer’s instructions, albeit with no vortexing step and incubation for only 2 min. The suspension was filtered on 70 μm mesh filters to retain only single cells. Cells were plated in macrophage growth medium (DMEM/F12 [Corning], 10% FBS [Sigma], 1% Penicillin/Streptomycin [Corning], 2 ng/ml recombinant M-CSF [Miltenyi Biotech], and 10% L929-conditioned medium as an additional source of M-CSF). For cells undergoing MOTS-c treatment, this initial culture medium was supplemented with 10 μM MOTS-c peptide (New England Peptides, >95% purity). After 3 days, adherent cells were rinsed with D-PBS, and fresh macrophage growth media was added. Cells were collected after 7 days in culture, when BMDMs have completed differentiation. We differentiated BMDMs from five animals from each group (young females, young males, old females, old males, control, and MOTS-c treated; eight groups total).

### BMDM scRNA-seq library preparation

scRNA-seq libraries were prepared using Single Cell 3′ v2 Reagent Kits, according to the manufacturer’s instructions (10x Genomics). For scRNA-seq profiling, BMDMs were detached using ice-cold 10 mM EDTA, counted using a COUNTESS cell counter (Thermo Fisher Scientific), and samples from each sample group were pooled in an equicellular mix (8 mixes, 1 mix per biological condition). Using the 10x Genomics Single Cell 3′ v2 manufacturer’s instructions (10x Genomics), we loaded the microfluidics device with a targeted capture in each sample of 3000 cells. The eight samples were run in parallel on the same microfluidics chip and processed on a Chromium Controller instrument (10x Genomics), to generate single-cell Gel bead-in-EMulsions (GEMs). GEM-RT was performed in a C1000 Touch Thermal Cycler with a deep well module (Bio-Rad). The cDNA was amplified and cleaned up with SPRIselect Reagent Kit (Beckman Coulter Genomics). Single-indexed sequencing libraries were constructed using Chromium Single-Cell 3′ Library Kit. Library quality and quantification prior to pooling were assessed using a D1000 ScreenTape device on the TapeStation apparatus (Agilent). All barcoded libraries were combined in a single pool for sequencing, and sent for sequencing on three lanes of Hiseq-X-Ten at Novogene Corporation as paired-end 150 bp reads. The final average sequencing depth per cell was ~70,000 reads per cell.

### BMDM scRNA-seq data analysis

Reads were hard-trimmed to yield the lengths expected by the CellRanger pipeline (Read 1: 26 bp, Read 2: 98 bp) using the fastx_trimmer tool from the FASTX Toolkit v0.0.13 (http://hannonlab.cshl.edu/fastx_toolkit/). The raw sequencing data has been deposited to the NCBI Sequence Read Archive (accession number PRJNA769064). Trimmed reads were then processed using CellRanger software version 3.0.2 and the mm10 mouse genome reference for mapping, cell identification, and UMI processing (10x Genomics). Analyses for scRNA-seq were performed using R version 3.6.3 (single-cell level clustering and marker identification) or 4.1.2 (pseudobulk analysis).

Since sequencing on new-generation Illumina patterned flow cells can lead to the emergence of non-physiological chimeric reads, phantom molecules were identified and removed from the Cell Ranger h5 output using the PhantomPurge 1.0.0 R package (Farouni et al., 2020). After purging, data was converted to the ‘Seurat’ format for single-cell analysis for analysis with Seurat 3.2.2 package (Stuart et al., 2019). Genes detected in at least 50 cells, and cells with greater than 1000 genes and less than 10% of mitochondrial genes were selected for downstream analysis, yielding 15,415 cells and 11,622 genes passing quality control filters. The impact of number of genes detected per cells, percentage mitochondrial read, and cell cycle phase were regressed out as recommended by the Seurat package. We next leverage the DoubletFinder 2.0.3 package (McGinnis et al., 2019) to identify and filter out likely cell doublets, assuming a 2.3% doublet formation rate based on 10x Genomics estimates when capturing 3000 cells per samples. After this, 15,060 cells were identified as singlets by DoubletFinder and used for downstream analyses. Data was normalized using the SCTransform framework. We then ran principal component analysis, dimensionality reduction using UMAP using 30 principal components, and clustering with a resolution set to 0.2, yielding a total of eight clusters. Cluster markers were identified using the ‘FindAllMarkers’ function with a minimum of 25% of cells and a log_2_fold change threshold of 0.25 using the Wilcoxon test, and a significance threshold of FDR<0.05.

To analyze the scRNA-seq dataset with a pseudobulk approach, unnormalized counts were aggregated from cells within the same group (eight groups across age, sex, and MOTS-c treatment). Using R version 4.1.2 and the DESeq2 1.34.0 R package, differential transcriptomic profiles between the groups were estimated and visualized using MDS. To estimate the pairwise distance between female and male pseudobulk samples, we used a distance metric based on Spearman rank correlation coefficient (1–Rho). The distance of each female sample to both male samples was calculated in the control and MOTS-c-treated conditions. To determine whether the distance between female and male samples was reduced upon MOTS-c treatment (as hypothesized based on the MDS analysis), we then used a paired one-sided Wilcoxon rank-sum test to compare the distances in control vs*.* MOTS-c-treated conditions.

### BMDM single-cell GO cluster marker data analysis

To analyze which functional categories were enriched in association with BMDMs in clusters 5 and 6, whose frequency increased upon MOTS-c treatment, we used overrepresentation analysis with clusterProfiler 3.14.3 and annotation package org.Mm.eg.db 3.10.0. Enrichment was computed against the background of all expressed genes detected in the single-cell dataset.

### Code availability

The analytical code for the RNA-seq dataset is available on the Benayoun lab GitHub (https://github.com/BenayounLaboratory/MOTSc_Macrophage_Immunity, Benayoun, 2021). R code was run using R version 3.4.1, 3.6.3, or 4.1.2 as indicated in relevant sections.

## Data Availability

The raw sequencing data has been deposited to the NCBI Sequence Read Archive (accession number PRJNA623667 & PRJNA769064). The following datasets were generated: RiceMC ImunM JungSW ParkCY KimJS LaiRW BarrCR SonJM TorK KimE LuRJ CohenI BenayounBA LeeC 2020Impact of MOTS-c on gene expression in differentiating THP-1 monocytesNCBI BioProjectPRJNA623667 RiceMC ImunM JungSW ParkCY KimJS LaiRW BarrCR SonJM TorK KimE LuRJ CohenI BenayounBA LeeC 2021Single cell RNA-seq of murine Bone Marrow Derived Macrophages with and without treatment with MOTScNCBI BioProjectPRJNA769064

## Additional files

Supplementary file 1. Differentially regulated genes by phorbol myristate acetate (PMA) and MOTS-c in THP-1 cells.

Supplementary file 2. Gene set enrichment analysis (GSEA) of genes differentially regulated by MOTS-c in THP-1 cells.

Supplementary file 3. Genes enriched in clusters 5 and 6 in single-cell RNA-seq (scRNA-seq) of young and old bone marrow-derived macrophages of both sexes.

Supplementary file 4. Gene set enrichment analysis (GSEA) of genes enriched in clusters 5 and 6 in single-cell RNA-seq (scRNA-seq) of young and old bone marrow-derived macrophages of both sexes.

MDAR checklist

## References

[bib1] Almanzar N, Antony J, Baghel AS, Bakerman I, Bansal I, Barres BA, Beachy PA, Berdnik D, Bilen B, Brownfield D, Cain C, Chan CKF, Chen MB, Clarke MF, Conley SD, Darmanis S, Demers A, Demir K, de Morree A, Divita T, du Bois H, Ebadi H, Espinoza FH, Fish M, Gan Q, George BM, Gillich A, Gòmez-Sjöberg R, Green F, Genetiano G, Gu X, Gulati GS, Hahn O, Haney MS, Hang Y, Harris L, He M, Hosseinzadeh S, Huang A, Huang KC, Iram T, Isobe T, Ives F, Jones RC, Kao KS, Karkanias J, Karnam G, Keller A, Kershner AM, Khoury N, Kim SK, Kiss BM, Kong W, Krasnow MA, Kumar ME, Kuo CS, Lam J, Lee DP, Lee SE, Lehallier B, Leventhal O, Li G, Li Q, Liu L, Lo A, Lu WJ, Lugo-Fagundo MF, Manjunath A, May AP, Maynard A, McGeever A, McKay M, McNerney MW, Merrill B, Metzger RJ, Mignardi M, Min D, Nabhan AN, Neff NF, Ng KM, Nguyen PK, Noh J, Nusse R, Pálovics R, Patkar R, Peng WC, Penland L, Pisco AO, Pollard K, Puccinelli R, Qi Z, Quake SR, Rando TA, Rulifson EJ, Schaum N, Segal JM, Sikandar SS, Sinha R, Sit RV, Sonnenburg J, Staehli D, Szade K, Tan M, Tan W, Tato C, Tellez K, Dulgeroff LBT, Travaglini KJ, Tropini C, Tsui M, Waldburger L, Wang BM, van Weele LJ, Weinberg K, Weissman IL, Wosczyna MN, Wu SM, Wyss-Coray T, Xiang J, Xue S, Yamauchi KA, Yang AC, Yerra LP, Youngyunpipatkul J, Yu B, Zanini F, Zardeneta ME, Zee A, Zhao C, Zhang F, Zhang H, Zhang MJ, Zhou L, Zou J (2020). A single-cell transcriptomic atlas characterizes ageing tissues in the mouse. Nature.

[bib2] Amend SR, Valkenburg KC, Pienta KJ (2016). Murine hind limb long bone dissection and bone marrow isolation. Journal of Visualized Experiments.

[bib3] Andersson E, Rydengård V, Sonesson A, Mörgelin M, Björck L, Schmidtchen A (2004). Antimicrobial activities of heparin-binding peptides. European Journal of Biochemistry.

[bib4] Ashrafi N, Lapthorn C, Pullen FS, Naclerio F, Nielsen BV (2017). High performance liquid chromatography coupled to electrospray ionisation mass spectrometry method for the detection of salivary human neutrophil alpha defensins HNP1, HNP2, HNP3 and HNP4. Analytical Methods.

[bib5] Auwerx J (1991). The human leukemia cell line, THP-1: a multifacetted model for the study of monocyte-macrophage differentiation. Experientia.

[bib6] Ayabe T, Satchell DP, Wilson CL, Parks WC, Selsted ME, Ouellette AJ (2000). Secretion of microbicidal alpha-defensins by intestinal Paneth cells in response to bacteria. Nature Immunology.

[bib7] Bahar AA, Ren D (2013). Antimicrobial peptides. Pharmaceuticals.

[bib8] Bals R, Wang X, Zasloff M, Wilson JM (1998). The peptide antibiotic LL-37/hCAP-18 is expressed in epithelia of the human lung where it has broad antimicrobial activity at the airway surface. PNAS.

[bib9] Barman PK, Shin JE, Lewis SA, Kang S, Wu D, Wang Y, Yang X, Nagarkatti PS, Nagarkatti M, Messaoudi I, Benayoun BA, Goodridge HS (2022). Production of MHCII-expressing classical monocytes increases during aging in mice and humans. Aging Cell.

[bib10] Bassler BL (2002). Small talk. Cell-to-cell communication in bacteria. Cell.

[bib11] Benayoun BA, Pollina EA, Singh PP, Mahmoudi S, Harel I, Casey KM, Dulken BW, Kundaje A, Brunet A (2019). Remodeling of epigenome and transcriptome landscapes with aging in mice reveals widespread induction of inflammatory responses. Genome Research.

[bib12] Benayoun B (2021). Github.

[bib13] Biswas SK, Mantovani A (2012). Orchestration of metabolism by macrophages. Cell Metabolism.

[bib14] Bowdish DME, Davidson DJ, Speert DP, Hancock REW (2004). The human cationic peptide LL-37 induces activation of the extracellular signal-regulated kinase and p38 kinase pathways in primary human monocytes. Journal of Immunology.

[bib15] Bray NL, Pimentel H, Melsted P, Pachter L (2016). Near-optimal probabilistic RNA-seq quantification. Nature Biotechnology.

[bib16] Buszczak M, Signer RAJ, Morrison SJ (2014). Cellular differences in protein synthesis regulate tissue homeostasis. Cell.

[bib17] Cecotto L, van Kessel K, Wolfert MA, Vogely C, van der Wal B, Weinans H, van Strijp J, Amin Yavari S (2022). Antibacterial and anti-inflammatory properties of host defense peptides against *Staphylococcus aureus*. iScience.

[bib18] Chairatana P, Nolan EM (2014). Molecular basis for self-assembly of a human host-defense peptide that entraps bacterial pathogens. Journal of the American Chemical Society.

[bib19] Chang R, Subramanian K, Wang M, Webster TJ (2017). Enhanced antibacterial properties of self-assembling peptide amphiphiles functionalized with heparin-binding cardin-motifs. ACS Applied Materials & Interfaces.

[bib20] Che N, Qiu W, Wang JK, Sun XX, Xu LX, Liu R, Gu L (2019). MOTS-c improves osteoporosis by promoting the synthesis of type I collagen in osteoblasts via TGF-β/SMAD signaling pathway. European Review for Medical and Pharmacological Sciences.

[bib21] Chen Y, Meltzer PS (2005). Gene expression analysis via multidimensional scaling. Current Protocols in Bioinformatics.

[bib22] Chen J, Brunner A-D, Cogan JZ, Nuñez JK, Fields AP, Adamson B, Itzhak DN, Li JY, Mann M, Leonetti MD, Weissman JS (2020). Pervasive functional translation of noncanonical human open reading frames. Science.

[bib23] Choi H, Rangarajan N, Weisshaar JCL, Camera A (2016). Lights, camera, action! antimicrobial peptide mechanisms imaged in space and time. Trends in Microbiology.

[bib24] Chow NA, Jasenosky LD, Goldfeld AE (2014). A distal locus element mediates IFN-γ priming of lipopolysaccharide-stimulated TNF gene expression. Cell Reports.

[bib25] Cotter PD, Ross RP, Hill C (2013). Bacteriocins - a viable alternative to antibiotics?. Nature Reviews. Microbiology.

[bib26] Cudic M, Otvos L (2002). Intracellular targets of antibacterial peptides. Current Drug Targets.

[bib27] Davidson DJ, Currie AJ, Reid GSD, Bowdish DME, MacDonald KL, Ma RC, Hancock REW, Speert DP (2004). The cationic antimicrobial peptide LL-37 modulates dendritic cell differentiation and dendritic cell-induced T cell polarization. The Journal of Immunology.

[bib28] Drin G, Cottin S, Blanc E, Rees AR, Temsamani J (2003). Studies on the internalization mechanism of cationic cell-penetrating peptides. The Journal of Biological Chemistry.

[bib29] D’Souza RF, Woodhead JST, Hedges CP, Zeng N, Wan J, Kumagai H, Lee C, Cohen P, Cameron-Smith D, Mitchell CJ, Merry TL (2020). Increased expression of the mitochondrial derived peptide, MOTS-c, in skeletal muscle of healthy aging men is associated with myofiber composition. Aging.

[bib30] Duits LA, Ravensbergen B, Rademaker M, Hiemstra PS, Nibbering PH (2002). Expression of beta-defensin 1 and 2 mRNA by human monocytes, macrophages and dendritic cells. Immunology.

[bib31] Duplantier AJ, van Hoek ML (2013). The human cathelicidin antimicrobial peptide LL-37 as a potential treatment for polymicrobial infected wounds. Frontiers in Immunology.

[bib32] Fang XM, Shu Q, Chen QX, Book M, Sahl HG, Hoeft A, Stuber F (2003). Differential expression of alpha- and beta-defensins in human peripheral blood. European Journal of Clinical Investigation.

[bib33] Farkas A, Maróti G, Kereszt A, Kondorosi É (2017). Comparative analysis of the bacterial membrane disruption effect of two natural plant antimicrobial peptides. Frontiers in Microbiology.

[bib34] Farouni R, Djambazian H, Ferri LE, Ragoussis J, Najafabadi HS (2020). Model-based analysis of sample index hopping reveals its widespread artifacts in multiplexed single-cell RNA-sequencing. Nature Communications.

[bib35] Fei F, Lee KM, McCarry BE, Bowdish DME (2016). Age-associated metabolic dysregulation in bone marrow-derived macrophages stimulated with lipopolysaccharide. Scientific Reports.

[bib36] Franceschi C, Garagnani P, Parini P, Giuliani C, Santoro A (2018). Inflammaging: a new immune-metabolic viewpoint for age-related diseases. Nature Reviews. Endocrinology.

[bib37] Fuku N, Pareja-Galeano H, Zempo H, Alis R, Arai Y, Lucia A, Hirose N (2015). The mitochondrial-derived peptide MOTS-c: a player in exceptional longevity?. Aging Cell.

[bib38] Gagnon KT, Li L, Janowski BA, Corey DR (2014). Analysis of nuclear RNA interference in human cells by subcellular fractionation and Argonaute loading. Nature Protocols.

[bib39] Galluzzi L, Yamazaki T, Kroemer G (2018). Linking cellular stress responses to systemic homeostasis. Nature Reviews. Molecular Cell Biology.

[bib40] Ginhoux F, Schultze JL, Murray PJ, Ochando J, Biswas SK (2016). New insights into the multidimensional concept of macrophage ontogeny, activation and function. Nature Immunology.

[bib41] Gläser R, Harder J, Lange H, Bartels J, Christophers E, Schröder J-M (2005). Antimicrobial psoriasin (S100A7) protects human skin from *Escherichia coli* infection. Nature Immunology.

[bib42] Gomes B, Augusto MT, Felício MR, Hollmann A, Franco OL, Gonçalves S, Santos NC (2018). Designing improved active peptides for therapeutic approaches against infectious diseases. Biotechnology Advances.

[bib43] Goronzy JJ, Weyand CM (2013). Understanding immunosenescence to improve responses to vaccines. Nature Immunology.

[bib44] Gubert C, Hannan AJ (2021). Exercise mimetics: harnessing the therapeutic effects of physical activity. Nature Reviews. Drug Discovery.

[bib45] Hancock REW, Haney EF, Gill EE (2016). The immunology of host defence peptides: beyond antimicrobial activity. Nature Reviews. Immunology.

[bib46] Hanson MA, Dostálová A, Ceroni C, Poidevin M, Kondo S, Lemaitre B (2019). Synergy and remarkable specificity of antimicrobial peptides in vivo using a systematic knockout approach. eLife.

[bib47] Harder J, Bartels J, Christophers E, Schröder JM (1997). A peptide antibiotic from human skin. Nature.

[bib48] Held TK, Weihua X, Yuan L, Kalvakolanu DV, Cross AS (1999). Gamma interferon augments macrophage activation by lipopolysaccharide by two distinct mechanisms, at the signal transduction level and via an autocrine mechanism involving tumor necrosis factor alpha and interleukin-1. Infection and Immunity.

[bib49] Ho YH, Shah P, Chen YW, Chen CS (2016). Systematic analysis of intracellular-targeting antimicrobial peptides, Bactenecin 7, Hybrid of Pleurocidin and Dermaseptin, Proline-Arginine-rich Peptide, and Lactoferricin B, by Using *Escherichia coli* proteome microarrays. Molecular & Cellular Proteomics.

[bib50] Hu BT, Chen WZ (2018). MOTS-c improves osteoporosis by promoting osteogenic differentiation of bone marrow mesenchymal stem cells via TGF-β/Smad pathway. European Review for Medical and Pharmacological Sciences.

[bib51] Hurdle JG, O’Neill AJ, Chopra I, Lee RE (2011). Targeting bacterial membrane function: an underexploited mechanism for treating persistent infections. Nature Reviews. Microbiology.

[bib52] Ingelsson B, Söderberg D, Strid T, Söderberg A, Bergh AC, Loitto V, Lotfi K, Segelmark M, Spyrou G, Rosén A (2018). Lymphocytes eject interferogenic mitochondrial DNA webs in response to CpG and non-CpG oligodeoxynucleotides of class C. PNAS.

[bib53] Ingolia NT, Lareau LF, Weissman JS (2011). Ribosome profiling of mouse embryonic stem cells reveals the complexity and dynamics of mammalian proteomes. Cell.

[bib54] Kandasamy SK, Larson RG (2006). Effect of salt on the interactions of antimicrobial peptides with zwitterionic lipid bilayers. Biochimica et Biophysica Acta (BBA) - Biomembranes.

[bib55] Kang GM, Min SH, Lee CH, Kim JY, Lim HS, Choi MJ, Jung S-B, Park JW, Kim S, Park CB, Dugu H, Choi JH, Jang WH, Park SE, Cho YM, Kim JG, Kim K-G, Choi CS, Kim Y-B, Lee C, Shong M, Kim M-S (2021). Mitohormesis in hypothalamic POMC neurons mediates regular exercise-induced high-turnover metabolism. Cell Metabolism.

[bib56] Keller L, Surette MG (2006). Communication in bacteria: an ecological and evolutionary perspective. Nature Reviews. Microbiology.

[bib57] Khajuria RK, Munschauer M, Ulirsch JC, Fiorini C, Ludwig LS, McFarland SK, Abdulhay NJ, Specht H, Keshishian H, Mani DR, Jovanovic M, Ellis SR, Fulco CP, Engreitz JM, Schütz S, Lian J, Gripp KW, Weinberg OK, Pinkus GS, Gehrke L, Regev A, Lander ES, Gazda HT, Lee WY, Panse VG, Carr SA, Sankaran VG (2018). Ribosome levels selectively regulate translation and lineage commitment in human hematopoiesis. Cell.

[bib58] Kim SJ, Xiao J, Wan J, Cohen P, Yen K (2017). Mitochondrially derived peptides as novel regulators of metabolism. The Journal of Physiology.

[bib59] Kim KH, Son JM, Benayoun BA, Lee C (2018). The mitochondrial-encoded peptide MOTS-c translocates to the nucleus to regulate nuclear gene expression in response to metabolic stress. Cell Metabolism.

[bib60] Kim S-J, Miller B, Mehta HH, Xiao J, Wan J, Arpawong TE, Yen K, Cohen P (2019). The mitochondrial-derived peptide MOTS-c is a regulator of plasma metabolites and enhances insulin sensitivity. Physiological Reports.

[bib61] Kimmel JC, Penland L, Rubinstein ND, Hendrickson DG, Kelley DR, Rosenthal AZ (2019). Murine single-cell RNA-seq reveals cell-identity- and tissue-specific trajectories of aging. Genome Research.

[bib62] Kolde R (2015). R Package Version.

[bib63] Kong BS, Min SH, Lee C, Cho YM (2021). Mitochondrial-encoded MOTS-c prevents pancreatic islet destruction in autoimmune diabetes. Cell Reports.

[bib64] Kyte J, Doolittle RF (1982). A simple method for displaying the hydropathic character of a protein. Journal of Molecular Biology.

[bib65] Lai Y, Gallo RL (2009). AMPed up immunity: how antimicrobial peptides have multiple roles in immune defense. Trends in Immunology.

[bib66] Langston PK, Shibata M, Horng T (2017). Metabolism supports macrophage activation. Frontiers in Immunology.

[bib67] Lazzaro BP, Zasloff M, Rolff J (2020). Antimicrobial peptides: application informed by evolution. Science.

[bib68] Le CF, Fang CM, Sekaran SD (2017). Intracellular targeting mechanisms by antimicrobial peptides. Antimicrobial Agents and Chemotherapy.

[bib69] Lee C, Yen K, Cohen P (2013). Humanin: a harbinger of mitochondrial-derived peptides?. Trends in Endocrinology and Metabolism.

[bib70] Lee C, Zeng J, Drew BG, Sallam T, Martin-Montalvo A, Wan J, Kim S-J, Mehta H, Hevener AL, de Cabo R, Cohen P (2015). The mitochondrial-derived peptide MOTS-c promotes metabolic homeostasis and reduces obesity and insulin resistance. Cell Metabolism.

[bib71] Lehrer RI, Ganz T (1992). Defensins: endogenous antibiotic peptides from human leukocytes. Ciba Foundation Symposium.

[bib72] Lewis K (2013). Platforms for antibiotic discovery. Nature Reviews. Drug Discovery.

[bib73] Li Q, Lu H, Hu G, Ye Z, Zhai D, Yan Z, Wang L, Xiang A, Lu Z (2019). Earlier changes in mice after D-galactose treatment were improved by mitochondria derived small peptide MOTS-c. Biochemical and Biophysical Research Communications.

[bib74] Liao MH, Chen SJ, Tsao CM, Shih CC, Wu CC (2013). Possible biomarkers of early mortality in peritonitis-induced sepsis rats. The Journal of Surgical Research.

[bib75] Liu L, Roberts AA, Ganz T (2003). By IL-1 signaling, monocyte-derived cells dramatically enhance the epidermal antimicrobial response to lipopolysaccharide. The Journal of Immunology.

[bib76] Liu C, Gidlund E-K, Witasp A, Qureshi AR, Söderberg M, Thorell A, Nader GA, Barany P, Stenvinkel P, von Walden F (2019). Reduced skeletal muscle expression of mitochondrial-derived peptides humanin and MOTS-C and Nrf2 in chronic kidney disease. American Journal of Physiology-Renal Physiology.

[bib77] Lloberas J, Celada A (2002). Effect of aging on macrophage function. Experimental Gerontology.

[bib78] López-Otín C, Galluzzi L, Freije JMP, Madeo F, Kroemer G (2016). Metabolic control of longevity. Cell.

[bib79] López-Otín C, Blasco MA, Partridge L, Serrano M, Kroemer G (2023). Hallmarks of aging: an expanding universe. Cell.

[bib80] Love MI, Huber W, Anders S (2014). Moderated estimation of fold change and dispersion for RNA-seq data with DESeq2. Genome Biology.

[bib81] Lu H, Tang S, Xue C, Liu Y, Wang J, Zhang W, Luo W, Chen J (2019a). Mitochondrial-derived peptide MOTS-c increases adipose thermogenic activation to promote cold adaptation. International Journal of Molecular Sciences.

[bib82] Lu H, Wei M, Zhai Y, Li Q, Ye Z, Wang L, Luo W, Chen J, Lu Z (2019b). MOTS-c peptide regulates adipose homeostasis to prevent ovariectomy-induced metabolic dysfunction. Journal of Molecular Medicine.

[bib83] Lu R, Sampathkumar NK, Benayoun BA (2020). Measuring phagocytosis in bone marrow-derived macrophages and peritoneal macrophages with aging. Methods in Molecular Biology.

[bib84] Mahbub S, Deburghgraeve CR, Kovacs EJ (2012). Advanced age impairs macrophage polarization. Journal of Interferon & Cytokine Research.

[bib85] Malanovic N, Lohner K (2016). Gram-positive bacterial cell envelopes: the impact on the activity of antimicrobial peptides. Biochimica et Biophysica Acta (BBA) - Biomembranes.

[bib86] Martijn J, Vosseberg J, Guy L, Offre P, Ettema TJG (2018). Deep mitochondrial origin outside the sampled alphaproteobacteria. Nature.

[bib87] McGinnis CS, Murrow LM, Gartner ZJ (2019). DoubletFinder: doublet detection in single-cell RNA sequencing data using artificial nearest neighbors. Cell Systems.

[bib88] Melo MN, Ferre R, Castanho MARB (2009). Antimicrobial peptides: linking partition, activity and high membrane-bound concentrations. Nature Reviews. Microbiology.

[bib89] Miller B, Kim SJ, Kumagai H, Yen K, Cohen P (2022). Mitochondria-derived peptides in aging and healthspan. The Journal of Clinical Investigation.

[bib90] Ming W, Lu G, Xin S, Huanyu L, Yinghao J, Xiaoying L, Chengming X, Banjun R, Li W, Zifan L (2016). Mitochondria related peptide MOTS-c suppresses ovariectomy-induced bone loss via AMPK activation. Biochemical and Biophysical Research Communications.

[bib91] Minhas PS, Liu L, Moon PK, Joshi AU, Dove C, Mhatre S, Contrepois K, Wang Q, Lee BA, Coronado M, Bernstein D, Snyder MP, Migaud M, Majeti R, Mochly-Rosen D, Rabinowitz JD, Andreasson KI (2019). Macrophage de novo NAD^+^ synthesis specifies immune function in aging and inflammation. Nature Immunology.

[bib92] Mishra B, Reiling S, Zarena D, Wang G (2017). Host defense antimicrobial peptides as antibiotics: design and application strategies. Current Opinion in Chemical Biology.

[bib93] Monnet V, Juillard V, Gardan R (2016). Peptide conversations in Gram-positive bacteria. Critical Reviews in Microbiology.

[bib94] Mookherjee N, Anderson MA, Haagsman HP, Davidson DJ (2020). Antimicrobial host defence peptides: functions and clinical potential. Nature Reviews. Drug Discovery.

[bib95] Mottis A, Herzig S, Auwerx J (2019). Mitocellular communication: shaping health and disease. Science.

[bib96] Müller E, Christopoulos PF, Halder S, Lunde A, Beraki K, Speth M, Øynebråten I, Corthay A (2017). Toll-like receptor ligands and interferon-γ synergize for induction of antitumor M1 macrophages. Frontiers in Immunology.

[bib97] Murray PJ (2017). Macrophage polarization. Annual Review of Physiology.

[bib98] Nakagomi A, Freedman SB, Geczy CL (2000). Interferon-γ and lipopolysaccharide potentiate monocyte tissue factor induction by C-reactive protein. Circulation.

[bib99] Nicolas P (2009). Multifunctional host defense peptides: intracellular-targeting antimicrobial peptides. The FEBS Journal.

[bib100] Odegaard JI, Chawla A (2011). Alternative macrophage activation and metabolism. Annual Review of Pathology.

[bib101] Otto NA, Butler JM, Ramirez-Moral I, van Weeghel M, van Heijst JWJ, Scicluna BP, Houtkooper RH, de Vos AF, van der Poll T (2021). Adherence affects monocyte innate immune function and metabolic reprogramming after lipopolysaccharide stimulation in vitro. Journal of Immunology.

[bib102] Panda A, Arjona A, Sapey E, Bai F, Fikrig E, Montgomery RR, Lord JM, Shaw AC (2009). Human innate immunosenescence: causes and consequences for immunity in old age. Trends in Immunology.

[bib103] Papayannopoulos V (2018). Neutrophil extracellular traps in immunity and disease. Nature Reviews. Immunology.

[bib104] Pawelec G (2017). Does the human immune system ever really become “senescent”?. F1000Research.

[bib105] Pawelec G (2018). Age and immunity: What is “immunosenescence”?. Experimental Gerontology.

[bib106] Pena OM, Afacan N, Pistolic J, Chen C, Madera L, Falsafi R, Fjell CD, Hancock REW (2013). Synthetic cationic peptide IDR-1018 modulates human macrophage differentiation. PLOS ONE.

[bib107] Pioli PA, Weaver LK, Schaefer TM, Wright JA, Wira CR, Guyre PM (2006). Lipopolysaccharide-induced IL-1 beta production by human uterine macrophages up-regulates uterine epithelial cell expression of human beta-defensin 2. Journal of Immunology.

[bib108] Plowden J, Renshaw-Hoelscher M, Engleman C, Katz J, Sambhara S (2004). Innate immunity in aging: impact on macrophage function. Aging Cell.

[bib109] Pulido D, Moussaoui M, Andreu D, Nogués MV, Torrent M, Boix E (2012). Antimicrobial action and cell agglutination by the eosinophil cationic protein are modulated by the cell wall lipopolysaccharide structure. Antimicrobial Agents and Chemotherapy.

[bib110] Quirós PM, Mottis A, Auwerx J (2016). Mitonuclear communication in homeostasis and stress. Nature Reviews. Molecular Cell Biology.

[bib111] Reynolds J (2019). MOTS-c is an exercise-induced mitochondrial-encoded regulator of age-dependent physical decline and muscle homeostasis. bioRxiv.

[bib112] Reynolds JC, Bwiza CP, Lee C (2020). Mitonuclear genomics and aging. Human Genetics.

[bib113] Reynolds JC, Lai RW, Woodhead JST, Joly JH, Mitchell CJ, Cameron-Smith D, Lu R, Cohen P, Graham NA, Benayoun BA, Merry TL, Lee C (2021). MOTS-c is an exercise-induced mitochondrial-encoded regulator of age-dependent physical decline and muscle homeostasis. Nature Communications.

[bib114] Robert É, Lefèvre T, Fillion M, Martial B, Dionne J, Auger M (2015). Mimicking and understanding the agglutination effect of the antimicrobial peptide thanatin using model phospholipid vesicles. Biochemistry.

[bib115] Roth BL, Poot M, Yue ST, Millard PJ (1997). Bacterial viability and antibiotic susceptibility testing with SYTOX green nucleic acid stain. Applied and Environmental Microbiology.

[bib116] Russell DG, Huang L, VanderVen BC (2019). Immunometabolism at the interface between macrophages and pathogens. Nature Reviews. Immunology.

[bib117] Saghatelian A, Couso JP (2015). Discovery and characterization of smORF-encoded bioactive polypeptides. Nature Chemical Biology.

[bib118] Sassone-Corsi M, Nuccio S-P, Liu H, Hernandez D, Vu CT, Takahashi AA, Edwards RA, Raffatellu M (2016). Microcins mediate competition among Enterobacteriaceae in the inflamed gut. Nature.

[bib119] Schmidt NW, Mishra A, Lai GH, Davis M, Sanders LK, Tran D, Garcia A, Tai KP, McCray PB, Ouellette AJ, Selsted ME, Wong GCL (2011). Criterion for amino acid composition of defensins and antimicrobial peptides based on geometry of membrane destabilization. Journal of the American Chemical Society.

[bib120] Schmidt NW, Wong GCL (2013). Antimicrobial peptides and induced membrane curvature: geometry, coordination chemistry, and molecular engineering. Current Opinion in Solid State and Materials Science.

[bib121] Schroder K, Hertzog PJ, Ravasi T, Hume DA (2004). Interferon-γ: an overview of signals, mechanisms and functions. Journal of Leukocyte Biology.

[bib122] Shah P, Hsiao FSH, Ho YH, Chen CS (2016). The proteome targets of intracellular targeting antimicrobial peptides. Proteomics.

[bib123] Shi Z, Barna M (2015). Translating the genome in time and space: specialized ribosomes, RNA regulons, and RNA-binding proteins. Annual Review of Cell and Developmental Biology.

[bib124] Sinha S, Zheng L, Mu Y, Ng WJ, Bhattacharjya S (2017). Structure and interactions of a host defense antimicrobial peptide thanatin in lipopolysaccharide micelles reveal mechanism of bacterial cell agglutination. Scientific Reports.

[bib125] Sloan DB, Warren JM, Williams AM, Wu Z, Abdel-Ghany SE, Chicco AJ, Havird JC (2018). Cytonuclear integration and co-evolution. Nature Reviews. Genetics.

[bib126] Sørensen O, Cowland JB, Askaa J, Borregaard N (1997). An ELISA for hCAP-18, the cathelicidin present in human neutrophils and plasma. Journal of Immunological Methods.

[bib127] Sørensen OE, Follin P, Johnsen AH, Calafat J, Tjabringa GS, Hiemstra PS, Borregaard N (2001). Human cathelicidin, hCAP-18, is processed to the antimicrobial peptide LL-37 by extracellular cleavage with proteinase 3. Blood.

[bib128] Squair JW, Gautier M, Kathe C, Anderson MA, James ND, Hutson TH, Hudelle R, Qaiser T, Matson KJE, Barraud Q, Levine AJ, La Manno G, Skinnider MA, Courtine G (2021). Confronting false discoveries in single-cell differential expression. Nature Communications.

[bib129] Stuart T, Butler A, Hoffman P, Hafemeister C, Papalexi E, Mauck WM, Hao Y, Stoeckius M, Smibert P, Satija R (2019). Comprehensive integration of single-cell data. Cell.

[bib130] Subramanian A, Tamayo P, Mootha VK, Mukherjee S, Ebert BL, Gillette MA, Paulovich A, Pomeroy SL, Golub TR, Lander ES, Mesirov JP (2005). Gene set enrichment analysis: a knowledge-based approach for interpreting genome-wide expression profiles. PNAS.

[bib131] Sun J, Furio L, Mecheri R, van der Does AM, Lundeberg E, Saveanu L, Chen Y, van Endert P, Agerberth B, Diana J (2015). Pancreatic β-Cells limit autoimmune diabetes via an immunoregulatory antimicrobial peptide expressed under the influence of the gut microbiota. Immunity.

[bib132] Sun Y, Shang D (2015). Inhibitory effects of antimicrobial peptides on lipopolysaccharide-induced inflammation. Mediators of Inflammation.

[bib133] Sweet RM, Eisenberg D (1983). Correlation of sequence hydrophobicities measures similarity in three-dimensional protein structure. Journal of Molecular Biology.

[bib134] Szklarczyk D, Gable AL, Lyon D, Junge A, Wyder S, Huerta-Cepas J, Simonovic M, Doncheva NT, Morris JH, Bork P, Jensen LJ, Mering CV (2019). STRING v11: protein-protein association networks with increased coverage, supporting functional discovery in genome-wide experimental datasets. Nucleic Acids Research.

[bib135] Tamai R, Sugawara S, Takeuchi O, Akira S, Takada H (2003). Synergistic effects of lipopolysaccharide and interferon-gamma in inducing interleukin-8 production in human monocytic THP-1 cells is accompanied by up-regulation of CD14, Toll-like receptor 4, MD-2 and MyD88 expression. Journal of Endotoxin Research.

[bib136] Tan JX, Finkel T (2020). Mitochondria as intracellular signaling platforms in health and disease. The Journal of Cell Biology.

[bib137] Teo YV, Hinthorn SJ, Webb AE, Neretti N (2023). Single-cell transcriptomics of peripheral blood in the aging mouse. Aging.

[bib138] Thomas S, Karnik S, Barai RS, Jayaraman VK, Idicula-Thomas S (2010). CAMP: a useful resource for research on antimicrobial peptides. Nucleic Acids Research.

[bib139] Torrent M, Pulido D, Nogués MV, Boix E (2012). Exploring new biological functions of amyloids: bacteria cell agglutination mediated by host protein aggregation. PLOS Pathogens.

[bib140] Tsutsumi-Ishii Y, Nagaoka I (2003). Modulation of human beta-defensin-2 transcription in pulmonary epithelial cells by lipopolysaccharide-stimulated mononuclear phagocytes via proinflammatory cytokine production. Journal of Immunology.

[bib141] Tsuzuki T, Nomiyama H, Setoyama C, Maeda S, Shimada K, Pestka S (1983). The majority of cDNA clones with strong positive signals for the interferon-induction-specific sequences resemble mitochondrial ribosomal RNA genes. Biochemical and Biophysical Research Communications.

[bib142] Tyers M, Wright GD (2019). Drug combinations: a strategy to extend the life of antibiotics in the 21st century. Nature Reviews. Microbiology.

[bib143] van Beek AA, Van den Bossche J, Mastroberardino PG, de Winther MPJ, Leenen PJM (2019). Metabolic alterations in aging macrophages: ingredients for inflammaging?. Trends in Immunology.

[bib144] van der Does AM, Beekhuizen H, Ravensbergen B, Vos T, Ottenhoff THM, van Dissel JT, Drijfhout JW, Hiemstra PS, Nibbering PH (2010). LL-37 directs macrophage differentiation toward macrophages with a proinflammatory signature. Journal of Immunology.

[bib145] Viola A, Munari F, Sánchez-Rodríguez R, Scolaro T, Castegna A (2019). The metabolic signature of macrophage responses. Frontiers in Immunology.

[bib146] Wei M, Gan L, Liu Z, Liu L, Chang J-R, Yin D-C, Cao H-L, Su X-L, Smith WW (2020). Mitochondrial-derived peptide MOTS-c attenuates vascular calcification and secondary myocardial remodeling via adenosine monophosphate-activated protein kinase signaling pathway. Cardiorenal Medicine.

[bib147] Weng FB, Zhu LF, Zhou JX, Shan Y, Tian ZG, Yang LW (2019). MOTS-c accelerates bone fracture healing by stimulating osteogenesis of bone marrow mesenchymal stem cells via positively regulating FOXF1 to activate the TGF-β pathway. European Review for Medical and Pharmacological Sciences.

[bib148] West AP, Shadel GS (2017). Mitochondrial DNA in innate immune responses and inflammatory pathology. Nature Reviews. Immunology.

[bib149] Xinqiang Y, Quan C, Yuanyuan J, Hanmei X (2020). Protective effect of MOTS-c on acute lung injury induced by lipopolysaccharide in mice. International Immunopharmacology.

[bib150] Xue J, Schmidt SV, Sander J, Draffehn A, Krebs W, Quester I, De Nardo D, Gohel TD, Emde M, Schmidleithner L, Ganesan H, Nino-Castro A, Mallmann MR, Labzin L, Theis H, Kraut M, Beyer M, Latz E, Freeman TC, Ulas T, Schultze JL (2014). Transcriptome-based network analysis reveals a spectrum model of human macrophage activation. Immunity.

[bib151] Yan Z, Zhu S, Wang H, Wang L, Du T, Ye Z, Zhai D, Zhu Z, Tian X, Lu Z, Cao X (2019). MOTS-c inhibits osteolysis in the mouse calvaria by affecting osteocyte-osteoclast crosstalk and inhibiting inflammation. Pharmacological Research.

[bib152] Yin X, Jing Y, Chen Q, Abbas AB, Hu J, Xu H (2020). The intraperitoneal administration of MOTS-c produces antinociceptive and anti-inflammatory effects through the activation of AMPK pathway in the mouse formalin test. European Journal of Pharmacology.

[bib153] Yousefi S, Gold JA, Andina N, Lee JJ, Kelly AM, Kozlowski E, Schmid I, Straumann A, Reichenbach J, Gleich GJ, Simon H-U (2008). Catapult-like release of mitochondrial DNA by eosinophils contributes to antibacterial defense. Nature Medicine.

[bib154] Zasloff M (2002). Antimicrobial peptides of multicellular organisms. Nature.

[bib155] Zempo H, Kim SJ, Fuku N, Nishida Y, Higaki Y, Wan J, Yen K, Miller B, Vicinanza R, Miyamoto-Mikami E, Kumagai H, Naito H, Xiao J, Mehta HH, Lee C, Hara M, Patel YM, Setiawan VW, Moore TM, Hevener AL, Sutoh Y, Shimizu A, Kojima K, Kinoshita K, Arai Y, Hirose N, Maeda S, Tanaka K, Cohen P (2021). A pro-diabetogenic mtDNA polymorphism in the mitochondrial-derived peptide, MOTS-c. Aging.

[bib156] Zhai D, Ye Z, Jiang Y, Xu C, Ruan B, Yang Y, Lei X, Xiang A, Lu H, Zhu Z, Yan Z, Wei D, Li Q, Wang L, Lu Z (2017). MOTS-c peptide increases survival and decreases bacterial load in mice infected with MRSA. Molecular Immunology.

[bib157] Zhai Z, Boquete JP, Lemaitre B (2018). Cell-Specific Imd-NF-κB responses enable simultaneous antibacterial immunity and intestinal epithelial cell shedding upon bacterial infection. Immunity.

[bib158] Zhang LJ, Gallo RL (2016). Antimicrobial peptides. Current Biology.

