# Supplementary material for: MOTS-c is a mitochondrial-encoded interferon-linked host defense peptide
Source: eLife. 2026 Aug 18;12:RP87615. doi: 10.7554/eLife.87615 (PMC13485304; doi:10.7554/eLife.87615)
Supplement: Supplementary file 2 [file elife-87615-fig1-figsupp1-data1.zip › Figure 1-figure supplement 1_Source Data 1/Figure 1-figure supplement 1A_Source Data 1.pdf]

The Human Mitochondrial Genome Encodes for an Interferon-Responsive Host Defense Peptide

Rice MC, Kim JS, Imun M, Jung SW, Park CY, Lai RW, Barr CR, Son JM, Tor K, Kim E, Lu RJ, Cohen I, Benayoun BA, and Lee C

Figure 1-figure supplement 1A

A

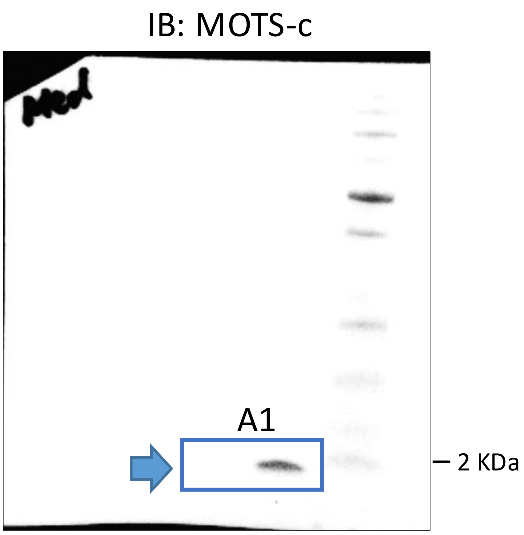

<u>A1:</u>
MOTS-c left in the media after mixing with *E. coli*.

B

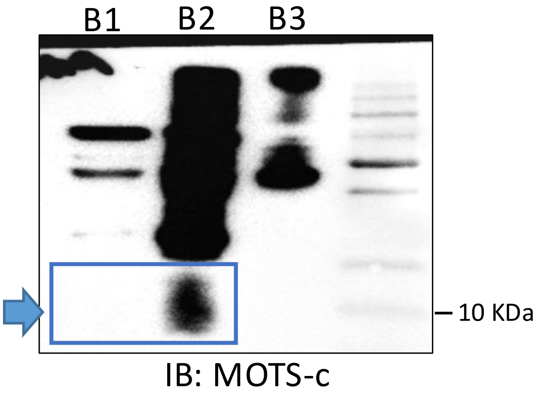

<u>B1:</u>
E. coli untreated with MOTS-c.

<u>B2:</u>
E. coli treated with MOTS-c.

<u>B3:</u>
E. coli treated with MOTS-c-FLAG

Upper band suspected to be non-specific binding of MOTS-c peptide to *E. coli* proteins.

C

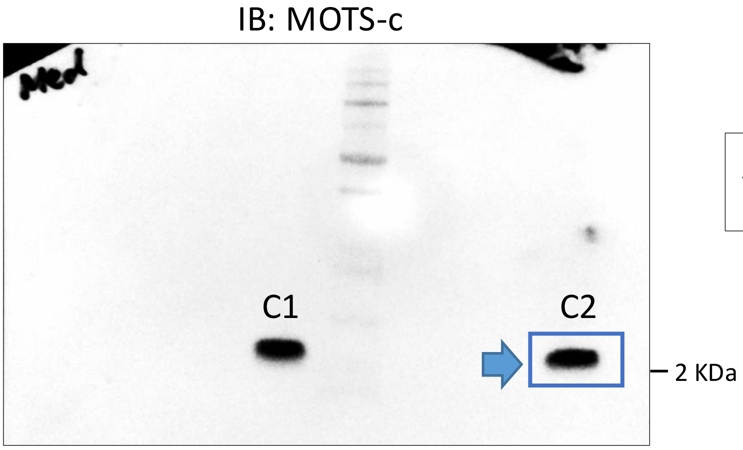

<u>C1 & C2:</u>
MOTS-c in media only.
