# Supplementary material for: MOTS-c is a mitochondrial-encoded interferon-linked host defense peptide
Source: eLife. 2026 Aug 18;12:RP87615. doi: 10.7554/eLife.87615 (PMC13485304; doi:10.7554/eLife.87615)

The Human Mitochondrial Genome Encodes for an Interferon-Responsive Host Defense Peptide

Rice MC, Kim JS, Imun M, Jung SW, Park CY, Lai RW, Barr CR, Son JM,
Tor K, Kim E, Lu RJ, Cohen I, Benayoun BA, and Lee C

Fig 3A

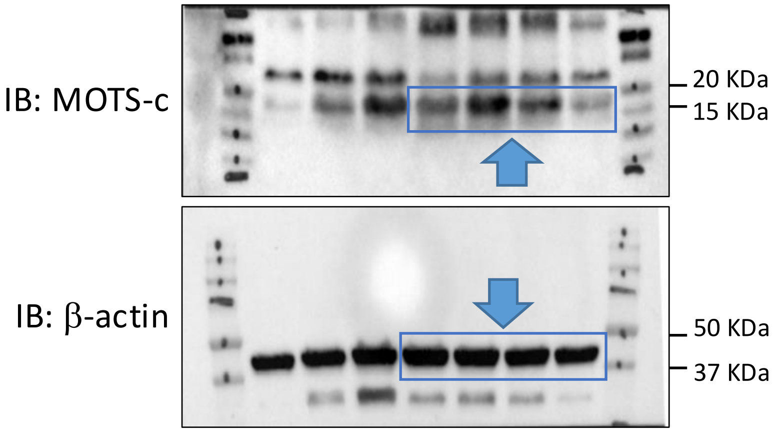

Fig 3B

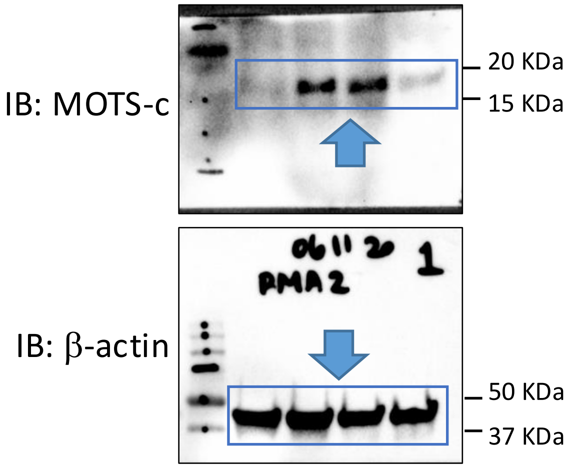

Fig 3C

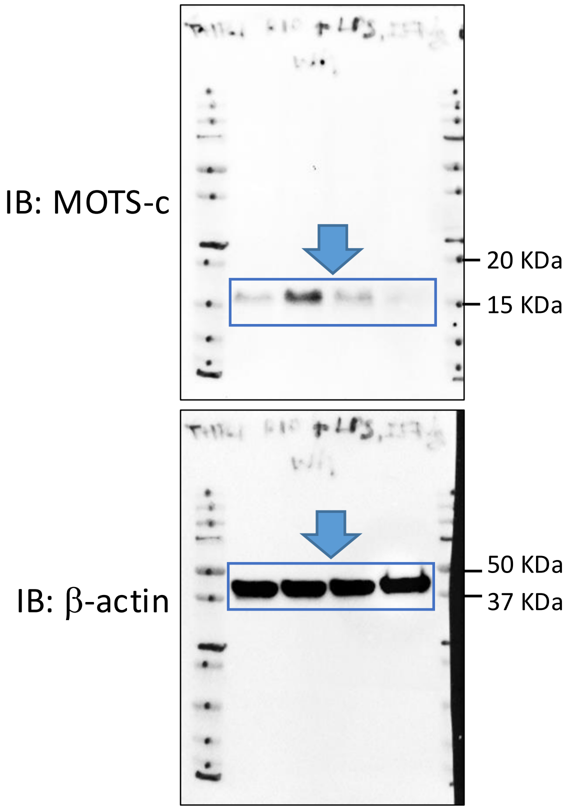

Fig 3D

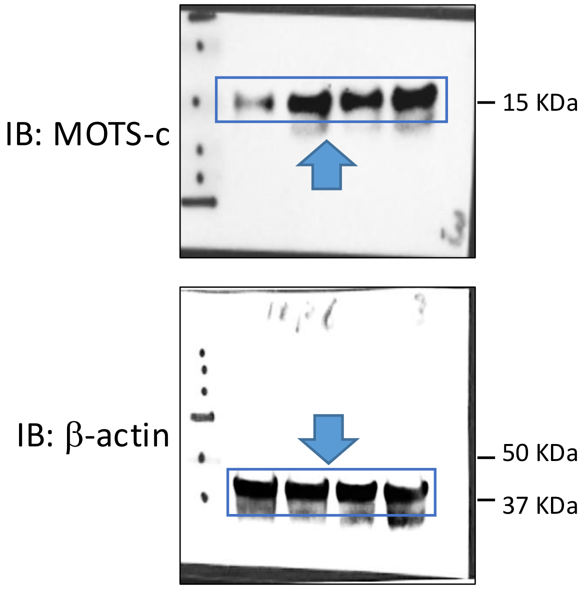

Fig 3E

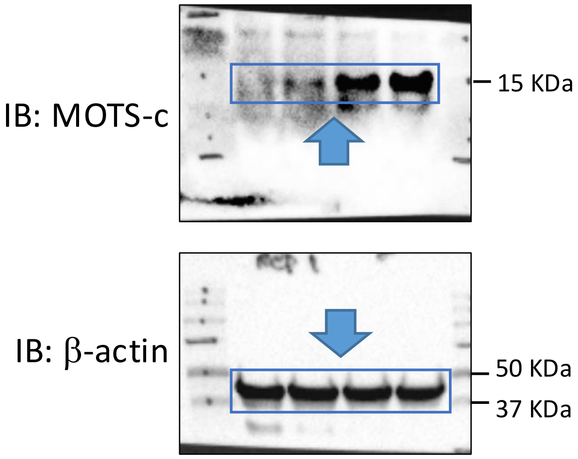

Supplement: Supplementary file 4 [file elife-87615-fig3-data1.zip › Figure 3_Source Data 1/Figure 3_Source Data 1.pdf]
