# Supplementary material for: MOTS-c is a mitochondrial-encoded interferon-linked host defense peptide
Source: eLife. 2026 Aug 18;12:RP87615. doi: 10.7554/eLife.87615 (PMC13485304; doi:10.7554/eLife.87615)

The Human Mitochondrial Genome Encodes for an Interferon-Responsive Host Defense Peptide

Rice MC, Kim JS, Imun M, Jung SW, Park CY, Lai RW, Barr CR, Son JM, Tor K, Kim E, Lu RJ, Cohen I, Benayoun BA, and Lee C

Figure 3-figure supplement 1A

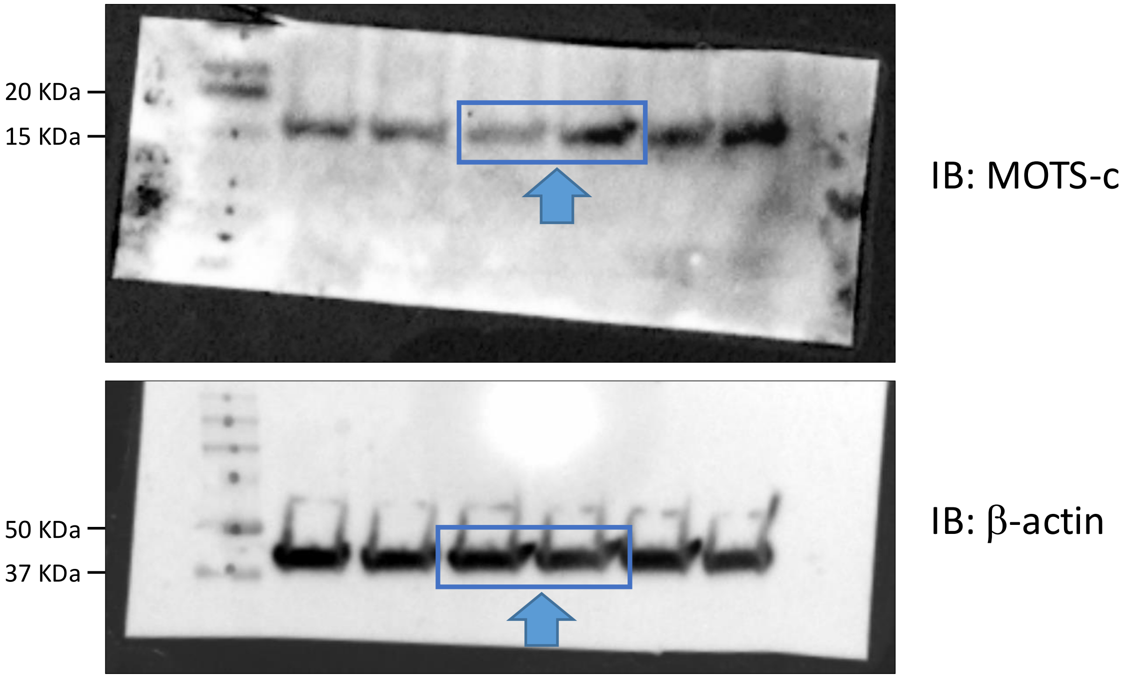

Figure 3-figure supplement 1B

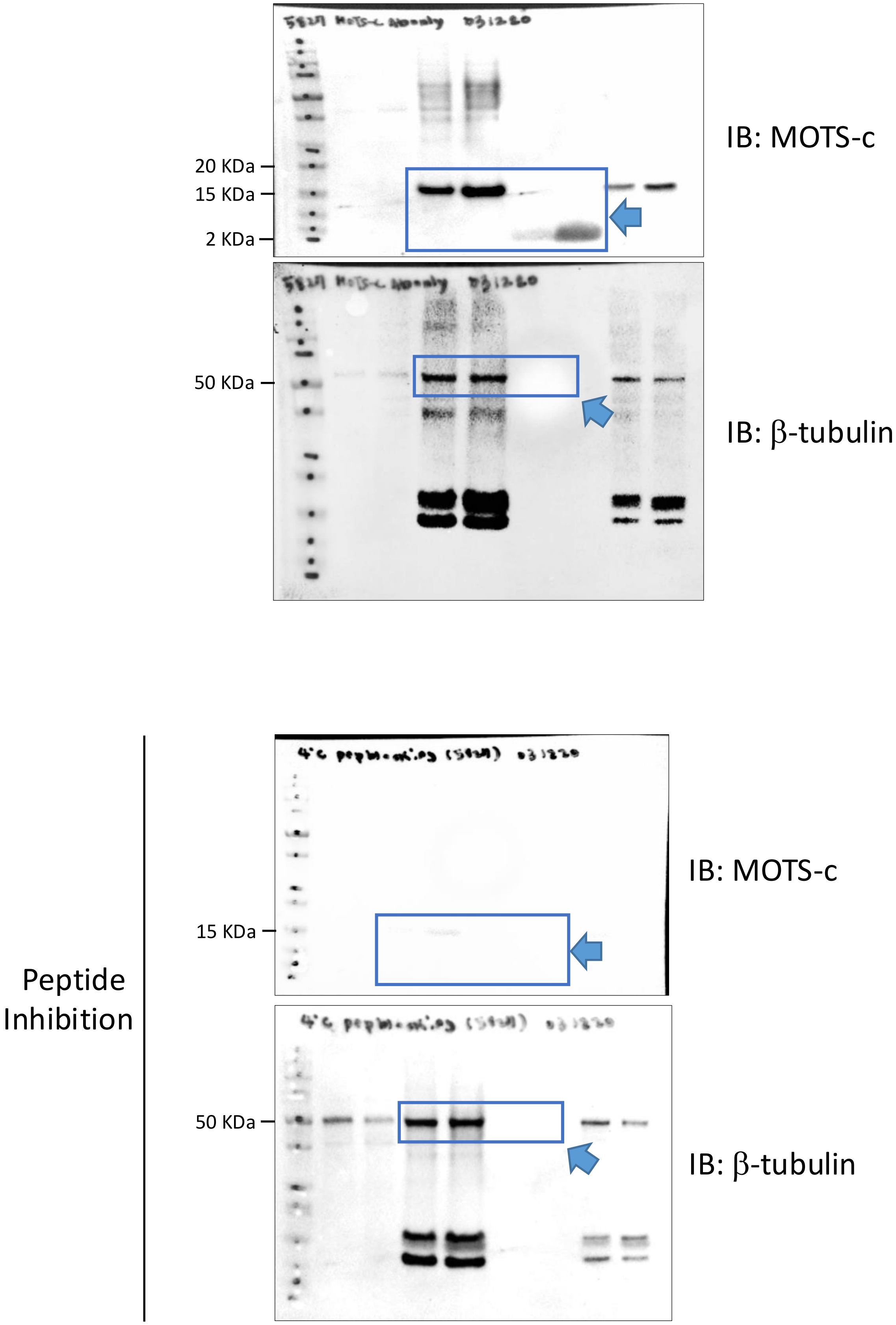

Supplement: Supplementary file 6 [file elife-87615-fig3-figsupp1-data1.zip › Figure 3-figure supplement 1_Source Data 1/Figure 3-figure supplement 1_Source Data 1.pdf]
