# Supplementary material for: MOTS-c is a mitochondrial-encoded interferon-linked host defense peptide
Source: eLife. 2026 Aug 18;12:RP87615. doi: 10.7554/eLife.87615 (PMC13485304; doi:10.7554/eLife.87615)
Supplement: Supplementary file 8 [file elife-87615-fig4-data1.zip › Figure 4_Source Data 1/Figure 4_Source Data 1.pdf]

The Human Mitochondrial Genome Encodes for an Interferon-Responsive Host Defense Peptide

Rice MC, Kim JS, Imun M, Jung SW, Park CY, Lai RW, Barr CR, Son JM, Tor K, Kim E, Lu RJ, Cohen I, Benayoun BA, and Lee C

Fig 4A

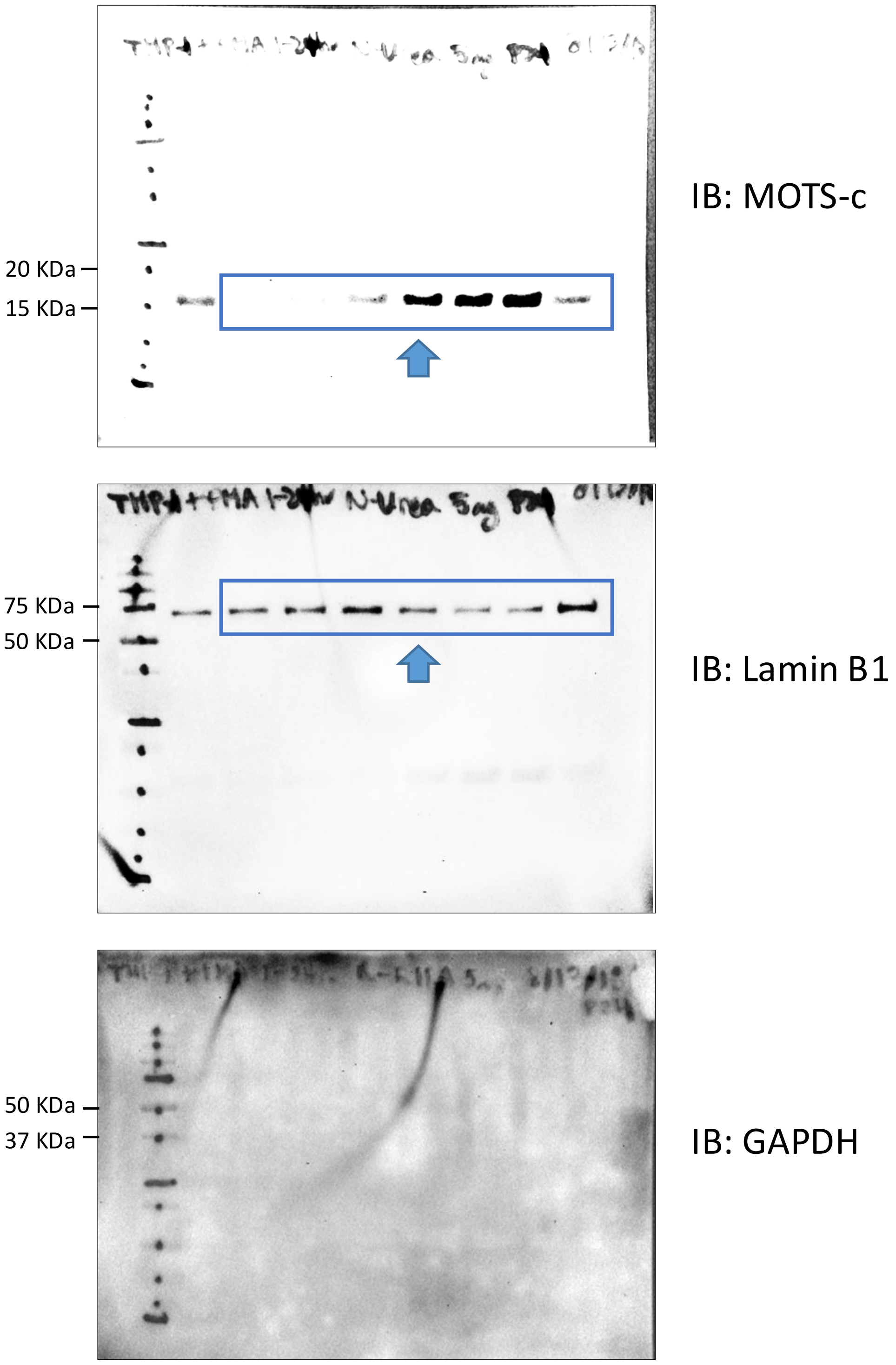

20 KDa
15 KDa
IB: MOTS-c
75 KDa
50 KDa
IB: Lamin B1
50 KDa
37 KDa
IB: GAPDH
