# Supplementary material for: MOTS-c is a mitochondrial-encoded interferon-linked host defense peptide
Source: eLife. 2026 Aug 18;12:RP87615. doi: 10.7554/eLife.87615 (PMC13485304; doi:10.7554/eLife.87615)
Supplement: Peer review history

# Editor report: eLife Assessment

**DOI:** 10.7554/eLife.87615.3.sa0

**Authors:** Florent Ginhoux

**Affiliations:** Singapore Immunology Network Singapore

**Keywords:** Solid, Valuable

This **valuable** study presents findings on the mode of action of MOTS-c (mitochondrial open reading frame from the twelve S rRNA type-c), and its impact on monocyte-derived macrophages. The authors present **solid** evidence for its increased expression in stimulated monocytes/macrophages, its direct bactericidal functions, as well as its role in the modulation of monocyte differentiation into macrophages. Since most of the data were generated from a cell line (THP1), future work is required to validate observations in primary cells and to further support the claims of this work.

# Referee report: Reviewer #1 (Public review)

**DOI:** 10.7554/eLife.87615.3.sa1

In this work, the authors examine the mechanism of action of MOTS-c and its impact on monocyte-derived macrophages. In the first part of the study, they show that MOTS-c acts as a host defense peptide with direct antibacterial activity. In the second part of the study, the authors aim to demonstrate that MOTS-c influences monocyte differentiation into macrophages via transcriptional regulation.

Major strengths. Methods used to study the bactericidal activity of MOTS-c are appropriate and the results convincing.

Major weaknesses. Methods used to study the impact on monocyte differentiation are inappropriate and the conclusions not fully supported by the data shown. A major issue is the use of the THP-1 cell line, a transformed monocytic line which does not mimic physiological monocyte biology. In particular, THP-1 differentiation is induced by PMA, which is a completely artificial system and conclusions from this approach cannot be generalized to monocyte differentiation. The authors would need to perform this series of experiments using freshly isolated monocytes, either from mouse or human. The read-out used for macrophage differentiation (adherence to plastic) is also not very robust, and the authors would need to analyze other parameters such as cell surface markers. It is also not clear whether MOTS-c could act in a cell-intrinsic fashion, as the authors have exposed cells to exogenous MOTS-c in all their experiments. The authors have also analyzed the transcriptomic changes induced by MOTS-c exposure in macrophages derived from young or old mice. While the results are potentially interesting, the differences observed seem independent from MOTS-c and mainly related to age, therefore the conclusions from this figure are not clear. The physiological relevance of this study is also unclear.

# Author comment: Author response

**DOI:** 10.7554/eLife.87615.3.sa2

**Authors:** Michelle C Rice, Maria Imun, Sang Wun Jung, Chan Yoon Park, Jessica S Kim, Rochelle W Lai, Casey R Barr, Jyung Mean Son, Kathleen Tor, Emmeline Kim, Ryan J Lu, Ilana Cohen, Bérénice A Benayoun, Changhan Lee

**Affiliations:** University of Southern California Los Angeles United States; University of Southern California Los Angeles United States; University of Southern California Los Angeles United States; University of Suwon Huaseong-si Republic of Korea; University of Southern California Los Angeles United States; University of Southern California Los Angeles United States; University of Southern California Los Angeles United States; University of Southern California Los Angeles United States; University of Southern California Los Angeles United States; University of Southern California Los Angeles United States; University of Southern California Los Angeles United States; University of Southern California Los Angeles United States; University of Southern California Los Angeles United States; University of Southern California Los Angeles United States

The following is the authors’ response to the original reviews.

**Public Reviews:**

**Reviewer #1 (Public Review):**

In this work, the authors examine the mechanism of action of MOTS-c and its impact on monocyte-derived macrophages. In the first part of the study, they show that MOTS-c acts as a host defense peptide with direct antibacterial activity. In the second part of the study, the authors aim to demonstrate that MOTS-c influences monocyte differentiation into macrophages via transcriptional regulation.

Major strengths.

Methods used to study the bactericidal activity of MOTS-c are appropriate and the results are convincing.

Major weaknesses.

Methods used to study the impact on monocyte differentiation are inappropriate and the conclusions are not supported by the data shown. A major issue is the use of the THP-1 cell line, a transformed monocytic line which does not mimic physiological monocyte biology. In particular, THP-1 differentiation is induced by PMA, which is a completely artificial system and conclusions from this approach cannot be generalized to monocyte differentiation. The authors would need to perform this series of experiments using freshly isolated monocytes, either from mouse or human. The read-out used for macrophage differentiation (adherence to plastic) is also not very robust, and the authors would need to analyze other parameters such as cell surface markers. It is also not clear whether MOTS-c could act in a cell-intrinsic fashion, as the authors have exposed cells to exogenous MOTS-c in all their experiments. The authors did not perform complementary experiments using MOTS-c deficient monocytes. The authors have also analyzed the transcriptomic changes induced by MOTS-c exposure in macrophages derived from young or old mice. While the results are potentially interesting, the differences observed seem independent from MOTS-c and mainly related to age, therefore the conclusions from this figure are not clear. Another concern is the reproducibility of the experiments, as the authors do not indicate the number of biological replicates analyzed nor the number of independent experiments performed.

In this study, we employed the THP-1 cell line as a proof-of-principle to elucidate the existence of a firstin-class mitochondrial-encoded host defense peptide. This peptide is expressed in monocytes and serves dual functions: (i) direct targeting of bacteria, and (ii) regulation of monocyte differentiation. It is noteworthy that THP-1 cells differentiated by PMA have been widely utilized as a model for monocyte differentiation by numerous research groups. While we acknowledge the significance of utilizing primary monocytes to fully comprehend the translational implications of our findings, conducting a complete replication of our experiments in primary monocytes falls beyond the scope of this study. However, we have conducted several pivotal experiments in primary monocytes, including:

i) Demonstration of the induction of endogenous MOTS-c in primary human monocytes during differentiation by M-CSF (Fig 3A).

ii) Observation of an increased number of adhered monocytes during monocyte differentiation following MOTS-c treatment (Fig 5A).

iii) Examination of the transcriptional regulation in mouse primary bone marrow-derived macrophages (BMDMs) by MOTS-c, seven days after a single treatment at the onset of differentiation (Fig 6).

In addition to assessing adherence to plastic, we performed RNA-seq of THP-1 cells during early differentiation with MOTS-c as a measure of accelerated differentiation (Fig 4). The positive correlation between the effects of PMA and PMA+MOTS-c suggests that MOTS-c accelerates the transcriptional changes that occur during differentiation (Fig 4G). We consider this method a more comprehensive evaluation of differentiation as it encompasses the expression of thousands of genes rather than relying on a limited selection of cell surface markers. Future investigations should explore additional indicators of differentiation, including potential epigenetic effects of MOTS-c.

Our findings indicate that endogenous MOTS-c is induced during monocyte stimulation and translocates into the nucleus (Figs 3-4), implying a cell-intrinsic role for MOTS-c during monocyte differentiation. Although examining MOTS-c deficient monocytes would offer valuable insights, technical limitations currently hinder the production of such monocytes due to the mitochondrial genomic encoding of MOTSc within the 12S rRNA.

Furthermore, our study reveals that MOTS-c alters gene expression in macrophages similarly across age and sex groups. This observation, illustrated in Fig 6E where the fold changes in clusters 5 and 6 in response to MOTS-c were consistent across all groups, suggests that MOTS-c modulates macrophage gene expression in an age-related manner. We postulate this to be an adaptive response to age-related alterations in the monocyte and macrophage microenvironment.

The number of biological replicates performed for each experiment is indicated.

The different parts of the manuscript do not appear well connected and it is not clear what the main message from the manuscript would be. The physiological relevance of this study is also unclear.

The main message of our manuscript is that the mitochondrial genome encodes for a previously unknown host defense peptide that has physiological roles in modulating immune responses during infection and during aging. We have edited the ‘introduction’ to clarify this.

**Reviewer #2 (Public Review):**

The research study presented by Rice et al. set out to further profile the host defense properties of the mitochondrial protein MOTS-c. To do this they studied i. the potential antimicrobial effects of MOTS-c on common bacterial pathogens *E. coli* and MRSA, ii. the effects of MOTS-c on the stimulation and differentiation of monocytes into macrophages. This is a well performed study that utilizes relevant methods and cell types to base their conclusions on. However, there appear to be a few weaknesses to the current study that hold it back from more broad application.

Comment 1: From reading the manuscript methods and results, it is unclear exactly what the synthetic MOTS-c source is. Therefore it is hard to determine whether there may be any impurities in the production of this synthetic protein that may interfere with the results presented throughout the manuscript. Though, the data presented in Supplemental Figure 4F, where *E. coli* expressing intracellular MOTS-c inhibited bacterial growth certainly support MOTS-c specific effects. Similarly with the experiments showing endogenous MOTS-c levels rising in stimulation and differentiated macrophages (Figure 3).

We have edited our manuscript to include the source and purity of our synthetic MOTS-c peptide. The MOTS-c peptide used was synthesized by New England Peptides (now Biosynth) with a purity >95% by mass spectrometry.

Comment 2: It is interesting that the mice receiving bacteria coupled with MOTS-c lost about 10% of their body weight. It would have been interesting to demonstrate the cause of this weight loss since the effect appears to be separate from mere PAMPs as shown by using heat-killed MRSA in Supplemental Figure 5. Was inflammation changed? Is this due to changes in systemic metabolism? Would have been interesting to have seen CRP levels or circulating liver enzymes.

As suggested, we repeated this experiment to include both the heat-killed and MOTS-c-MRSA groups in the same controlled experiment for comparison (Fig 2; see below). Blood was collected from these mice for evaluation of cytokine levels and markers of organ damage. While only 1/6 controls survived, all MOTSc and heat-killed MRSA-treated mice survived. However, compared to the heat-killed group, the MOTS-cMRSA group lost more weight and had a higher inflammatory profile, but still significantly less than in the control group. We hypothesize that this is due to only partial killing of MRSA by MOTS-c, as suggested by the CFU plated after overnight incubation, leading to a non-lethal infection in these mice. Others have shown that in this peritonitis model, α-hemolysin production by live MRSA is a key factor in toxicity, rather than PAMP-induced shock (PMID: 8975909; 22802349), which is consistent with the absence of death following heat-killed MRSA inoculation.

Despite these concerns, the data are well suited to answering their research question, and they open up the door to studying how mitochondrial peptides like MOTS-c could have roles outside of the mitochondria.

**Reviewer #1 (Recommendations For The Authors):**

Suggestions for improvement

(1) The authors need to indicate in each legend the number of biological replicates analyzed and the number of independent experiments performed. This is essential.

We have included the number of biological replicates analyzed.

(2) The authors need to repeat the key experiments using freshly isolated monocytes, either human or mouse. THP-1 cells are abnormal cells and findings from these cells cannot be generalized to monocytes. For instance, in Figures 3A and B, it is clear that the kinetics of MOTS-c expression are different between THP-1 cells and human blood monocytes.

The kinetics of THP-1 cells compared to human monocytes are slightly different, as expected by using different cells and different differentiation cues (M-CSF vs PMA). However, our findings collectively demonstrate the same effect, that each stimulus transiently induces the expression of MOTS-c within 24 hours in monocytes.

In Figure 3A, the authors should show what happens in the absence of MCSF. Is MOTS-c expression upregulated by culture alone?

There is some degree of baseline expression of MOTS-c in a resting state, and MOTS-c expression is significantly increased upon stimulation. This expression may be higher in primary monocytes than THP-1 cells, given that these monocytes are inevitably stressed by being removed from the native environment and put through the purification process.

(3) In Figure 4A, a control for cytoplasmic contamination in the nuclear fraction is missing.

We now include GAPDH detection in the nuclear fraction.

**Author response image 1.**

(4) The RNA-seq analysis shown in Figure 4 is not very informative. What genes are differentially expressed? The authors should provide a list of these genes as supplementary information and highlight some key genes in the figure and text.

The complete list of these genes is provided in Tables S1 and S2. We chose not to highlight specific genes in this paper due to the lack of sufficient evidence identifying any particular genes as key factors at this time.

(5) In Figure 5A, a control is missing: the authors should treat the monocytes with the same volume of 'vehicle' (presumably it is water).

In all experiments with MOTS-c treatment, the controls were treated with the same volume of vehicle (water). We have edited legends to state this.

(6) In Figure 6, the differences observed seem independent on MOTS-c. The conclusions from this figure are overstated and need to be rephrased and clarified.

MOTS-c shifted gene expression in macrophages in a similar manner *regardless* of age and sex, as shown in Fig 6E where the fold changes in clusters 5 and 6 in response to MOTS-c were similar in all groups. Independently, aging alone increases the expression of these same genes related to antigen presentation and interferon signaling, suggesting that MOTS-c shifts macrophage gene expression in an age-related manner – the expression of antigen presentation and interferon-related genes have been shown to be highly age-related (PMID: 36040389, 32669714, 36622281, 31754020). We hypothesize this to be an adaptive response to age-related changes in the monocyte and macrophage microenvironment.

(7) Adherence to plastic is not a robust read-out for monocyte differentiation into macrophages. The authors need to examine other parameters, for instance characteristic cell surface markers for macrophages.

As a read-out of accelerated differentiation, in addition to adherence to plastic we performed RNA-seq of THP-1 cells during early differentiation with MOTS-c (Fig 4). The positive correlation between the effects of PMA and effects of PMA+MOTS-c suggest MOTS-c is accelerating the transcriptional changes that occur during differentiation (Fig 4G). We believe this to be a more robust assessment of differentiation as it relies on the expression of thousands of genes rather than a limited selection of cell surface markers. Further studies are needed to assess other read-outs of differentiation, including possible epigenetic effects of MOTS-c.

(8) It is not clear whether MOTS-c could have a cell-intrinsic effect in monocytes. The results should be strengthened by examining the differentiation of monocytes deficient for MOTS-c (without addition of exogenous MOTS-c).

We have shown that endogenous MOTS-c is induced during monocyte stimulation and translocates into the nucleus (Figs 3-4), suggesting that MOTS-c does have a cell-intrinsic role during monocyte differentiation.

While having MOTS-c deficient monocytes would certainly be insightful, because MOTS-c is encoded within the mitochondrial genome in the 12S rRNA there are currently technical limitations in producing these monocytes.

Other points

(1) The paper would benefit from a more extended discussion to understand the physiological relevance of these findings. What cells would release MOTS-c in vivo, and how would that affect monocytes ? Is there a cell-intrinsic of MOTS-c in monocytes, and if so what would be the signals inducing its expression during differentiation ? These aspects should be discussed by the authors so that the readers can understand their views.

We thank the reviewer for their suggestion and have edited the discussion in our revised manuscript.

MOTS-c has been detected in various tissue and cell types, including the liver, muscle, T cells, monocytes/macrophages, and epithelial cells. This aligns with MOTS-c being referred to in literature as a cytokine, which are typically expressed by a broad range of cell types. Consistent with this, we also propose that MOTS-c would be expressed in cells known to express HDPs.

We hypothesize that MOTS-c acts in both a cell-intrinsic and extrinsic manner in vivo, consistent with known HDPs, to both target bacteria directly and modulate immune cell responses. In vitro, M-CSF, PMA, LPS, and IFNγ each induced MOTS-c expression. In vivo, monocytes respond to a range of stimuli that influence their differentiation, and these stimuli may induce MOTS-c as well. We have previously published that MOTS-c acts primarily under conditions of cell stress, such as nutrient deprivation and oxidative stress, to help restore homeostasis. While MOTS-c did regulate macrophage gene expression in resting “M0-like” macrophages, we hypothesize that the physiological role of MOTS-c is to regulate cell adaptation to stress, therefore the context under which monocytes differentiate will be an important factor determining the functional effects of MOTS-c. In future studies, we plan to test whether the immuno-modulatory effects of MOTS-c are dependent on the environment during differentiation.

(2) Scale bar appear to be missing from Figure 1G.

We apologize for the poor resolution of the scale bar. We have made it easily recognizable in the revised figure.

(3) It is not very clear what is shown in Figure S2. The authors should better explain what the images represent.

Figure S2 is related to Figure 1D and Figure S1. In this experiment, *E. coli*, *S. typhimurium*, and *P. aeruginosa* cultures were treated with MOTS-c (100uM). We observed that only *E. coli* aggregated immediately, while

*S. typhimurium* and *P. aeruginosa* did not show aggregation. This suggests that MOTS-c exhibits specificity in targeting certain types of bacteria, although the underlying basis of this specificity is currently unknown.

We have revised the legend as follows: 'MOTS-c exhibits specificity in bacterial targeting. MOTS-c (100 μM) treatment causes immediate aggregation of *E. coli* but not *S. typhimurium* or *P. aeruginosa* (n=6). Representative image shown. See Figure 1D'.

**Reviewer #2 (Recommendations For The Authors):**

This is a beautifully executed study and a well written manuscript. I generally don't have much critical feedback to give based on my reading. The only recommendation I have to improve the completeness of the data would be in relation to Figure 5E and F. The metabolic phenotype of LPS stimulated monocytes/macrophages is more typically the Warburg effect where oxidative phosphorylation is reduced (as you show with a lowered OCR), but with a concomitant elevation in lactate production. It would have been nice to see either i. the ECAR levels from your seahorse data, or ii. separate lactate measurements on your supernatants. This would go a long way to further explaining the phenotype described in the figure.

We greatly appreciate the reviewer's positive feedback. The data provided below are ECAR measurements obtained from the Seahorse assay. However, it's important to note that the assays were originally designed for OCR measurement (*e.g.* buffered media unsuitable for ECAR measurements, use of mitochondrial complex inhibitors, etc.), thus rendering the ECAR data unreliable for accurately assessing glycolysis. Consequently, while we share this data with the reviewer, we believe it is inappropriate to include it in the manuscript (hence omitted in the original submission).

**Author response image 2.** 

Furthermore, we are currently engaged in a separate manuscript focusing on elucidating the immunometabolic mechanisms of MOTS-c in macrophages. We intend for this manuscript to stand alone, providing a comprehensive exploration of metabolic pathways, including a detailed untargeted metabolomics map spanning multiple time-points.
